# Identification of potential candidate vaccines against *Mycobacterium ulcerans* based on the major facilitator superfamily transporter protein

**DOI:** 10.3389/fimmu.2022.1023558

**Published:** 2022-11-08

**Authors:** Tamara Z. Ishwarlall, Victoria T. Adeleke, Leah Maharaj, Moses Okpeku, Adebayo A. Adeniyi, Matthew A. Adeleke

**Affiliations:** ^1^ Discipline of Genetics, School of Life Sciences, University of KwaZulu-Natal, Durban, South Africa; ^2^ Department of Chemical Engineering, Mangosuthu University of Technology, Durban, South Africa; ^3^ Department of Chemistry, Faculty of Natural and Agricultural Sciences, University of the Free State, Bloemfontein, South Africa; ^4^ Department of Industrial Chemistry, Federal University Oye Ekiti, Oye-Ekiti, Ekiti State, Nigeria

**Keywords:** Buruli ulcer, immunoinformatics, multi-epitope-based vaccine, *Mycobacterium ulcerans*, neglected tropical disease

## Abstract

Buruli ulcer is a neglected tropical disease that is characterized by non-fatal lesion development. The causative agent is *Mycobacterium ulcerans (M. ulcerans).* There are no known vectors or transmission methods, preventing the development of control methods. There are effective diagnostic techniques and treatment routines; however, several socioeconomic factors may limit patients’ abilities to receive these treatments. The Bacillus Calmette–Guérin vaccine developed against tuberculosis has shown limited efficacy, and no conventionally designed vaccines have passed clinical trials. This study aimed to generate a multi-epitope vaccine against *M. ulcerans* from the major facilitator superfamily transporter protein using an immunoinformatics approach. Twelve *M. ulcerans* genome assemblies were analyzed, resulting in the identification of 11 CD8^+^ and 7 CD4^+^ T-cell epitopes and 2 B-cell epitopes. These conserved epitopes were computationally predicted to be antigenic, immunogenic, non-allergenic, and non-toxic. The CD4^+^ T-cell epitopes were capable of inducing interferon-gamma and interleukin-4. They successfully bound to their respective human leukocyte antigens alleles in *in silico* docking studies. The expected global population coverage of the T-cell epitopes and their restricted human leukocyte antigens alleles was 99.90%. The population coverage of endemic regions ranged from 99.99% (Papua New Guinea) to 21.81% (Liberia). Two vaccine constructs were generated using the Toll-like receptors 2 and 4 agonists, LprG and RpfE, respectively. Both constructs were antigenic, non-allergenic, non-toxic, thermostable, basic, and hydrophilic. The DNA sequences of the vaccine constructs underwent optimization and were successfully *in-silico* cloned with the pET-28a(+) plasmid. The vaccine constructs were successfully docked to their respective toll-like receptors. Molecular dynamics simulations were carried out to analyze the binding interactions within the complex. The generated binding energies indicate the stability of both complexes. The constructs generated in this study display severable favorable properties, with construct one displaying a greater range of favorable properties. However, further analysis and laboratory validation are required.

## 1 Introduction

Buruli ulcer (BU) is a chronic, necrotizing disease that primarily affects the skin and occasionally bones (1). It is caused by *Mycobacterium ulcerans*, which is categorized as a mycolactone-producing-mycobacterium (MPM) (2). It is classified as a neglected tropical disease (NTD) and is the third most common mycobacterial disease globally, following tuberculosis and leprosy in immunocompetent individuals (3, 4). It has been reported in 33 countries, of which most are in tropical or subtropical regions except for Australia, China and Japan (1). There are 17 known endemic countries, 165 non-endemic countries and 17 previously endemic countries whose current status remains unknown (5). The annual number of globally reported cases remains erratic, as there was a decrease in cases from 2010 to 2016 and a yearly increase until 2018 (1). There was a sharp decline in cases between 2019 and 2020; however, this can be partially attributed to the impact of COVID-19 (1). The World Health Organization (WHO) recommended postponing mass treatment campaigns, active case-finding activities, and population-based surveys for NTDs on the 1st of April 2020, later reaffirming it on the 5th of May 2020 (6, 7). This was done in an attempt to reduce the risk of COVID-19 transmission. The other recommendation issued was that countries should monitor and re-evaluate at regular intervals the necessity for a continuing delay (6). It is important to note the effect this may have had on case detection. Various precautionary measures pertaining to designated sites, health workers, and targeted populations for NTD activities were provided in July 2020 (7). However, it was stated that additional precautionary measures might be developed specifically based nationally or locally (7). Early diagnosis is vital for a positive medical outcome (8). Experienced health professionals are expected to make reliable clinical diagnoses; however, laboratory confirmation is recommended as some BU symptoms are similar to other endemic disease conditions (1).

This disease is characterized by the progression of painless nodules, papules, plaques or edemas to painless ulcers, mainly on arms and legs (9). The painlessness of the disease may be attributed to the effect of mycolactone on specific neurological pathways of the host (10). These ulcers can lead to potential disfigurement or long-term disability if the patient is not treated timeously (8, 9). The recommended treatment consists of 10 mg/kg of rifampicin once daily and 7.5 mg/kg of clarithromycin twice daily (1). However, patients are often required to travel long distances to treatment centers (8). The cost of transportation and accommodation of patients and caregivers is also a burden on affected households (11). Based on the factors mentioned above, focus should be given to prevention methods. There are no confirmed vectors or reservoirs of *M. ulcerans*, which hinders the development of prevention methods (12). Vaccination is a promising avenue that may limit further infections.

The Bacillus Calmette–Guérin (BCG) vaccine was created against tuberculosis using live attenuated *M. bovis* (13). BCG was tested against *M. ulcerans* infection due to its ability to induce significant cross-reactive immune responses against other mycobacteria (14). However, it could not induce long-term protection in humans (15, 16). Vaccines generated specifically against *M. ulcerans* ranged from the use of live *M. ulcerans* or other mycobacterial species to DNA vaccines (17). None of these vaccines could generate long-term protective immune responses in animal models and were not entered into clinical trials (17). The advent of technology has allowed for the movement of vaccine design from using purely laboratory-based methods to the amalgamation of both laboratory- and computer-based approaches.

One of these approaches is reverse vaccinology (RV). RV has been steadily evolving since the development of sequencing technologies (18). This method has been called ‘a new way of thinking to vaccine development’, and the era of RV has been termed ‘a renaissance of vaccinology’ (19, 20). It entails the use of various computational methods and tools to identify vaccine candidates consisting of surface or secreted proteins that may induce a protective response in the host (21, 22). The identification and study of proteins linked to pathogenesis may yield promising new strategies for therapeutic intervention (23). Another promising avenue is the use of anti-mycobacterial peptides, which are observed to inhibit synthesis, interfere with the cell membrane or envelope and have immunomodulatory activity (24, 25). Minimal immunogenic regions of these protein antigens, known as epitopes, may be used to form a multi-epitope vaccine (MEV) (26, 27). This approach proves advantageous in terms of the increased speed and lowered cost when identifying potential candidates, especially for bacterial diseases, as culturing bacteria is unnecessary at the identification stage of the study (28). The lack of the use of the whole microorganism could decrease the risk of side effects while potentially inducing a protective immune response (29).

The adaptive immune system is comprised of cytotoxic and humoral immune responses (27). T-cell immunity is vital to consider during vaccine design as the resulting neutralizing antibodies are critical for the success of the vaccine, and a cellular response against conserved antigens may yield a broader protective response against multiple strains of the pathogen (30). T-cells can be broken down into two main types, i.e., cytotoxic lymphocytes (CTLs) and T-helper cells (T_H_ cells) (27). CTLs, also known as CD8^+^ cells, destroy infected cells through direct cytotoxic action aided by T_H_ cells, known as CD4^+^ cells (27). CD4^+^ cells also play a vital role in the expansion, differentiation, class switching and affinity maturation of B-cells and their responses (31). B-cells are critical in the facilitation of the secretion of antibodies and the mediation of the humoral adaptive immunity (32). The identification of epitopes recognized by CD8^+^ and CD4^+^ T-cells can be used to identify new antigens (31).

There have been two studies involving the identification of T- and B-cell epitopes from different types of *M. ulcerans* proteins (33, 34). The earlier study identified epitopes from the Proline-Glutamate Polymorphic GC-rich Sequence (PE-PGRS) protein of *M. ulcerans strain Agy 99* (34), while the second study identified epitopes from virulence factors of *M. ulcerans strain Agy99* (33). The major facilitator superfamily (MFS) transporters are involved in a wide variety of physiological processes, with different subfamilies playing vital roles in every kingdom of life (35). The aim of this study is to identify and analyze antigenic CD8^+^ and CD4^+^ T-cell and B-cell epitopes from the MFS transporter proteins from *M. ulcerans.* These epitopes will be analyzed to determine if they may be capable of inducing a protective immune response in an *in-silico* MEV using similar immunoinformatic tools and webservers.

## 2 Materials and methods

### 2.1 Identification of possible virulent outer membrane peptides

The analysis of the entire proteome may broaden the number of epitopes that may be identified compared to specific narrow searches. Twelve *M. ulcerans* genome assemblies were downloaded from the National Center for Biotechnology Information database (NCBI) (https://www.ncbi.nlm.nih.gov/) in the protein format (**Supplementary Table 1**). The proteomes were combined and submitted to the MP3: Prediction of Pathogenic/Virulent Proteins database (http://metagenomics.iiserb.ac.in/mp3/index.php), with the threshold set to 0.5 (36). The MP3 tool uses an integrated Support Vector Machine (SVM)-Hidden Markov Model (HMM) approach to accurately predict potentially pathogenic proteins (36). MFS proteins that were identified as virulent were extracted and submitted to Clustal Omega (https://www.ebi.ac.uk/Tools/msa/clustalo/) for multiple sequence alignment (37, 38). The aligned proteins were submitted to the TMHMM v 2.0 webserver (https://services.healthtech.dtu.dk/service.php?TMHMM-2.0) for topology analysis (39, 40). Only proteins that were identified as having an outer topology were selected.

### 2.2 Prediction of T-cell epitopes

#### 2.2.1 Prediction of CD8^+^ epitopes

The probable outer membrane proteins were submitted to NetMHCpan v 4.1 (https://services.healthtech.dtu.dk/service.php?NetMHCpan-4.1), with the peptide length set to 9 (41). NetMHCpan v 4.1 uses artificial neural networks (ANNs) to predict peptides that may bind to major histocompatibility complex molecules (MHC) of known sequences (41). The human leukocyte antigens (HLA) alleles within endemic countries on Allele Frequency Net Database (http://www.allelefrequencies.net/default.asp) were filtered based on the allele frequency, in order of highest to lowest, and the population standard was set to gold only (**Supplementary Table 2**) (42). The first ten respective HLA alleles were selected for each available allele type and combined with more HLA alleles (**Supplementary Table 3**). This combination of alleles was used to generate MHC I binding molecules based on the availability of HLA molecules on the website. Nonamers with IC_50_ values ≤ 250 nM were extracted and submitted to VaxiJen v 2.0 (http://www.ddg-pharmfac.net/vaxijen/VaxiJen/VaxiJen.html) for antigenicity analysis (43). VaxiJen v 2.0 classifies antigens based on the physicochemical properties of the peptides (43). The target organism selected was bacteria, and the threshold was set to 0.5. Only antigenic nonamers (≥ 0.5) were selected and submitted to the Class I Immunogenicity website (http://tools.iedb.org/immunogenicity/) (44). This website analyses peptides by examining its amino acid properties and positions, resulting in the prediction of the immunogenicity of the peptide-MHC complex (44). Peptides with positive scores were extracted.

#### 2.2.2 Prediction of CD4^+^ epitopes

The probable outer membrane proteins were submitted to NetMHCIIpan v 4.0 (https://services.healthtech.dtu.dk/service.php?NetMHCIIpan-4.0), with the peptide length set to 15 (45). NetMHCIIpan v 4.0 also uses ANNs to predict peptides capable of binding to MHC II molecules of known sequences (45). The first ten respective HLA alleles from the endemic populations in order of highest to lowest allele frequency were selected from the Allele Frequency Net Database (http://www.allelefrequencies.net/default.asp) (**Supplementary Table 2**) (42). Only populations of a gold standard were selected. These HLA-alleles were combined with another set of HLA-alleles and used to generate MHC II sequences based on the available HLA molecules on the website (**Supplementary Table 3**). Peptides with an IC_50_ value ≤ 250 nM were extracted. They were submitted to VaxiJen v 2.0 (http://www.ddg-pharmfac.net/vaxijen/VaxiJen/VaxiJen.html) for antigenicity analysis (43). The target organism selected was bacteria, and the threshold was set to 0.5. Only antigenic peptides (≥ 0.5) were selected.

### 2.3 Assessment of cytokine-induction, allergenicity, toxicity, and conservancy properties of CD4^+^ epitopes

The antigenic CD4^+^ epitopes were submitted to the IFNepitope website (http://crdd.osdd.net/raghava/ifnepitope/predict.php) (46). Epitopes that were positive for inducing interferon-gamma (IFN-γ) were selected. They were then submitted to IL4pred (https://webs.iiitd.edu.in/raghava/il4pred/predict.php) to predict epitopes that may be capable of inducing interleukin-4 (IL-4) (47). The SVM threshold was set to the default. Epitopes predicted to induce IL-4 were extracted. The immunogenic CD8^+^ and IL-4 inducing CD4^+^ epitopes were submitted to the AllerTOP v. 2.0 webserver (https://www.ddg-pharmfac.net/AllerTOP/) for allergenicity analysis (48). AllerTop uses the auto cross-covariance (ACC) protein mining method to classify peptides as allergens or nonallergens (48, 49). Epitopes predicted to be non-allergenic were batch submitted to ToxinPred (https://webs.iiitd.edu.in/raghava/toxinpred/index.html) (50, 51). Non-toxin T-cell epitopes were identified and extracted. The non-toxin CD8^+^ and CD4^+^ epitopes were submitted to the Epitope Conservancy Analysis website (http://tools.iedb.org/conservancy/) (52). The Epitope Conservancy Analysis website calculates the degree of conservancy of an epitope with a given protein sequence at a given identity level (52). It defines conservancy as the fraction of protein sequences that contain the epitope (52). CD8^+^ epitopes with 100% linear conservancy were selected and used to identify overlapping CD4^+^ epitopes.

### 2.4 Population coverage analysis

A total of 11 CD8^+^ and 7 CD4^+^ epitopes were submitted to the Population Coverage website (http://tools.iedb.org/population/) (53). The Population Coverage website determines the fraction of individuals predicted to respond to a given epitope based on HLA genotypic frequencies, MHC I and II binding, and T-cell restriction data (53). This is done to attempt to prevent ethnically biased population coverage (53). The respective HLA alleles were inputted for both the CD8^+^ and CD4^+^ T-cell epitopes (**Supplementary Table 4**). The calculation option was set to Class I and II combined. The endemic regions were chosen as per the WHO database (5). The following regions were selected, World, Australia, Cameroon, Central African Republic, Congo, Côte d’Ivoire, Ghana, Gabon, Japan, Liberia, Nigeria, Papua New Guinea, Sudan, and West Africa.

### 2.5 Prediction of B-cell epitopes

The probable outer membrane peptides were submitted to ABCpred (https://webs.iiitd.edu.in/raghava/abcpred/index.html) with default parameters (54, 55). The linear B-cell epitopes identified (≥ 0.51) were extracted and submitted to VaxiJen v 2.0 (http://www.ddg-pharmfac.net/vaxijen/VaxiJen/VaxiJen.html) (43). Bacteria was selected as the target organism, and the threshold was set to 0.5. Antigenic B-cell epitopes (≥ 0.50) were extracted. B-cell epitopes that were 100% conserved with any of the 11 CD8^+^ epitopes were identified with the Epitope Conservancy Analysis website (http://tools.iedb.org/conservancy/) (52). CD8^+^ epitopes with 100% conservancy were selected and used to identify overlapping B-cell epitopes. The conserved B-cell epitopes were submitted to the AllerTOP webserver (https://www.ddg-pharmfac.net/AllerTOP/) for allergenicity analysis and ToxinPred (https://webs.iiitd.edu.in/raghava/toxinpred/index.html) for toxicity analysis (48, 50, 51). Two non-allergenic and non-toxic B-cell epitopes were identified and extracted. Upon identification T-cell epitopes, the source proteins were identified. The T-cell epitopes were submitted to ImmunomeBrowser (http://tools.iedb.org/immunomebrowser/) to determine if the identified epitopes have been examined in immune assay studies, and if so, the results of the assays (56). The parameters were set to default.

### 2.6 Molecular docking of CD8^+^ and CD4^+^ epitopes

Molecular docking of the T-cell epitopes to the active sites of their respective HLA alleles was carried out to determine if interactions between the epitopes and the respective MHC would occur (57). The most conserved HLA allele for the CD8^+^ epitopes with an existing crystalline structure was HLA-A*02:06. The most common HLA allele for the virulent CD4^+^ epitopes was HLA-DRB1*01:01. The crystalline structures 3OXR was retrieved for HLA-A*02:06 (58, 59) and 1T5X for HLA-DRB1*01:01 (60, 61) from the RCSB Protein Data Bank (PDB) (https://www.rcsb.org/) (62, 63). The structures were cleaned using UCSF Chimera v.1.14 (64), and chain A for HLA-A*02:06 and chains A and B for HLA-DRB1*01:01 were selected for docking. The binding sites of all the alleles structures were identified for solvent accessibility and flexibility with the aid of the Naccess 2.1.1 package (65). The allele structures and the predicted epitopes were submitted to ATTRACT Online (http://www.attract.ph.tum.de/services/ATTRACT/peptide.html) for docking analysis. The docking was completed on the locally installed ATTRACT on Centre for High-Performance Computing (CHPC) South Africa. Following docking, 50 frames were generated for each docking interaction. The best frame for each docking model was determined based on the lowest energy value. Visual Molecular Dynamics (VMD) software v 1.9.3 was used to visualize the best model for each epitope, and UCSF Chimera v.1.14 was used to produce images of the epitope structures (64, 66).

### 2.7 Construction of the multi-epitope vaccine candidate sequences and structural analysis

#### 2.7.1 Generation of the multi-epitope vaccine models

The 11 CD8^+^ and 7 CD4^+^ T-cell epitopes and 2 B-cell epitopes that were antigenic, immunogenic, non-allergenic and non-toxic were assembled to form two candidate sequences. The TLR2 and -4 agonists Lipoprotein LprG and the resuscitation-promoting factor (RpfE), respectively, were selected as adjuvants to boost the potential resulting immune response (67, 68). The sequences for LprG (accession number ABL04283.1) and RpfE (accession number OIN23277.1) were retrieved from NCBI (https://www.ncbi.nlm.nih.gov/). The LprG adjuvant was added to construct one, and the RpfE adjuvant was added to construct two. The EAAAK linker was used to connect the respective adjuvants at the N-terminal of the vaccine constructs, the AAY linker between the CD8^+^ epitopes, the GPGPG linker between the CD4^+^ epitopes and the KK linker between the B-cell epitopes. The CD8^+^ and CD4^+^ epitopes were arranged in decreasing order of antigenicity values. The two models were submitted to AllerTOP v. 2.0 (https://www.ddg-pharmfac.net/AllerTOP/) for allergenicity analysis (48). They were then submitted to VaxiJen v 2.0 (http://www.ddg-pharmfac.net/vaxijen/VaxiJen/VaxiJen.html), with a threshold of 0.5 (43).

#### 2.7.2 Structural analysis of the relevant vaccine candidate sequences

The analysis of secondary and tertiary structures is critical when designing vaccines (69). The constructs were submitted to the Gor IV Secondary Structure Prediction Method webserver (https://npsa-prabi.ibcp.fr/cgi-bin/npsa_automat.pl?page=/NPSA/npsa_gor4.html) to predict the secondary structures (70, 71). The construct sequences were then analyzed by the trRosetta algorithm (https://yanglab.nankai.edu.cn/trRosetta/), and 3D models were generated (72–74). The best models were chosen and refined using the GalaxyRefine tool on the GalaxyWeb website (https://galaxy.seoklab.org/cgi-bin/submit.cgi?type=REFINE) (75, 76). Model refinement was carried out to improve the structural quality of the two vaccine structures. The best models were selected for each construct based on their conformation. ProSA-web (https://prosa.services.came.sbg.ac.at/prosa.php) was used to validate the refined models by calculating the Z-scores (77, 78). The models were then submitted to the SAVES v6.0 website (https://saves.mbi.ucla.edu/) for analysis and further validation using ERRAT and PROCHECK (79–81).

#### 2.7.3 Physicochemical analysis of the vaccine candidate sequences

Through the computational analysis of physicochemical properties of proteins, one can understand the functions of the protein encoded by genes *in vitro* (82). The physical and chemical parameters of the vaccine models were analyzed using the ProtParam tool (https://web.expasy.org/protparam/) (83). This included the molecular weight, amino acid composition, isoelectric point, instability index, aliphatic index, the grand average of hydropathicity (GRAVY) and the estimated half-life of the protein in mammalian cells, yeast cells, and *Escherichia coli* (83). The models were submitted to the SCooP v 1.0 website (http://babylone.ulb.ac.be/SCooP/index.php) to determine various thermodynamic properties of the structures, such as the melting temperature (T_m_), change in enthalpy (Δ H_m_), change in specific heat upon folding (Δ C_p_), and the folding free energy at room temperature (Δ G_r_) (84–86).

#### 2.7.4 *In-silico* codon adaptation, vaccine optimization and expression

The models were submitted to the JAVA Codon Adaption Tool (JCat) (http://www.jcat.de/) for codon adaptation and vaccine optimization (87). Codon adaptation is critical to avoid the expression of rarely employed codons in the host, which can lead to poorly translated mRNA, decreased mRNA stability, the possible premature termination of translation and the misincorporation of amino acids (87). JCat determines the optimized sequence’s Codon Adaptation Index (CAI) and GC content (%). The model organism was set to *Escherichia coli* (strain K12). The XhoI (5’CTCGAG’3) and HindIII (5’AAGCTT’3) restriction sites were added to the N- and C-terminals of the first DNA optimized sequence, respectively. The HindIII (5’AAGCTT’3) and BamHI (5’GGATCC’3) restriction sites were added to the N- and C-terminals of the second DNA optimized sequence, respectively. The sequences were each inserted into the pET-28a(+) plasmid using SnapGene v.6.0.2 software (from Insightful Science; available at https://www.snapgene.com/).

#### 2.7.5 Molecular docking of the multi-epitope vaccine sequences to toll-like receptors

The crystalline structures 3A7B (88, 89) and 4G8A (90, 91) for TLR2 and TLR4 were downloaded from RCSB PDB (https://www.rcsb.org/), respectively (62, 63). The structures were cleaned and prepared using UCSF Chimera v.1.14 (64). The docking method followed that of the CD8^+^ and CD4^+^ T-cell epitopes.

### 2.8 Structural analyses of the TLR– multi-epitope vaccine complexes

The flexibility of proteins impacts the structures’ ability to respond to chemical modifications, environmental changes, and ligand binding (92). The two MEV-TLR complexes were submitted to the CABSflex v. 2.0 website (http://biocomp.chem.uw.edu.pl/CABSflex2) for the analysis of the flexibility of the structures (93). Interface residues in protein-protein interactions contribute to the stability and specificity of a complex (94). The complexes were sent to ProFunc (https://www.ebi.ac.uk/thornton-srv/databases/profunc/) to analyze the binding interactions between the TLRs and MEV complexes (95). The results were viewed using PDBsum (96). The solubility and aggression propensity of the complex was examined using AGGRESCAN3D v. 2.0 (http://212.87.3.12/A3D2/) (97, 98).

### 2.9 Immune simulations

The two MEV candidate sequences were submitted to C-IMMSIM (https://kraken.iac.rm.cnr.it/C-IMMSIM/index.php?page=1) to observe the simulation of the potential immune response to the designed constructs (99, 100). The settings were kept at default, with a time step of 1 and a single injection with no lipopolysaccharide (LPS) selected.

### 2.10 Molecular dynamics simulations

The docked complexes, bound, and unbound MEV constructs underwent molecular dynamic simulations (MDS) using the AMBER 14 and 18 packages (101, 102). This was carried out to evaluate the stability of the complex and the interactions between the proteins (103). The proteins were described using FF14SB (104). The topologies were generated using the LEaP module of AMBER 14 (101). Protons and Na^+^ ions were added as counter ions to the complexes, and Cl^-^ was added to the unbound MEV constructs. This was done to neutralize the system in an orthorhombic box of TIP3P water molecules of 8 Å (105). Initial energy minimization was carried out for 10 000 steps (500 steepest descents with 9500 conjugate gradient), after which full energy minimization was carried out for 2000 steps. The complexes were gradually heated from 0 K to 300 K in a canonical ensemble (NVT) with a Langevin thermostat for 2 ns. The collision frequency applied to the system was 1.0 p s^−1^, with the density of the water system regulated with 2 ns of NPT (constant number N, pressure P and time T) simulation. The complexes were equilibrated at 300 K for an additional 2 ns at a pressure of 1 bar. MDS production was run for 100 ns at NVT. The simulations were run using the GPU (CUDA) version of PMEMD provided in AMBER 18 (102, 106–108).

### 2.11 Post molecular dynamics simulations analysis

The CPPTRAJ and PTRAJ modules in AMBER 18 were used to carry out post-MDS analyses (102, 109). The Root Mean Square Deviation (RMSD) and the Root Mean Square Fluctuations (RMSF) of the complexes and the MEV constructs were determined. The Molecular Mechanics/Generalized Born Surface Area (MM/GBSA) module in AMBER 18 was used to calculate the endpoint binding free energy of the docked complexes using the formula:


$$\Delta G_{bind}={\text{G}}_{complex}-\left(G_{receptor}+G_{ligand}\right)$$

The CPPTRAJ and PTRAJ modules were used to generate 2000 frames of the complexes and the unbound MEVs (109). VMD v 1.9.3 was used to view the generated structure (66). The Bio3D package was loaded onto RStudio v 4.0.4 and used to perform principal component analysis (PCA) and cross-correlation analysis (110, 111). PCA was performed to generate information regarding the nature of the clusters and conformational changes following MDS (112). Cross-correlation analysis generates a dynamical cross-correlation matrix (DCCM) and is used to determine the extent to which the fluctuations within the complexes and MEVs are correlated (110). This is done by analyzing the pairwise cross-correlation coefficients (110).

## 3 Results

### 3.1 Epitope analyses

#### 3.1.1 Identification of potential virulent outer membrane peptides

A total of 9906 potentially virulent proteins were identified. There were 9 MFS transporter proteins identified as virulent. After alignment, 97 peptides of various lengths were identified. There were 49 peptides identified to have an outer topology.

#### 3.1.2 Identification of T-cell epitopes, cytokines, and conservancy

The screening of CD8^+^ and CD4^+^ T-cell epitopes capable of binding to MHC I and II yielded 178 CD8^+^ and 245 CD4^+^ T-cell epitopes. Following antigenicity analysis, 80 CD8^+^ and 89 CD4^+^ antigens were identified. A total of 62 immunogenic CD8^+^ T-cell epitopes were identified and extracted. CD4^+^ epitopes capable of inducing IFN-γ and IL-4 amounted to 30 and 10, respectively. A total of 47 CD8^+^ and 7 CD4^+^ non-allergenic and non-toxic epitopes were detected. Upon conservancy analysis, 11 conserved CD8^+^ epitopes and 7 overlapping CD4^+^ epitopes were identified. The CD8^+^ epitope GVDGRLPLL had the highest antigenicity score, i.e., 1.80, while the epitope with the lowest antigenicity was YAQRAAHRL, with a score of 0.64 (**Supplementary Table 4**). The IC_50_ values for the CD8^+^ epitopes ranged from 6.16nM (FLWGVDGRL) to 246.48nM (YAQRAAHRL) (**Supplementary Table 4**). The CD4^+^ epitope WAGFLWGVDGRLPLL had the lowest antigenic score of 0.78 and GSAPVVGVNPWAITL with the highest antigenic score of 1.67 (**Supplementary Table 4**). The IC_50_ values for the CD4^+^ epitopes ranged from 17.17nM to 242.18nM for PFALRLIRPAWQRPV and GSAPVVGVNPWAITL, respectively (**Supplementary Table 4**). The CD8^+^ and CD4^+^ epitopes were identified to originate from the 4 MFS transporter proteins (**Supplementary Table 5**). The highest virulence score was 1.02 (EUA85589.1), and the lowest virulence score was 0.69 (WP_096369848.1). The ImmunomeBrowser was used to computer specific T-cell response frequency (RF) with the lower and upper bound confidence interval (CI). All the epitopes showed positive responses (**Supplementary Table 6**) and good response frequency was observed for CD4^+^ T-cell epitopes indicating more immunodominant regions than CD8^+^ T-cell epitopes.

#### 3.1.3 Population coverage of the CD8^+^ and CD4^+^ epitopes

**Figure 1:**
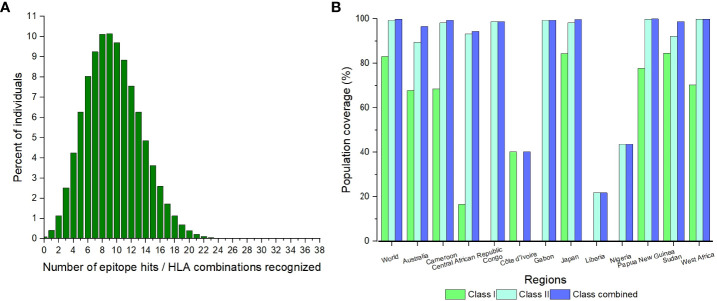
The population coverage of the CD8^+^ and CD4^+^ epitopes for the world and identified endemic regions. **(A)** The number of epitopes hits/HLA combinations recognized by different percentages of individuals globally. **(B)** The population coverage of the individual classes and combined class for the significant regions.

The highest number of epitope hits/HLA combinations recognized by the greatest percentage of individuals was 9 combinations recognized by 10.14% of individuals (**Figure 1A**). The lowest number of combinations recognized was 26 to 38 by 0% of individuals (**Figure 1A**). There was a great variation in the population coverage among the endemic countries. The country with the highest combined class population coverage was Papua New Guinea, with a percentage of 99.99%, and the lowest was Liberia, with a population coverage of 21.81% (**Figure 1B** and **Supplementary Table 7**). The global individual class I and II and class combined HLA coverage values were 83.15%, 99.40% and 99.90%, respectively (**Supplementary Table 7**).

#### 3.1.4 Identification of B-cell epitopes

A total of 34 B-cell epitopes were initially identified. Upon antigenicity analysis, 14 antigenic B-cell epitopes were detected. There were 11 epitopes that were determined to be non-allergenic and non-toxic. Once overlapped with the 11 CD8^+^ T-cell epitopes, 2 B-cell epitopes were identified. The first B-cell epitope (LPGCDSRYAQRAAHRL) had an ABCpred score of 0.56 and an antigenicity score of 0.78, while the second epitope (VGVNPWAITLAVSLAV) had an ABCpred score of 0.60 and antigenicity score of 1.45.

#### 3.1.5 Molecular docking of CD8^+^ and CD4^+^ epitopes

**Figure 2:**
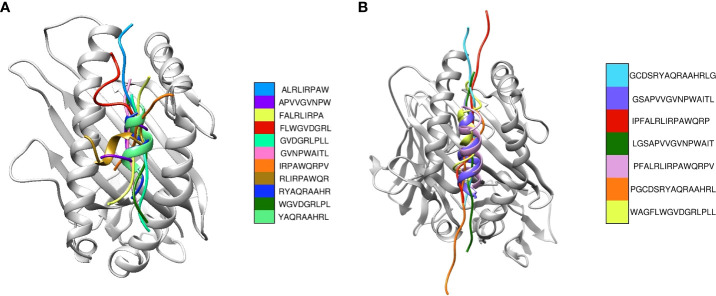
Superimposition of the most suitable epitopes. **(A)** The CD8^+^ epitopes are superimposed, with each colour representing a different model. The respective HLA structures are shown in grey **(B)** The CD4^+^ epitopes are superimposed, with each colour representing a different model. The HLA structure is shown in grey.

Upon the completion of molecular docking, the binding energy of the CD8^+^ epitopes interacting with HLA-A*02:06 ranged from -132.37 to -93.27 kCal/mol (**Table 1**). The CD4^+^ epitopes interacting with HLA-DRB1*01:01 had binding energies ranging from -186.49 to -88.17 kCal/mol (**Table 1**). **Figures 2A, B** displayed the binding of the epitopes in the binding pockets of the respective HLAs.

**Table 1:** The CD8^+^ and CD4^+^ epitopes, along with the best energy values.

Epitope	Energy (kCal/mol)
**CD8^+^ Epitopes**	
ALRLIRPAW	-106.74
APVVGVNPW	-93.27
FALRLIRPA	-113.41
FLWGVDGRL	-111.71
GVDGRLPLL	-116.19
GVNPWAITL	-114.19
IRPAWQRPV	-115.51
RLIRPAWQR	-115.42
RYAQRAAHR	-116.13
WGVDGRLPL	-132.37
YAQRAAHRL	-97.27
**CD4^+^ Epitopes**	
GCDSRYAQRAAHRLG	-186.49
GSAPVVGVNPWAITL	-88.17
IPFALRLIRPAWQRP	-172.70
LGSAPVVGVNPWAIT	-120.44
PFALRLIRPAWQRPV	-169.27
PGCDSRYAQRAAHRL	-144.63
WAGFLWGVDGRLPLL	-131.30

### 3.2 Multi-epitope vaccine analyses

#### 3.2.1 Construction of the MEV candidate sequences and structural analysis of the candidate sequences

**Figure 3:**
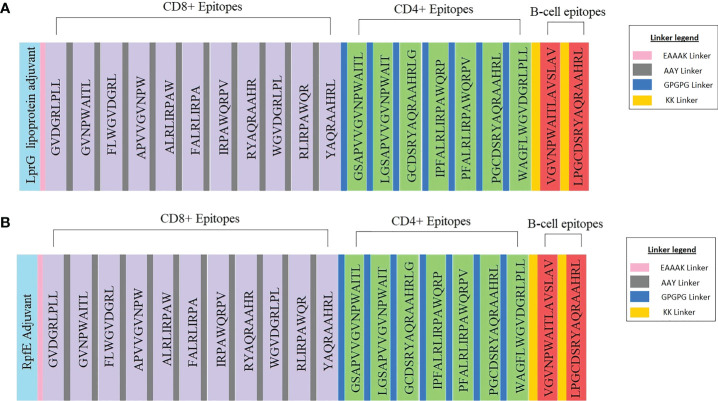
Schematic representation of the multi-epitope vaccine constructs with the legend for the linkers. **(A)** Vaccine construct one consists of the LprG adjuvant, CD8^+^, and CD4^+^ T-cell epitopes, B-cell epitopes and the respective linkers. **(B)** Vaccine construct two consists of the RpfE adjuvant, CD8^+^, CD4^+^ T-cell epitopes, B-cell epitopes and the respective linkers.

The vaccine constructs one, and two consisted of 546 and 524 amino acids, respectively (**Figures 3A, B**). The antigenicity value of vaccine constructs one and two were predicted to be 0.84 and 0.86, respectively (**Figures 3A, B**). Vaccine construct one consisted of 183 alpha-helices, 82 extended strands and 281 random coils (**Supplementary Figure 1A**). The second vaccine construct had 148 alpha helices, 66 extended strands and 310 random coils (**Supplementary Figure 1B**). Both constructs were non-allergenic and non-toxic. The 3D models of the two constructs were generated (**Supplementary Figures 2A, B**) The Z-score of the refined vaccine models was -6.04 and -4.64, respectively (**Supplementary Figures 3A**, **4A**). A total of 97.90% of residues from vaccine model one was in the most favoured regions, and 2.10% was in the additional allowed regions of the generated Ramachandran plot (**Supplementary Figure 3B**). Vaccine model two consisted of 99.20% residues in the most favoured regions and 0.80% in the additional allowed regions within the Ramachandran plot (**Supplementary Figure 4B**). The overall quality factors were 95.72 for vaccine construct one and 94.12 for vaccine construct two (**Supplementary Figures 3C**, **4C**).

#### 3.2.2 Physicochemical analysis of the vaccine candidate sequences

The sequence of vaccine candidate one consisted of 8155 atoms, with a molecular weight of 57.45 kDa. It contained 29 negatively charged residues and 58 positively charged residues. The theoretical isoelectric point (pI) value was estimated to be 10.30, and the instability index was 27.10. The aliphatic index and the GRAVY value of the first construct were determined to be 89.30 and -0.06, respectively. This indicates that the first construct is basic, stable, thermostable and hydrophilic. The extinction coefficient was estimated to be 118830-1 cm-1 at 280 nm measured in water, assuming all cysteine residues are reduced and 119080-1 cm-1 at 280 nm measured in water, assuming all pairs of cysteine residues form cystines. Vaccine construct two consisted of 7659 atoms with a molecular weight of 54.51 kDa. The second vaccine construct contained 36 negatively charged residues and 48 positively charged residues. The pI value, instability index, aliphatic index and GRAVY value for the second construct were 9.58, 42.05, 78.45 and -0.13, respectively. The second construct is also basic and hydrophilic; however, it was unstable and less thermostable than the first construct. The extinction coefficient was estimated to be 124330M-1 cm-1 at 280 nm measured in water, assuming all cysteine residues are reduced and 124580-1 cm-1 at 280 nm measured in water, assuming all pairs of cysteine residues form cystines. The N-terminal amino acid was considered to be methionine for both constructs. The half-life of both vaccine constructs was approximately 30 hours (in mammalian reticulocytes, *in vitro*), less than 20 hours (yeast, *in vivo*) and less than 10 hours (*Escherichia coli*, *in vivo*). Based on the generated Gibbs-Helmholtz curve for the first construct, the Δ Hm, Δ Cp, Tm and Δ Gr values were 23.60 kCal/mol, 0.72 kcal/(mol K), 61.30° C, and 1.1 kCal/mol, respectively (**Supplementary Figure 5A**). The Δ Hm, Δ Cp, Tm and Δ Gr values for the second construct were 119.90 kCal/mol, 3.56 kcal/(mol K), 63° C, and 5.60 kCal/mol, respectively (**Supplementary Figure 5B**).

#### 3.2.3 Codon adaptation, vaccine optimization and cloning

**Figure 4:**
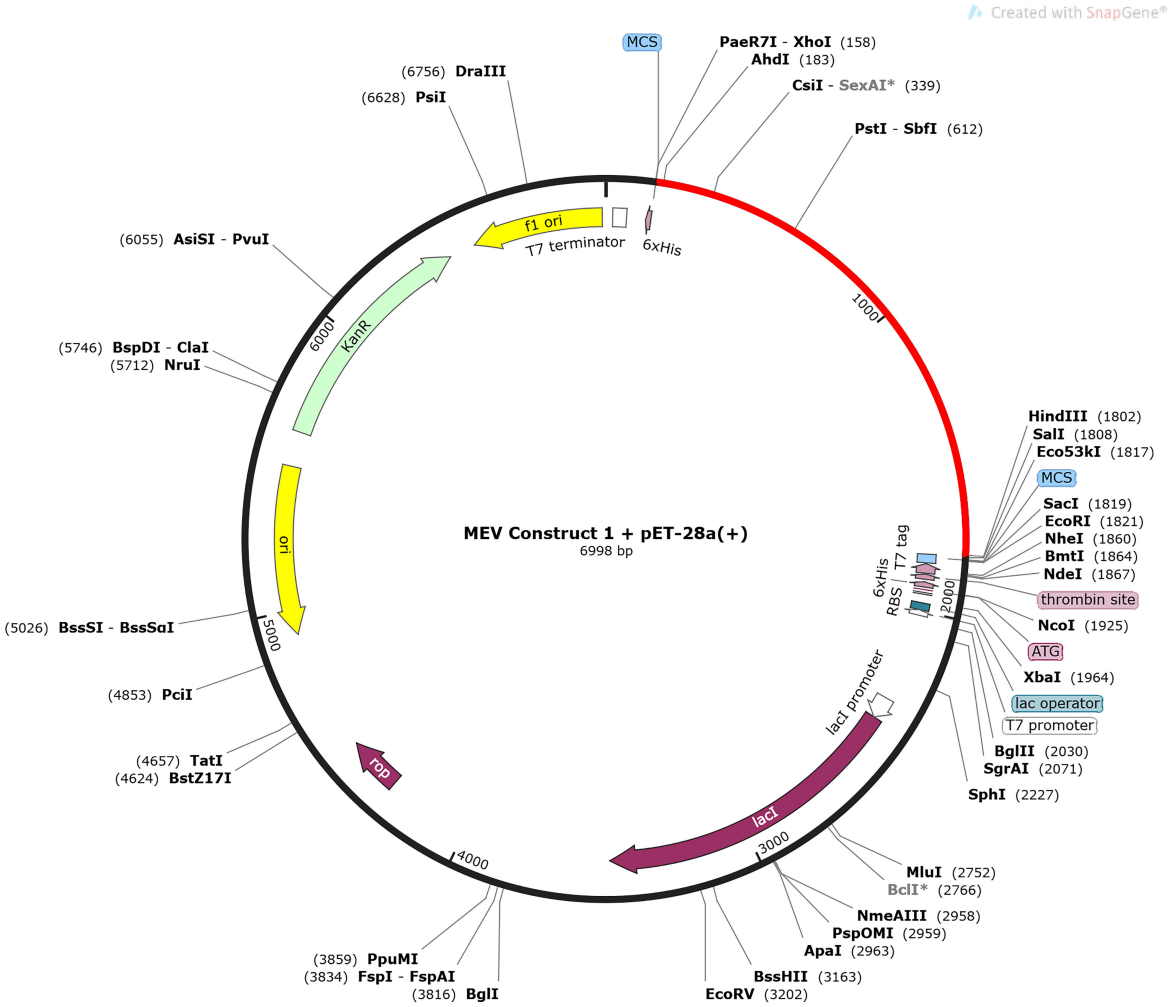
The *in-silico* cloning map of the pET-28a(+) plasmid, with the optimized DNA sequence of the first MEV construct shown in red. The sequence is located between XhoI (158) and HindIII (1802).

The CAI value of both vaccine constructs was estimated to be 1 (**Supplementary Figures 6A, B**). However, construct one had an average GC content of 59.46%, while the average content of construct two was 62.85%. Both constructs fell within the optimal range of the CAI value and GC content, indicating an improved expression of the genes in *E. coli* without translation errors. The combined first vaccine construct and the pET-28a(+) plasmid amounted to 6998 bp, while the second vaccine construct and the plasmid amounted to 6922 bp (**Figure 4** and **Supplementary Figure 7**).

#### 3.2.4 Binding interaction of constructed multi-epitope to the TLRs and structural analysis of the TLR-MEV complexes

**Figure 5:**
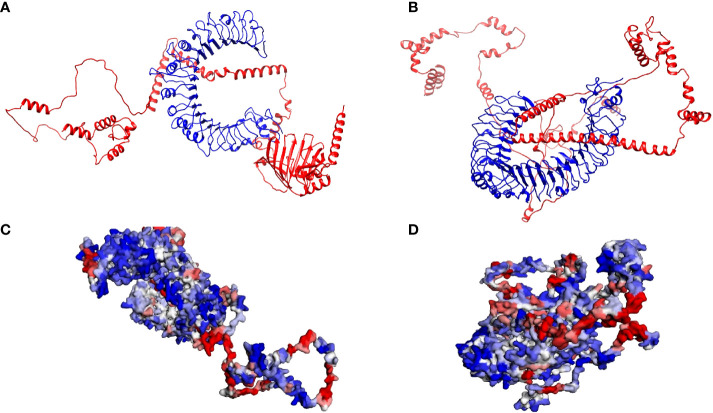
Analysis of the interactions between the respective Toll-Like Receptors and multi-epitope vaccine constructs **(A)** The crystalline structure of the first multi-epitope vaccine construct docked with TLR2. The multi-epitope chain is shown in red and the TLR2 in blue. **(B)** The crystalline structure of the second multi-epitope vaccine construct docked with TLR4. The multi-epitope chain is shown in red and the TLR4 in blue. **(C)** The solubility and aggregation propensity model of the first docked complex. The graphical representation model shows the soluble residues in red, the aggregation-prone residues in blue, and residues with no predicted influence shown in white. **(D)** The solubility and aggregation propensity model of the second docked complex. The graphical representation model shows the soluble residues in red, the aggregation-prone residues in blue, and residues with no predicted influence shown in white.

The best model energies for the docked vaccine complexes one and two were -186.17 kCal/mol and -163.86 kCal/mol, respectively (**Figures 5A, B**). There were 9 hydrogen bonds observed, 6 salt bridges and 135 non-bonded contacts observed between the first vaccine construct and TLR2 (**Supplementary Figure 8A**). The second complex contained 10 hydrogen bonds, 2 salt bridges and 124 non-bonded contacts (**Supplementary Figure 8B**). The generated contact maps for constructs one and two revealed various contacts among the different residues of the retrieved structures (**Supplementary Figures 9A, B**). The binding domains of the first construct to TLR2 and the original ligand i.e., *Streptococcus Pneumoniae* lipoteichoic acid, to TLR2 were mapped (**Supplementary Figures 10A, B**). **Supplementary Figures 11A, B** show the mapped binding domains of the second vaccine construct to TLR4 and the original MD-2 and LPS ligands to TLR4. These confirmed that the vaccine constructs bound well in the binding domains of the respective TLRs. The first construct’s minimal and maximal aggression scores were -4.05 and 3.74, respectively. The average score was -0.57, and the total score was -628.21. It was estimated that 204 residues from the complex were aggregation-prone, 634 were soluble, and 258 had negligible influence (**Figure 5C**). The TLR2 chain consisted of 39 aggregation-prone residues, 313 soluble resides and 198 residues of negligible influence. The MEV chain consisted of 165 aggregation-prone residues, 321 soluble residues and 60 negligible residues. The minimal and maximal aggression scores of the second complex were -3.95 and 3.57, respectively. The average and total scores were -0.52 and -586.12, respectively. The second complex consisted of 221 aggregation-prone residues, 645 soluble residues, and 259 residues with negligible influence (**Figure 5D**). The TLR4 chain contained 29 aggregation-prone residues, 338 soluble resides and 234 residues of negligible influence. The MEV chain consisted of 192 aggregation-prone residues, 307 soluble residues and 25 negligible residues.

#### 3.2.5 Immune simulations

**Figure 6:**
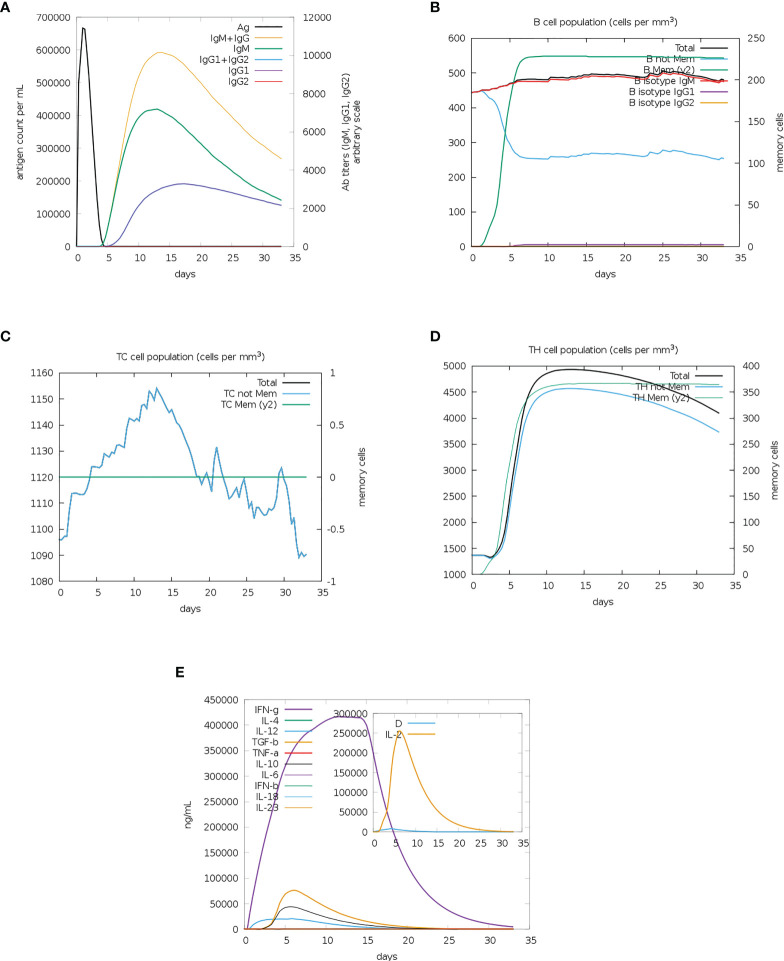
The immune simulation results from C-IMMSIM of MEV construct one. **(A)** The induced antigen and immunoglobin responses. **(B)** The B-cell population: total count, memory cells, and sub-divided into isotypes IgM, IgG1 and IgG2. **(C)** The CD8^+^ cytotoxic lymphocytes count. **(D)** The CD4^+^ T-helper lymphocyte count. **(E)** The induced cytokine response. The inset plot shows the danger signal together with IL-2.

A single dose of the MEV vaccine constructs elicited an observable immune response involving T- and B-cells. A sharp increase followed by a gradual decrease in antigen count was observed between 0 to 5 days for both vaccine constructs (**Figure 6A** and **Supplementary Figure 12A**). Immunoglobulin M (IgM) and IgM combined with IgG gradually increased between days 10 to 15 before decreasing, with the IgM and IgG combined levels displaying a greater increase than IgM alone (**Figure 6A** and **Supplementary Figure 12A**). There was a gradual increase in IgG1 from days 5 to 15 for both constructs, following which it was observed to decrease (**Figure 6A** and **Supplementary Figure 12A**). The IgG2 levels remained at 0 for the entire run for both constructs (**Figure 6A** and **Supplementary Figure 12A**). Vaccine construct one appears to have elicited a greater immunoglobin response than vaccine construct two (**Figure 6A** and **Supplementary Figure 12A**). The memory B-cells showed a sharp increase from day 0 to 5, followed by the maintenance of levels of B-cell memory for both constructs (**Figure 6B** and **Supplementary Figure 12B**). The population of non-memory B-cells showed a simultaneous decrease in both cases (**Figure 6B** and **Supplementary Figure 12B**). However, there were greater numbers of non-memory B-cells from the start of the simulation for construct one (**Figure 6B**). The B isotype IgM of both simulations remained relatively level, with minor increases in both cases (**Figure 6B** and **Supplementary Figure 12B**). B isotype IgG1 levels remained at 0 for the duration of the simulation for both constructs (**Figure 6B** and **Supplementary Figure 12B**). This differed from the B isotype IgG2, which had a minor increase for vaccine construct one and remained at 0 for vaccine construct two (**Figure 6B** and **Supplementary Figure 12B**). The CD8^+^ memory response remained constant for the duration of the simulation, while the non-memory cells displayed sharp increases and decreases (**Figure 6C** and **Supplementary Figure 12C**). The CD4^+^ T-cell responses for both constructs showed a gradual increase before 5 days, followed by a gradual decrease (**Figure 6D** and **Supplementary Figure 12D**). The count of CD4^+^ memory cells plateaued; however, the first construct elicited a greater memory response (**Figure 6D** and **Supplementary Figure 12D**). Both constructs induced the significant release of IFN-γ, with lower levels of IL-10, IL-12 and Transforming growth factor β (TGF-β) (**Figure 6E** and **Supplementary Figure 12E**). Tumour Necrosis Factor α (TNF-α) remained at 0 for both complexes for the duration of the simulation (**Figure 6E** and **Supplementary Figure 12E**). The inset plots show significant levels of IL-2 and the danger signal, with construct one eliciting a greater response in IL-2 (**Figure 6E** and **Supplementary Figure 12E**).

#### 3.2.6 Molecular dynamics simulations and post-molecular dynamics simulations analysis

**Figure 7:**
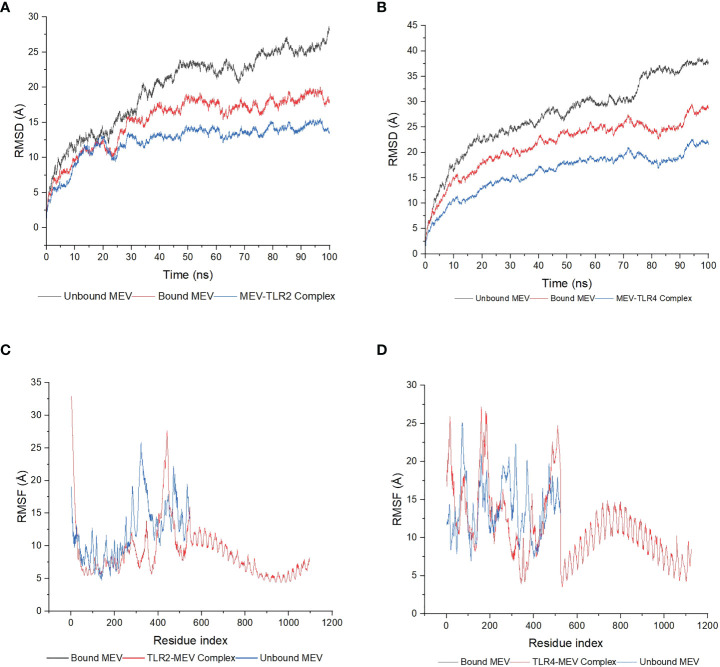
The molecular dynamics simulations of the MEV-TLR docked complexes, the MEV bound to the TLRs and the unbound MEVs. **(A)** The RMSD plot was generated based on construct one. The bound MEV is shown in red, the unbound MEV in grey and the TLR2-MEV complex in blue. **(B)** The RMSD plot was generated based on construct two. The bound MEV is shown in red, the unbound MEV in grey and the TLR4-MEV complex in blue. **(C)** The RMSF plot was generated based on construct one. The bound MEV is shown in grey, the unbound MEV in blue and the TLR2-MEV complex in red. **(D)** The RMSF plot based on construct two. The bound MEV is shown in grey, the unbound MEV in blue and the TLR4-MEV complex in red.

Both complexes and bound MEVs had lower RMSD and RMSF values than the unbound MEV (**Figure 7**). The fluctuation for the backbone atoms of the unbound MEV of construct 1 ranged from 0.56Å to 28.71Å, whereas the fluctuation for the backbone atoms of the bound MEV was within 0.56Å to 20.09Å (**Figure 7A**). The fluctuation of the backbone atoms of the unbound and bound MEV chains for construct two was within the range of 0.55Å to 38.72Å and 0.54Å to 29.61Å, respectively (**Figure 7B**). This indicated better stability for complexes one and two. The lower fluctuation of residues of the bound MEVs and complexes compared to the unbound MEVs indicates the enhanced stability of the vaccine in both complexes (**Figures 7C, D**). To gain further insight into the interaction between the MEVs and their respective TLRs in the complex, MM/GBSA was performed to determine the binding affinities (**Table 2**). The endpoint binding energies of both complexes indicate good interactions, with the MEV-TLR4 complex displaying a better degree of interactions than the MEV-TLR2 complex (**Table 2**). The negative Van der Waals energies for both complexes are favorable. The electrostatic, gas-phase and solvation-free energy contributions are shown to be significant.

**Table 2:** The energy composition profile (kCal/mol) based on the MM/GBSA analysis of the two vaccine complexes, consisting of the average and standard error of mean.

Energy component	MEV-TLR2 complex	MEV-TLR4 complex
*ΔE*_*VDW*_	-228.13 ± 0.64	-348.80 ± 1.14
*ΔE*_*ELE*_	-2225.03 ± 5.09	-3410.93 ± 7.54
*ΔG*_*gas*_	-2453.16 ± 5.54	-3759.72 ± 8.59
*ΔG*_*solv*_	2282.30 ± 5.15	3551.60 ± 7.76
*ΔG*_*bind*_	-170.86 ± 0.56	-208.13 ± 0.92

ΔE_VDW_ = The van der Waals contribution from molecular mechanics (MM). Δ_EELE_ =The electrostatic energy calculated by MM force field. ΔG_gas_ = The gas-phase energy contribution. ΔG_solv_ = The solvation-free energy contribution. ΔG_bind_ = The endpoint binding energy of the interaction of the complex.

**Figure 8:**
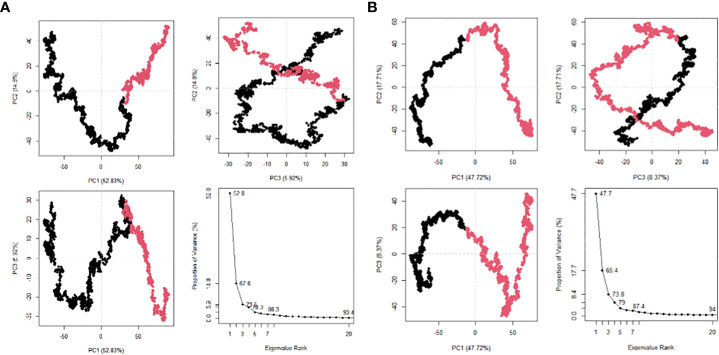
Post MDS analysis of the MEV constructs. **(A)** The PCA plots for MEV one in eigenvalue rank; PC2 vs PC1, PC2 vs PC3, PC3 vs PC1. The colours are based on order of time and the cumulative variability at each data point. **(B)** The PCA plots for MEV two in eigenvalue rank; PC2 vs PC1, PC2 vs PC3, PC3 vs PC1. The colours are based on order of time and the cumulative variability at each data point.

**Figure 9:**
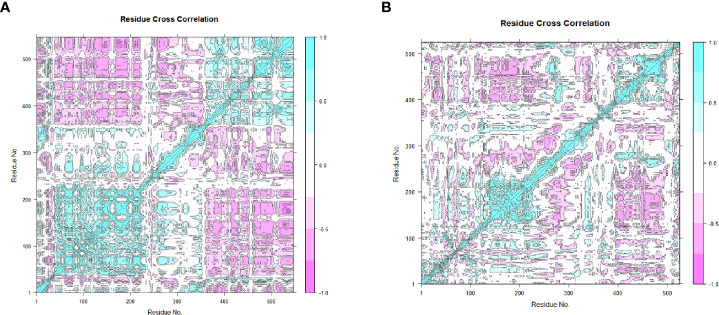
The dynamical cross-correlation maps generated based on the MEVs. The blue regions indicate the residues moving in a singular direction, while the pink regions indicate that the residues moved in opposite directions. **(A)** The dynamical cross-correlation map generated based on MEV one. **(B)** The dynamical cross-correlation map generated based on MEV two.

The 20 principal components captured 93.40% of the variance of the atom positional fluctuations of the first MEV construct during MDS (**Figure 8A**). The three PCs, i.e., PC1 to PC3, are accountable for 73.55% of the total proportion of variance shown in the eigenvalue plot (**Figure 8A**). PC1 contributed the largest variability, followed by PC2 and PC3, with proportions of 52.83%, 14.80% and 5.92%, respectively (**Figure 8A**). This differed from the variance of the MEV-TLR2 complex, wherein the 20 principal components captured 90.30% of the variance (**Supplementary Figure 13A**). PC1 was accountable for 39.62%, PC2 for 15.80%, and PC3 for 9.53% (**Supplementary Figure 13A**). This amounted to 64.95% of the total proportion of the variance shown in the eigenvalue plot (**Supplementary Figure 13A**). The 20 principal components of the second vaccine construct captured 94% of the variance of the atom positional fluctuations during MDS (**Figure 8B**). The three PCs, i.e., PC1 to PC3, are accountable for 73.80% of the total proportion of variance shown in the eigenvalue plot (**Figure 8B**). PC1 contributed the largest variability, followed by PC2 and PC3, with proportions of 47.72%, 17.71% and 8.37%, respectively (**Figure 8B**). This differed from the variance of the complex, wherein the 20 principal components captured 92% of the variance (**Supplementary Figure 14A**). The total proportion of the variance shown in the eigenvalue plot was 71.34% (**Supplementary Figure 14A**). PC1 was accountable for 44.35%, PC2 for 18.48%, and PC3 for 8.51% (**Supplementary Figure 14A**). The fluctuations of the residual-wise loadings were lower in both the complexes than in the vaccine constructs alone (**Supplementary Figures 13B, 14B, 15**). The two complexes and constructs displayed a series of residues moving in the same direction near to the diagonal, indicating the inter-correlation of the residues (**Figures 9A, B** and **Supplementary Figures 13C, 14C**).

## 4 Discussion

BU is a neglected disease that remains erratic in several tropical and subtropical countries (1). It is not fatal but is associated with permanent degrees of disability and severe morbidity (113). Early diagnostic techniques and effective treatments may lessen the severity of symptoms, thereby limiting the chance of disability (114). It should be noted that several socioeconomic factors may limit patients’ ability to receive treatment (115). This includes transportation costs and the need for long hospital stays associated with a loss of earnings and work opportunities (115). Patients may also experience stigmatization (115). A BU vaccine may reduce not only the physical burden of the disease but also the economic burden placed on the patient, family, and community. The BCG vaccine and conventionally designed *M. ulcerans-*specific vaccines have displayed limited efficacy in animal models (16, 17).

The immunoinformatics approach includes mapping T-cell and B-cell epitopes that can be used for disease and host-pathogen interaction understanding and analyses, allergy prediction and vaccine design (116). These epitopes should have a high affinity for MHC I and II molecules (117). Through the binding of T-cell epitopes to MHC I and II molecules and B-cell stimulation, the cell-mediated and humoral immune responses can be induced, respectively (118). Various studies have been conducted using similar immunoinformatics protocols to develop MEVs against various human diseases. This includes and is not limited to *Chandipura vesiculovirus* (CHPV) (119), malaria (57), cutaneous leishmaniasis (120), tuberculosis (121) and SARS Coronavirus-2 (SARS-CoV-2) (122). Two *in-silico* studies have been carried out for BU (33, 34). Both studies consisted of the identification, analysis and molecular docking of T-cell and B-cell epitopes originating from *M. ulcerans* (33, 34). Nain et al. (34) performed further analysis and generated a MEV construct, which underwent TLR docking and MDS. The studies yielded epitopes and a vaccine that displayed several desirable properties. The immunoinformatics protocol remains similar regardless of the target organism and disease, with the identification of T-cell and B-cell epitopes remaining a key step.

The source of the T-cell and B-cell epitopes are MFS transporter proteins. The MFS is the largest and most diverse superfamily of transmembrane secondary carriers (123). They serve as uniporters, symporters or antiporters across all domains of life (124). Their role in bacteria is mainly nutrient uptake and deleterious compound extrusion (125). MFS transporters have been implicated in a wide range of diseases and are potential drug targets (125). Based on their phylogeny and function, the Transporter Classification Database (TCDB) (https://tcdb.org) classified 16 families and 89 subfamilies within the MFS (126). Only seven protein crystal structures within six distinct MFS subfamilies have been identified presently, with proteins from each subfamily exhibiting low sequence similarity, distinct substrate specificities and different transport coupling mechanisms (35). However, the structural MFS fold is common among these structures (35). The epitopes isolated from the MFS transporter proteins and, by extension, the MEVs constructed using these epitopes displayed several desirable properties.

The immunogenic potential of the CD8^+^ epitopes and the cytokine-inducing capabilities for the CD4^+^ epitopes is promising. The TH type 1 (TH1) response is suggested to be critical in eradicating *M. ulcerans* infection (127). Previous studies using conventional *M. ulcerans* vaccines in animal models noted the induction of the IFN-γ, IL-2, and IL-10 (128–132). Cytokines associated with the TH1 response include IFN-γ, IL-2 and IL-12, while the TH2 response consists of IL-4, IL-5 and IL-13 (133). The potential of the CD4^+^ epitopes to induce IFN-γ is promising. High levels of IFN-γ have been observed in patients during the advanced phases of BU and once they have healed (127). The TH2 cytokines IL-4, IL-13 and anti-inflammatory cytokines IL-10 and TGF-β may play a detrimental role in the control of bacterial proliferation (134). The immune response data generated indicates that further analysis may be required regarding the generation of positive assays. The non-allergenic and non-toxic properties of the epitopes and subsequent MEV constructs indicate a degree of safety. Interestingly, the epitopes generated in this study were not found in the previous studies and displayed different properties from the epitopes generated in those studies (33, 34). The construction of the MEVs with the T-cell epitopes located in the N-terminus and the B-cell epitopes in the C-terminus was found to induce a greater affinity and specificity of antibodies in previous studies (135, 136). The selection of epitopes with low IC_50_ values was based on the implication that these epitopes would be high-affinity binders (137, 138).

The identification of different HLA alleles is a critical step for the prediction of T-cell epitopes (139). The highly polymorphic nature of MHC molecules results in varying frequencies of different HLA types in diverse ethnic groups (140). However, the ability of certain human populations to respond to a specific antigen may be limited if extreme levels of polymorphisms occur (140). T-cell epitopes should present the highest world population coverage possible (117). A vaccine with a broad range of reactivity for at least 90% of most ethnic populations may be acceptable for public health (141). Longmate et al. (141), found that this is achievable by using 11 uniquely defined HLA-restricted CD8^+^ epitopes. They suggested that the derivation of four or more CD8^+^ epitopes may provide 90% coverage for African or Asian ethnic groups (141). Compared to previous studies, the global population coverage of the BU-specific MEV was 99.55%, 56.36% and 99.80% for class I, class II and combined class, respectively (34). This study had greater global population coverages for class II and class combined; however, the class I value was lower. The range of selected regions was narrower in this study compared to the previous study (34). There was no population coverage generated for Ghana and a low population coverage of Côte d’Ivoire; however, the high population coverage generated for West Africa (99.95%) indicates that the prevalence of the T-cells epitopes and the restricted respective HLA alleles for the individual countries may not be high. Benin was not available for selection under the Population Coverage website. This must be addressed as Nigeria, Côte d’Ivoire, and Benin have reported high case numbers in 2021 (142).

Adjuvants were combined with the T-cell and B-cell epitopes to potentially induce an improved immune response without significant safety risk (143). However, possible adverse effects should still be taken into consideration (143). TLR agonists have been termed attractive candidates for human vaccines (144). LprG is a known TLR2 ligand (145). It was also selected as an adjuvant based on findings that indicated it triggered signals that lead to T-cell activation and the induction of effector functions when combined with T-cell receptor triggering (145). RpfE was selected as the corresponding adjuvant to TLR4 due to its role as an agonist (67). Choi et al. (146) found that RpfE has the potential to enhance dendritic cell (DC)-mediated T-cell activation. TLR4 and TLR2 were chosen for the MEV complexes due to their active participation in the innate immune response to *M. ulcerans* (147). TLR2 was found to be an important contributor to the innate immune recognition of *M. tuberculosis* (68). The production of *M. ulcerans*-mediated chemokines such as CXC chemokine ligand 8 (CXCL8) and CC chemokine ligand (CCL) 2 was found to be dependent on mainly TLR2 and, to an extent, TLR4 in primary human keratinocytes (147).

The addition of the linkers may prove advantageous in terms of the improvement in biological activity and increase in the expression yield (148). The GPGPG linker was chosen as it was found to eliminate junctional epitopes, which could alter the immune response towards insignificant or immunodominant epitopes (149). It was also found to have the ability to optimize the immunogenic capability of CD4^+^ epitopes (149). The AAY linkers also prevent the formation of junctional epitopes (150). However, the induction of significant changes to protein characteristics, such as hydrophilicity, flexibility, and α, β, turn, and coil regions, was observed (150). This can impact the stability of the protein and possibly reduce immunogenicity (150). The rigid EAAAK linker is able to limit the interactions between the sections of the MEVs (148). The separation of the MEV sections ensures that they can each function independently (148). It was found that the KK linker has the potential to overcome the generation of unexpected immune responses (151). However, it was observed that when peptides were located on the N-terminal side of the linker, the antibody induction was weak compared to the greater induction of antibodies when the peptide was on the C-terminal side (151). The KK linker consists of basic amino acids, i.e., lysine, and may increase the pI of the structure (152).

The optimal range for the CAI and GC content is 0.8-1.0 and 30-70%, respectively (153). This indicates the improved expression of the gene in the selected organism without translation errors (103). Both vaccine constructs were within the optimal ranges, indicating improved expression of both the sequences. It is reported that the expression of mammalian proteins in *E. coli* was dramatically increased, with increases between five- to fifteen-fold and a yield of up to 5% of the *E. coli* soluble protein (154). The successful *in-silico* cloning of the DNA sequences for both vaccine constructs into the high expression plasmid indicates promise for the ease and accuracy of the vaccine production (103).

The vaccine constructs displayed several desirable physicochemical properties, i.e., thermostability and stability. However, vaccine construct one may be the more attractive choice, as only it was identified as stable. The thermostability coupled with the melting temperature indicates the suitability of the constructs in endemic regions. The estimated half-life of both the constructs in mammalian cells suggests that the peptides might remain viable for a long enough period to potentially induce an effective immune response (155). The molecular weight for an ideal vaccine construct is estimated to be greater than approximately 40 to 50 kDa, as this will result in an increase in the uptake of the construct by the lymphatic system (156). The molecular weight of both constructs is greater than 50kDa, indicating a potential hindering of the lymphatic system uptake. The basic nature of the constructs may hinder further development of the vaccine, as the preferred pH of vaccines should be closer to the natural pH of fluids of the human body (152). The hydrophilic nature of the constructs indicates the potential of these constructs to interact with water molecules (157). This may be beneficial, as water has been observed to act as a stabilizing factor between two hydrophilic residues over far distances (158). The presence of these structures has been observed to improve alpha-helical proteins, and water is also thought to participate in loop stabilization (158).

Alpha-helices have been noted to return to their native structures during testing of synthetic peptides, resulting in their recognition by naturally induced antibodies during infection (153). Isolated extended strands are observed to commonly occur in proteins (159). The refinement of both constructs resulted in the improvement of the structures. The majority of residues in both constructs were in favored regions per the Ramachandran plots. The allowed regions displays which values of the Phi (φ)/Psi (ψ) angles are possible for an amino acid, X, in a ala-X-ala tripeptide (160). The observation of this distribution of values can be used for structural validation of protein structures (160). A larger allowable area within the four quadrants may occur as a result of residues with less bulky or no side chains, which can have a higher number of possible combinations of φ and ψ (161). This differs from residues with bulky side chains, which may have a lower number of φ and ψ combinations, resulting in a smaller allowable area (161). The negative Z-scores indicate the accuracy of the predicted constructs (162). The good quality of the refined 3D structures is suggested by the predicted ERRAT scores greater than 50 (162). It also indicates the potential of these structures to serve as reliable models for further analysis (162). Protein interactions such as hydrogen bonds and salt bridges are important in protein binding due to their role in stabilizing the complex (163). The presence of these interactions in the complexes further indicates the stability of the structure. The preservation of protein function and the limitation of aggregation relies on the balancing of protein stability and solubility (164). The negative average and total aggregation scores indicate high normalized and global solubility, respectively (97). Both complexes consisted of more soluble residues than aggregation-prone residues. This is favorable as the formation of aggregates may result in reduced production yields and unpredictable immune responses (165).

IgG is critical for immune memory and a maintained immune response (166). Varying levels of different isotypes of specific IgG antibodies have been observed in mice models immunized with *M. ulcerans* specific vaccines (131, 167–170). The release of combined IgG and IgM for both constructs is promising, with the first construct eliciting a greater response. IgM is observed in the early stages of the antibody response following the introduction of antigens (171). This was observed in this study as levels of B isotype IgM were maintained through the simulation. However, it should be noted that the simulation for the vaccine was run with a single dose, and generally, vaccines require multiple doses to ensure long-term protection (57).

Molecular docking is used to predict the manner in which different formations or combinations of molecules may connect to a suitable target site (139). In this study, it was utilized to study the binding interactions between the T-cell epitopes and their respective alleles and the vaccine constructs with their respective TLRs. The low binding energies indicate the stability of the interaction. MDS was carried out to analyze the binding interactions of the vaccines and the receptors (103). The examination of these results supplies information regarding the dynamics and binding states of the receptor to the vaccines (172). The stability and appropriateness of the vaccine and receptor binding is linked to variation in the RMSD values (173). The lower fluctuation values of the bound vaccines and vaccine complexes compared to the unbound vaccines indicate increased stability once the binding was carried out. This pattern was also observed with the RMSF values, more clearly with the first complex, thereby solidifying the complex’s stability and indicating the complex chains’ flexibility (57). The negative binding, Van der Waals and electrostatic energy values indicate a high binding affinity between protein-protein or protein-ligand interactions (174). PCA provides information regarding the structural and energy data generated from MDS on the complexes and individual MEVs (175). The shifting of color from black to pink within the PC plots is indicative of periodic jumps during MDS (112). The vaccine constructs contained both negatively- and positively correlated residue motions. The addition of the TLRs exhibited a significant impact in PCA and DCCM.

The design of BU vaccines is ongoing, and reverse vaccinology opens up a promising venture. The various analyses performed have indicated that the vaccine constructs display several favorable characteristics. The constructs were found to be antigenic, immunogenic, non-allergenic, non-toxic, and stable. The cytokine and further immune response simulations indicate the induction of several advantageous responses. The vaccine-TLR complexes have displayed strong and stable binding interactions. This study serves to provide additional *in-silico* candidates in BU vaccine design. It is important to remember that this study was conducted *in-silico*, and the results are within the boundaries of the tools used. Laboratory validation is required to analyze these constructs’ suitability and safety in model organisms. This study further shows the potential for a vaccine against *M. ulcerans*.

## Data availability statement

Publicly available datasets were analyzed in this study. This data can be found here: National Center for Biotechnology Information (NCBI) with the following accession numbers: GCA_022374915.1 - https://www.ncbi.nlm.nih.gov/assembly/GCA_022374915.1, GCA_020150655.1 - https://www.ncbi.nlm.nih.gov/assembly/GCA_020150655.1, GCA_020616615.1 - https://www.ncbi.nlm.nih.gov/assembly/GCA_020616615.1, GCA_900638745.1 - https://www.ncbi.nlm.nih.gov/assembly/GCA_900638745.1, GCA_001870585.1 - https://www.ncbi.nlm.nih.gov/assembly/GCA_001870585.1, GCA_901411635.1 - https://www.ncbi.nlm.nih.gov/assembly/GCA_901411635.1, GCA_902506705.1 - https://www.ncbi.nlm.nih.gov/assembly/GCA_902506705.1, GCA_900683785.1 - https://www.ncbi.nlm.nih.gov/assembly/GCA_900683785.1, GCA_000013925.2 - https://www.ncbi.nlm.nih.gov/assembly/GCA_000013925.2, GCA_000524035.1 - https://www.ncbi.nlm.nih.gov/assembly/GCA_000524035.1, GCA_002355775.1 - https://www.ncbi.nlm.nih.gov/assembly/GCA_002355775.1, GCA_002356495.1 - https://www.ncbi.nlm.nih.gov/assembly/GCA_002356495.1.

## Author contributions

TI, MA, and AA conceived the idea. TI conducted the *in-silico* work on the vaccine with assistance from VA and LM. TI wrote the manuscript while LM, VA, AA, MA and MO edited the manuscript. MA and MO supervised the work. All authors contributed to the article and approved the submitted version.

## Funding

This work is based on the research supported wholly by the National Research Foundation of South Africa (Grant Number 141385), as a bursary award for the first author.

## Acknowledgments

The authors wish to acknowledge the Centre for High-Performance Computing, South Africa, for the computer programs and facilities used for this project.

## Conflict of interest

The authors declare that the research was conducted in the absence of any commercial or financial relationships that could be construed as a potential conflict of interest.

## Publisher’s note

All claims expressed in this article are solely those of the authors and do not necessarily represent those of their affiliated organizations, or those of the publisher, the editors and the reviewers. Any product that may be evaluated in this article, or claim that may be made by its manufacturer, is not guaranteed or endorsed by the publisher.
