# Supplementary material for: Identification of potential candidate vaccines against Mycobacterium ulcerans based on the major facilitator superfamily transporter protein
Source: Front Immunol. 2022 Nov 8;13:1023558. doi: 10.3389/fimmu.2022.1023558 (PMC9679648; doi:10.3389/fimmu.2022.1023558)
Supplement: Supplementary file 1 [file DataSheet_1.docx]

Supplementary materials

**Supplementary Table 1**. The genome assemblies used in the analysis, accession numbers, species name, strain of origin and level of organization

| Accession number | Species name | Strain | Level of organization |
| --- | --- | --- | --- |
| GCA_022374915.1 | *Mycobacterium ulcerans* | ATCC 19423 | Complete genome |
| GCA_020150655.1 | *Mycobacterium ulcerans* | BS123 | Complete genome |
| GCA_020616615.1 | *Mycobacterium ulcerans* | JKD8049 | Complete genome |
| GCA_900638745.1 | *Mycobacterium ulcerans* | SGL03 | Complete genome |
| GCA_001870585.1 | *Mycobacterium ulcerans* | S4018 | Scaffold |
| GCA_901411635.1 | *Mycobacterium ulcerans* | P7741 | Contig |
| GCA_902506705.1 | *Mycobacterium ulcerans* | CSURQ0185 | Scaffold |
| GCA_900683785.1 | *Mycobacterium ulcerans* | P7741 | Contig |
| GCA_000013925.2 | *Mycobacterium ulcerans Agy99* | Agy99 | Complete genome |
| GCA_000524035.1 | *Mycobacterium ulcerans str. Harvey* | Harvey | Contig |
| GCA_002355775.1 | *Mycobacterium ulcerans subsp. shinshuense* | ATCC 33728 | Complete genome |
| GCA_002356495.1 | *Mycobacterium ulcerans subsp. shinshuense* | ATCC 33728 | Chromosome |

**Supplementary Table 2.** The top ten highest HLA-alleles and frequencies for the identified endemic countries generated from the Allele Frequency Net Database

|  | | Country | | | | | | | | | | | | | | | | | | | | | | | | | | | | | | | | | | | | | | | | | | | | | | | | |
| --- | --- | --- | --- | --- | --- | --- | --- | --- | --- | --- | --- | --- | --- | --- | --- | --- | --- | --- | --- | --- | --- | --- | --- | --- | --- | --- | --- | --- | --- | --- | --- | --- | --- | --- | --- | --- | --- | --- | --- | --- | --- | --- | --- | --- | --- | --- | --- | --- | --- | --- |
|  | | Australia | | | | | | | | | | | | | | | | | | | | | | | | | | | | | | | | | | | | | | | | | | | | | | | | |
|  | | | Australia Cape York Peninsula Aborigine (n=103) | | | | | | | | | | | | | | Australia Groote Eylandt Aborigine (n=75) | | | | | | | | | | | | Australia New South Wales Caucasian (n=134) | | | | | | | | | | | Australia Yuendumu Aborigine (n=191) | | | | | | | | | | |
| HLA Allele | | | Allele Freq | | | | | | | | | | | | | | Allele Freq | | | | | | | | | | | | Allele Freq | | | | | | | | | | | Allele Freq | | | | | | | | | | |
| A*01:01 | | | 0.053 | | | | | | | | | | | | | | 0.027 | | | | | | | | | | | | 0.187 | | | | | | | | | | | 0.008 | | | | | | | | | | |
| A*02:01 | | | 0.175 | | | | | | | | | | | | | | 0.107 | | | | | | | | | | | | 0.261 | | | | | | | | | | | 0.113 | | | | | | | | | | |
| A*02:05 | | |  | | | | | | | | | | | | | |  | | | | | | | | | | | | 0.008 | | | | | | | | | | |  | | | | | | | | | | |
| A*02:07 | | |  | | | | | | | | | | | | | |  | | | | | | | | | | | | 0.008 | | | | | | | | | | |  | | | | | | | | | | |
| A*03:01 | | | 0.044 | | | | | | | | | | | | | |  | | | | | | | | | | | | 0.138 | | | | | | | | | | | 0.003 | | | | | | | | | | |
| A*11:01 | | | 0.180 | | | | | | | | | | | | | | 0.240 | | | | | | | | | | | | 0.067 | | | | | | | | | | | 0.076 | | | | | | | | | | |
| A*23:01 | | |  | | | | | | | | | | | | | |  | | | | | | | | | | | | 0.019 | | | | | | | | | | |  | | | | | | | | | | |
| A*24:02 | | | 0.223 | | | | | | | | | | | | | | 0.293 | | | | | | | | | | | | 0.082 | | | | | | | | | | | 0.298 | | | | | | | | | | |
| A*24:06 | | |  | | | | | | | | | | | | | |  | | | | | | | | | | | |  | | | | | | | | | | | 0.013 | | | | | | | | | | |
| A*24:13 | | |  | | | | | | | | | | | | | |  | | | | | | | | | | | |  | | | | | | | | | | | 0.018 | | | | | | | | | | |
|  | | |  | | | | | | | | | | | | | |  | | | | | | | | | | | |  | | | | | | | | | | |  | | | | | | | | | | |
| B*07:02 | | | 0.045 | | | | | | | | | | | | | |  | | | | | | | | | | | | 0.120 | | | | | | | | | | |  | | | | | | | | | | |
| B*07:05 | | |  | | | | | | | | | | | | | |  | | | | | | | | | | | | 0.004 | | | | | | | | | | |  | | | | | | | | | | |
| B*08:01 | | | 0.035 | | | | | | | | | | | | | | 0.020 | | | | | | | | | | | | 0.173 | | | | | | | | | | |  | | | | | | | | | | |
| B*13:01 | | | 0.270 | | | | | | | | | | | | | | 0.233 | | | | | | | | | | | |  | | | | | | | | | | | 0.244 | | | | | | | | | | |
| B*13:02 | | |  | | | | | | | | | | | | | |  | | | | | | | | | | | | 0.008 | | | | | | | | | | |  | | | | | | | | | | |
| B*14:01 | | | 0.005 | | | | | | | | | | | | | | 0.007 | | | | | | | | | | | | 0.011 | | | | | | | | | | |  | | | | | | | | | | |
| B*14:02 | | |  | | | | | | | | | | | | | |  | | | | | | | | | | | | 0.011 | | | | | | | | | | |  | | | | | | | | | | |
| B*15:01 | | |  | | | | | | | | | | | | | |  | | | | | | | | | | | | 0.056 | | | | | | | | | | | 0.008 | | | | | | | | | | |
| B*15:02 | | |  | | | | | | | | | | | | | | 0.007 | | | | | | | | | | | |  | | | | | | | | | | |  | | | | | | | | | | |
| B*15:03 | | |  | | | | | | | | | | | | | |  | | | | | | | | | | | | 0.004 | | | | | | | | | | |  | | | | | | | | | | |
|  | | |  | | | | | | | | | | | | | |  | | | | | | | | | | | |  | | | | | | | | | | |  | | | | | | | | | | |
| C*01:02 | | | 0.185 | | | | | | | | | | | | | | 0.267 | | | | | | | | | | | |  | | | | | | | | | | | 0.247 | | | | | | | | | | |
| C*02:02 | | | 0.006 | | | | | | | | | | | | | |  | | | | | | | | | | | |  | | | | | | | | | | |  | | | | | | | | | | |
| C*03:02 | | | 0.006 | | | | | | | | | | | | | |  | | | | | | | | | | | |  | | | | | | | | | | |  | | | | | | | | | | |
| C*03:03 | | | 0.028 | | | | | | | | | | | | | | 0.151 | | | | | | | | | | | |  | | | | | | | | | | | 0.055 | | | | | | | | | | |
| C*03:04 | | | 0.022 | | | | | | | | | | | | | | 0.007 | | | | | | | | | | | |  | | | | | | | | | | | 0.005 | | | | | | | | | | |
| C*04:01 | | | 0.275 | | | | | | | | | | | | | | 0.253 | | | | | | | | | | | |  | | | | | | | | | | | 0.268 | | | | | | | | | | |
| C*04:03 | | | 0.157 | | | | | | | | | | | | | | 0.123 | | | | | | | | | | | |  | | | | | | | | | | | 0.151 | | | | | | | | | | |
| C*05:01 | | | 0.034 | | | | | | | | | | | | | | 0.014 | | | | | | | | | | | |  | | | | | | | | | | | 0.003 | | | | | | | | | | |
| C*06:02 | | | 0.028 | | | | | | | | | | | | | | 0.014 | | | | | | | | | | | |  | | | | | | | | | | |  | | | | | | | | | | |
| C*07:01 | | | 0.034 | | | | | | | | | | | | | | 0.021 | | | | | | | | | | | |  | | | | | | | | | | |  | | | | | | | | | | |
|  | | |  | | | | | | | | | | | | | |  | | | | | | | | | | | |  | | | | | | | | | | |  | | | | | | | | | | |
| DPB1*01:01 | | | 0.010 | | | | | | | | | | | | | |  | | | | | | | | | | | |  | | | | | | | | | | |  | | | | | | | | | | |
| DPB1*02:01 | | | 0.156 | | | | | | | | | | | | | |  | | | | | | | | | | | |  | | | | | | | | | | |  | | | | | | | | | | |
| DPB1*03:01 | | | 0.031 | | | | | | | | | | | | | |  | | | | | | | | | | | |  | | | | | | | | | | |  | | | | | | | | | | |
| DPB1*04:01 | | | 0.104 | | | | | | | | | | | | | |  | | | | | | | | | | | |  | | | | | | | | | | |  | | | | | | | | | | |
| DPB1*05:01 | | | 0.453 | | | | | | | | | | | | | |  | | | | | | | | | | | |  | | | | | | | | | | |  | | | | | | | | | | |
| DPB1*11:01 | | | 0.010 | | | | | | | | | | | | | |  | | | | | | | | | | | |  | | | | | | | | | | |  | | | | | | | | | | |
| DPB1*13:01 | | | 0.010 | | | | | | | | | | | | | |  | | | | | | | | | | | |  | | | | | | | | | | |  | | | | | | | | | | |
| DPB1*15:01 | | | 0.016 | | | | | | | | | | | | | |  | | | | | | | | | | | |  | | | | | | | | | | |  | | | | | | | | | | |
| DPB1*16:01 | | | 0.010 | | | | | | | | | | | | | |  | | | | | | | | | | | |  | | | | | | | | | | |  | | | | | | | | | | |
| DPB1*22:01 | | | 0.198 | | | | | | | | | | | | | |  | | | | | | | | | | | |  | | | | | | | | | | |  | | | | | | | | | | |
|  | | |  | | | | | | | | | | | | | |  | | | | | | | | | | | |  | | | | | | | | | | |  | | | | | | | | | | |
| DQA1*01:01 | | | 0.167 | | | | | | | | | | | | | |  | | | | | | | | | | | |  | | | | | | | | | | |  | | | | | | | | | | |
| DQA1*01:02 | | | 0.096 | | | | | | | | | | | | | |  | | | | | | | | | | | |  | | | | | | | | | | |  | | | | | | | | | | |
| DQA1*01:03 | | | 0.359 | | | | | | | | | | | | | |  | | | | | | | | | | | |  | | | | | | | | | | |  | | | | | | | | | | |
| DQA1*02:01 | | | 0.040 | | | | | | | | | | | | | |  | | | | | | | | | | | |  | | | | | | | | | | |  | | | | | | | | | | |
| DQA1*03:01:01 | | | 0.202 | | | | | | | | | | | | | |  | | | | | | | | | | | |  | | | | | | | | | | |  | | | | | | | | | | |
| DQA1*05:01 | | | 0.131 | | | | | | | | | | | | | |  | | | | | | | | | | | |  | | | | | | | | | | |  | | | | | | | | | | |
| DQA1*06:01 | | | 0.005 | | | | | | | | | | | | | |  | | | | | | | | | | | |  | | | | | | | | | | |  | | | | | | | | | | |
|  | | |  | | | | | | | | | | | | | |  | | | | | | | | | | | |  | | | | | | | | | | |  | | | | | | | | | | |
| DQB1*02:01 | | | 0.071 | | | | | | | | | | | | | |  | | | | | | | | | | | |  | | | | | | | | | | |  | | | | | | | | | | |
| DQB1*03:01 | | | 0.091 | | | | | | | | | | | | | |  | | | | | | | | | | | |  | | | | | | | | | | |  | | | | | | | | | | |
| DQB1*03:02 | | | 0.045 | | | | | | | | | | | | | |  | | | | | | | | | | | |  | | | | | | | | | | |  | | | | | | | | | | |
| DQB1*03:03 | | | 0.015 | | | | | | | | | | | | | |  | | | | | | | | | | | |  | | | | | | | | | | |  | | | | | | | | | | |
| DQB1*04:01 | | | 0.020 | | | | | | | | | | | | | |  | | | | | | | | | | | |  | | | | | | | | | | |  | | | | | | | | | | |
| DQB1*04:02 | | | 0.126 | | | | | | | | | | | | | |  | | | | | | | | | | | |  | | | | | | | | | | |  | | | | | | | | | | |
| DQB1*05:01 | | | 0.030 | | | | | | | | | | | | | |  | | | | | | | | | | | |  | | | | | | | | | | |  | | | | | | | | | | |
| DQB1*05:02 | | | 0.010 | | | | | | | | | | | | | |  | | | | | | | | | | | |  | | | | | | | | | | |  | | | | | | | | | | |
| DQB1*05:03 | | | 0.217 | | | | | | | | | | | | | |  | | | | | | | | | | | |  | | | | | | | | | | |  | | | | | | | | | | |
| DQB1*06:01 | | | 0.313 | | | | | | | | | | | | | |  | | | | | | | | | | | |  | | | | | | | | | | |  | | | | | | | | | | |
| DRB1*01:01 | | | 0.010 | | | | | | | | | | | | | |  | | | | | | | | | | | |  | | | | | | | | | | |  | | | | | | | | | | |
| DRB1*01:03 | | | 0.005 | | | | | | | | | | | | | |  | | | | | | | | | | | |  | | | | | | | | | | |  | | | | | | | | | | |
| DRB1*03:01 | | | 0.045 | | | | | | | | | | | | | |  | | | | | | | | | | | |  | | | | | | | | | | |  | | | | | | | | | | |
| DRB1*04:01 | | | 0.005 | | | | | | | | | | | | | |  | | | | | | | | | | | |  | | | | | | | | | | |  | | | | | | | | | | |
| DRB1*04:03 | | | 0.010 | | | | | | | | | | | | | |  | | | | | | | | | | | |  | | | | | | | | | | |  | | | | | | | | | | |
| DRB1*04:04 | | | 0.015 | | | | | | | | | | | | | |  | | | | | | | | | | | |  | | | | | | | | | | |  | | | | | | | | | | |
| DRB1*04:05 | | | 0.086 | | | | | | | | | | | | | |  | | | | | | | | | | | |  | | | | | | | | | | | 0.004 | | | | | | | | | | |
| DRB1*04:10 | | | 0.025 | | | | | | | | | | | | | |  | | | | | | | | | | | |  | | | | | | | | | | |  | | | | | | | | | | |
| DRB1*04:11 | | | 0.015 | | | | | | | | | | | | | |  | | | | | | | | | | | |  | | | | | | | | | | |  | | | | | | | | | | |
| DRB1*04:12 | | |  | | | | | | | | | | | | | |  | | | | | | | | | | | |  | | | | | | | | | | | 0.076 | | | | | | | | | | |
|  | | |  | | | | | | | | | | | | | |  | | | | | | | | | | | |  | | | | | | | | | | |  | | | | | | | | | | |
| Congo | | | | | | | | | | | | | | | | | | | | | | | | | | | | | | | | | | | | | | | | | | | | | | | | | | |
| Congo Kinshasa Bantu (n=90) | | | | | | | | | | | | | | | | | | | | | | | | | | | | | | | | | | | | | | | | | | | | | | | | | | |
| DPB1*01:01 | | | 0.229 | | | | | | | | | | | | | | | | | | | | | | | | | | | | | | | | | | | | | | | | | | | | | | | |
| DPB1*02:01 | | | 0.175 | | | | | | | | | | | | | | | | | | | | | | | | | | | | | | | | | | | | | | | | | | | | | | | |
| DPB1*03:01 | | | 0.060 | | | | | | | | | | | | | | | | | | | | | | | | | | | | | | | | | | | | | | | | | | | | | | | |
| DPB1*04:01 | | | 0.054 | | | | | | | | | | | | | | | | | | | | | | | | | | | | | | | | | | | | | | | | | | | | | | | |
| DPB1*04:02 | | | 0.199 | | | | | | | | | | | | | | | | | | | | | | | | | | | | | | | | | | | | | | | | | | | | | | | |
| DPB1*11:01 | | | 0.030 | | | | | | | | | | | | | | | | | | | | | | | | | | | | | | | | | | | | | | | | | | | | | | | |
| DPB1*13:01 | | | 0.048 | | | | | | | | | | | | | | | | | | | | | | | | | | | | | | | | | | | | | | | | | | | | | | | |
| DPB1*14:01 | | | 0.006 | | | | | | | | | | | | | | | | | | | | | | | | | | | | | | | | | | | | | | | | | | | | | | | |
| DPB1*17:01 | | | 0.042 | | | | | | | | | | | | | | | | | | | | | | | | | | | | | | | | | | | | | | | | | | | | | | | |
| DPB1*18:01 | | | 0.073 | | | | | | | | | | | | | | | | | | | | | | | | | | | | | | | | | | | | | | | | | | | | | | | |
|  | | |  | | | | | | | | | | | | | | | | | | | | | | | | | | | | | | | | | | | | | | | | | | | | | | | |
| DQA1*01:01 | | | 0.072 | | | | | | | | | | | | | | | | | | | | | | | | | | | | | | | | | | | | | | | | | | | | | | | |
| DQA1*01:02 | | | 0.417 | | | | | | | | | | | | | | | | | | | | | | | | | | | | | | | | | | | | | | | | | | | | | | | |
| DQA1*01:03 | | | 0.039 | | | | | | | | | | | | | | | | | | | | | | | | | | | | | | | | | | | | | | | | | | | | | | | |
| DQA1*01:04 | | | 0.006 | | | | | | | | | | | | | | | | | | | | | | | | | | | | | | | | | | | | | | | | | | | | | | | |
| DQA1*01:05 | | | 0.061 | | | | | | | | | | | | | | | | | | | | | | | | | | | | | | | | | | | | | | | | | | | | | | | |
| DQA1*02:01 | | | 0.067 | | | | | | | | | | | | | | | | | | | | | | | | | | | | | | | | | | | | | | | | | | | | | | | |
| DQA1*03:01 | | | 0.011 | | | | | | | | | | | | | | | | | | | | | | | | | | | | | | | | | | | | | | | | | | | | | | | |
| DQA1*03:02 | | | 0.011 | | | | | | | | | | | | | | | | | | | | | | | | | | | | | | | | | | | | | | | | | | | | | | | |
| DQA1*03:03 | | | 0.078 | | | | | | | | | | | | | | | | | | | | | | | | | | | | | | | | | | | | | | | | | | | | | | | |
| DQA1*04:01 | | | 0.056 | | | | | | | | | | | | | | | | | | | | | | | | | | | | | | | | | | | | | | | | | | | | | | | |
|  | | |  | | | | | | | | | | | | | | | | | | | | | | | | | | | | | | | | | | | | | | | | | | | | | | | |
| DQB1*02:01 | | | 0.166 | | | | | | | | | | | | | | | | | | | | | | | | | | | | | | | | | | | | | | | | | | | | | | | |
| DQB1*02:02 | | | 0.017 | | | | | | | | | | | | | | | | | | | | | | | | | | | | | | | | | | | | | | | | | | | | | | | |
| DQB1*03:01 | | | 0.155 | | | | | | | | | | | | | | | | | | | | | | | | | | | | | | | | | | | | | | | | | | | | | | | |
| DQB1*03:02 | | | 0.006 | | | | | | | | | | | | | | | | | | | | | | | | | | | | | | | | | | | | | | | | | | | | | | | |
| DQB1*03:03:02 | | | 0.017 | | | | | | | | | | | | | | | | | | | | | | | | | | | | | | | | | | | | | | | | | | | | | | | |
| DQB1*04:02 | | | 0.039 | | | | | | | | | | | | | | | | | | | | | | | | | | | | | | | | | | | | | | | | | | | | | | | |
| DQB1*05:01 | | | 0.194 | | | | | | | | | | | | | | | | | | | | | | | | | | | | | | | | | | | | | | | | | | | | | | | |
| DQB1*05:02 | | | 0.011 | | | | | | | | | | | | | | | | | | | | | | | | | | | | | | | | | | | | | | | | | | | | | | | |
| DQB1*05:03 | | | 0.011 | | | | | | | | | | | | | | | | | | | | | | | | | | | | | | | | | | | | | | | | | | | | | | | |
| DQB1*06:02 | | | 0.300 | | | | | | | | | | | | | | | | | | | | | | | | | | | | | | | | | | | | | | | | | | | | | | | |
| Liberia | | | | | | | | | | | | | | | | | | | | | | | | | | | | | | | | | | | | | | | | | | | | | | | | | | |
| Liberia Bong County (n=110) | | | | | | | | | | | | | | | | | | | | | | | | | | | | | | | | | | | | | | | | | | | | | | | | | | |
| DPA1*01:03 | | | 0.095 | | | | | | | | | | | | | | | | | | | | | | | | | | | | | | | | | | | | | | | | | | | | | | | |
| DPA1*02:01:01 | | | 0.436 | | | | | | | | | | | | | | | | | | | | | | | | | | | | | | | | | | | | | | | | | | | | | | | |
| DPA1*02:01:02 | | | 0.023 | | | | | | | | | | | | | | | | | | | | | | | | | | | | | | | | | | | | | | | | | | | | | | | |
| DPA1*02:02:01 | | | 0.050 | | | | | | | | | | | | | | | | | | | | | | | | | | | | | | | | | | | | | | | | | | | | | | | |
| DPA1*02:02:02 | | | 0.241 | | | | | | | | | | | | | | | | | | | | | | | | | | | | | | | | | | | | | | | | | | | | | | | |
| DPA1*03:01 | | | 0.155 | | | | | | | | | | | | | | | | | | | | | | | | | | | | | | | | | | | | | | | | | | | | | | | |
|  | | |  | | | | | | | | | | | | | | | | | | | | | | | | | | | | | | | | | | | | | | | | | | | | | | | |
| DPB1*01:01 | | | 0.559 | | | | | | | | | | | | | | | | | | | | | | | | | | | | | | | | | | | | | | | | | | | | | | | |
| DPB1*02:01 | | | 0.050 | | | | | | | | | | | | | | | | | | | | | | | | | | | | | | | | | | | | | | | | | | | | | | | |
| DPB1*03:01 | | | 0.023 | | | | | | | | | | | | | | | | | | | | | | | | | | | | | | | | | | | | | | | | | | | | | | | |
| DPB1*04:01 | | | 0.055 | | | | | | | | | | | | | | | | | | | | | | | | | | | | | | | | | | | | | | | | | | | | | | | |
| DPB1*04:02 | | | 0.095 | | | | | | | | | | | | | | | | | | | | | | | | | | | | | | | | | | | | | | | | | | | | | | | |
| DPB1*06:01 | | | 0.005 | | | | | | | | | | | | | | | | | | | | | | | | | | | | | | | | | | | | | | | | | | | | | | | |
| DPB1*11:01 | | | 0.009 | | | | | | | | | | | | | | | | | | | | | | | | | | | | | | | | | | | | | | | | | | | | | | | |
| DPB1*13:01 | | | 0.032 | | | | | | | | | | | | | | | | | | | | | | | | | | | | | | | | | | | | | | | | | | | | | | | |
| DPB1*15:01 | | | 0.005 | | | | | | | | | | | | | | | | | | | | | | | | | | | | | | | | | | | | | | | | | | | | | | | |
| DPB1*17:01 | | | 0.068 | | | | | | | | | | | | | | | | | | | | | | | | | | | | | | | | | | | | | | | | | | | | | | | |
|  | | |  | | | | | | | | | | | | | | | | | | | | | | | | | | | | | | | | | | | | | | | | | | | | | | | |
| Nigeria | | | | | | | | | | | | | | | | | | | | | | | | | | | | | | | | | | | | | | | | | | | | | | | | | | |
| Nigeria Southwest Abanla Yoruba (n=130) | | | | | | | | | | | | | | | | | | | | | | | | | | | | | | | | | | | | | | | | | | | | | | | | | | |
| DPA1*01:03 | | | 0.235 | | | | | | | | | | | | | | | | | | | | | | | | | | | | | | | | | | | | | | | | | | | | | | | |
| DPA1*02:01:01 | | | 0.323 | | | | | | | | | | | | | | | | | | | | | | | | | | | | | | | | | | | | | | | | | | | | | | | |
| DPA1*02:01:02 | | | 0.012 | | | | | | | | | | | | | | | | | | | | | | | | | | | | | | | | | | | | | | | | | | | | | | | |
| DPA1*02:02:01 | | | 0.012 | | | | | | | | | | | | | | | | | | | | | | | | | | | | | | | | | | | | | | | | | | | | | | | |
| DPA1*02:02:02 | | | 0.304 | | | | | | | | | | | | | | | | | | | | | | | | | | | | | | | | | | | | | | | | | | | | | | | |
| DPA1*03:01 | | | 0.115 | | | | | | | | | | | | | | | | | | | | | | | | | | | | | | | | | | | | | | | | | | | | | | | |
|  | | |  | | | | | | | | | | | | | | | | | | | | | | | | | | | | | | | | | | | | | | | | | | | | | | | |
| DPB1*01:01 | | | 0.531 | | | | | | | | | | | | | | | | | | | | | | | | | | | | | | | | | | | | | | | | | | | | | | | |
| DPB1*02:01 | | | 0.085 | | | | | | | | | | | | | | | | | | | | | | | | | | | | | | | | | | | | | | | | | | | | | | | |
| DPB1*03:01 | | | 0.019 | | | | | | | | | | | | | | | | | | | | | | | | | | | | | | | | | | | | | | | | | | | | | | | |
| DPB1*04:01 | | | 0.008 | | | | | | | | | | | | | | | | | | | | | | | | | | | | | | | | | | | | | | | | | | | | | | | |
| DPB1*04:02 | | | 0.123 | | | | | | | | | | | | | | | | | | | | | | | | | | | | | | | | | | | | | | | | | | | | | | | |
| DPB1*10:01 | | | 0.004 | | | | | | | | | | | | | | | | | | | | | | | | | | | | | | | | | | | | | | | | | | | | | | | |
| DPB1*11:01 | | | 0.004 | | | | | | | | | | | | | | | | | | | | | | | | | | | | | | | | | | | | | | | | | | | | | | | |
| DPB1*13:01 | | | 0.023 | | | | | | | | | | | | | | | | | | | | | | | | | | | | | | | | | | | | | | | | | | | | | | | |
| DPB1*16:01 | | | 0.035 | | | | | | | | | | | | | | | | | | | | | | | | | | | | | | | | | | | | | | | | | | | | | | | |
| DPB1*17:01 | | | 0.046 | | | | | | | | | | | | | | | | | | | | | | | | | | | | | | | | | | | | | | | | | | | | | | | |
|  | | |  | | | | | | | | | | | | | | | | | | | | | | | | | | | | | | | | | | | | | | | | | | | | | | | |
| Cameroon | | | | | | | | | | | | | | | | | | | | | | | | | | | | | | | | | | | | | | | | | | | | | | | | | | |
|  | | | Cameroon (n=126) | | | | | | | | | Cameroon Bamileke (n=77) | | | | | | | | | | Cameroon Beti (n=174) | | | | | | | | | | | Cameroon Saa (n=172) | | | | | | | | | | Cameroon Yaounde (n=92) | | | | | | | |
| A*01:01 | | |  | | | | | | | | |  | | | | | | | | | | 0.011 | | | | | | | | | | |  | | | | | | | | | | 0.011 | | | | | | | |
| A*01:02 | | |  | | | | | | | | |  | | | | | | | | | | 0.003 | | | | | | | | | | |  | | | | | | | | | |  | | | | | | | |
| A*02:01 | | |  | | | | | | | | |  | | | | | | | | | | 0.112 | | | | | | | | | | |  | | | | | | | | | | 0.071 | | | | | | | |
| A*02:02 | | |  | | | | | | | | |  | | | | | | | | | | 0.060 | | | | | | | | | | |  | | | | | | | | | | 0.082 | | | | | | | |
| A*02:04 | | |  | | | | | | | | |  | | | | | | | | | |  | | | | | | | | | | |  | | | | | | | | | | 0.006 | | | | | | | |
| A*02:05 | | |  | | | | | | | | |  | | | | | | | | | | 0.011 | | | | | | | | | | |  | | | | | | | | | | 0.022 | | | | | | | |
| A*02:11 | | |  | | | | | | | | |  | | | | | | | | | |  | | | | | | | | | | |  | | | | | | | | | | 0.006 | | | | | | | |
| A*02:14 | | |  | | | | | | | | |  | | | | | | | | | |  | | | | | | | | | | |  | | | | | | | | | | 0.006 | | | | | | | |
| A*03:01 | | |  | | | | | | | | |  | | | | | | | | | | 0.086 | | | | | | | | | | |  | | | | | | | | | |  | | | | | | | |
| A*03:01:01 | | |  | | | | | | | | |  | | | | | | | | | |  | | | | | | | | | | |  | | | | | | | | | | 0.077 | | | | | | | |
|  | | |  | | | | | | | | |  | | | | | | | | | |  | | | | | | | | | | |  | | | | | | | | | |  | | | | | | | |
| B*07:02:01 | | |  | | | | | | | | |  | | | | | | | | | |  | | | | | | | | | | |  | | | | | | | | | | 0.060 | | | | | | | |
| B*08:01 | | |  | | | | | | | | |  | | | | | | | | | |  | | | | | | | | | | |  | | | | | | | | | | 0.054 | | | | | | | |
| B*13:02 | | |  | | | | | | | | |  | | | | | | | | | |  | | | | | | | | | | |  | | | | | | | | | | 0.027 | | | | | | | |
| B*14:01 | | |  | | | | | | | | |  | | | | | | | | | |  | | | | | | | | | | |  | | | | | | | | | | 0.011 | | | | | | | |
| B*14:02 | | |  | | | | | | | | |  | | | | | | | | | |  | | | | | | | | | | |  | | | | | | | | | | 0.005 | | | | | | | |
| B*14:03 | | |  | | | | | | | | |  | | | | | | | | | |  | | | | | | | | | | |  | | | | | | | | | | 0.016 | | | | | | | |
| B*15:01 | | |  | | | | | | | | |  | | | | | | | | | |  | | | | | | | | | | |  | | | | | | | | | | 0.011 | | | | | | | |
| B*15:03 | | |  | | | | | | | | |  | | | | | | | | | |  | | | | | | | | | | |  | | | | | | | | | | 0.049 | | | | | | | |
| B*15:10 | | |  | | | | | | | | |  | | | | | | | | | |  | | | | | | | | | | |  | | | | | | | | | | 0.011 | | | | | | | |
| B*15:16 | | |  | | | | | | | | |  | | | | | | | | | |  | | | | | | | | | | |  | | | | | | | | | | 0.011 | | | | | | | |
|  | | |  | | | | | | | | |  | | | | | | | | | |  | | | | | | | | | | |  | | | | | | | | | |  | | | | | | | |
| C*02:02 | | |  | | | | | | | | | 0.110 | | | | | | | | | | 0.046 | | | | | | | | | | |  | | | | | | | | | |  | | | | | | | |
| C*03:02 | | |  | | | | | | | | | 0.013 | | | | | | | | | | 0.023 | | | | | | | | | | |  | | | | | | | | | |  | | | | | | | |
| C*03:04 | | |  | | | | | | | | | 0.013 | | | | | | | | | | 0.032 | | | | | | | | | | |  | | | | | | | | | |  | | | | | | | |
| C*04:01 | | |  | | | | | | | | | 0.130 | | | | | | | | | | 0.187 | | | | | | | | | | |  | | | | | | | | | |  | | | | | | | |
| C*04:07 | | |  | | | | | | | | | 0.026 | | | | | | | | | | 0.014 | | | | | | | | | | |  | | | | | | | | | |  | | | | | | | |
| C*05:01 | | |  | | | | | | | | |  | | | | | | | | | | 0.023 | | | | | | | | | | |  | | | | | | | | | |  | | | | | | | |
| C*06:02 | | |  | | | | | | | | | 0.175 | | | | | | | | | | 0.126 | | | | | | | | | | |  | | | | | | | | | |  | | | | | | | |
| C*07:01 | | |  | | | | | | | | | 0.130 | | | | | | | | | | 0.129 | | | | | | | | | | |  | | | | | | | | | |  | | | | | | | |
| C*07:02 | | |  | | | | | | | | | 0.071 | | | | | | | | | | 0.066 | | | | | | | | | | |  | | | | | | | | | |  | | | | | | | |
| C*07:04 | | |  | | | | | | | | |  | | | | | | | | | | 0.003 | | | | | | | | | | |  | | | | | | | | | |  | | | | | | | |
|  | | |  | | | | | | | | |  | | | | | | | | | |  | | | | | | | | | | |  | | | | | | | | | |  | | | | | | | |
| DPA1*01:03 | | |  | | | | | | | | |  | | | | | | | | | |  | | | | | | | | | | | 0.372 | | | | | | | | | |  | | | | | | | |
| DPA1*02:01:01 | | |  | | | | | | | | |  | | | | | | | | | |  | | | | | | | | | | | 0.221 | | | | | | | | | |  | | | | | | | |
| DPA1*02:01:02 | | |  | | | | | | | | |  | | | | | | | | | |  | | | | | | | | | | | 0.003 | | | | | | | | | |  | | | | | | | |
| DPA1*02:01:03 | | |  | | | | | | | | |  | | | | | | | | | |  | | | | | | | | | | | 0.003 | | | | | | | | | |  | | | | | | | |
| DPA1*02:02:02 | | |  | | | | | | | | |  | | | | | | | | | |  | | | | | | | | | | | 0.096 | | | | | | | | | |  | | | | | | | |
| DPA1*03:01 | | |  | | | | | | | | |  | | | | | | | | | |  | | | | | | | | | | | 0.262 | | | | | | | | | |  | | | | | | | |
| DPA1*03:02 | | |  | | | | | | | | |  | | | | | | | | | |  | | | | | | | | | | | 0.017 | | | | | | | | | |  | | | | | | | |
| DPA1*04:01 | | |  | | | | | | | | |  | | | | | | | | | |  | | | | | | | | | | | 0.032 | | | | | | | | | |  | | | | | | | |
|  | | |  | | | | | | | | |  | | | | | | | | | |  | | | | | | | | | | |  | | | | | | | | | |  | | | | | | | |
| DPB1*01:01:01 | | |  | | | | | | | | |  | | | | | | | | | |  | | | | | | | | | | | 0.174 | | | | | | | | | |  | | | | | | | |
| DPB1*01:01:02 | | |  | | | | | | | | |  | | | | | | | | | |  | | | | | | | | | | | 0.038 | | | | | | | | | |  | | | | | | | |
| DPB1*02:01 | | |  | | | | | | | | |  | | | | | | | | | |  | | | | | | | | | | | 0.125 | | | | | | | | | |  | | | | | | | |
| DPB1*02:02 | | |  | | | | | | | | |  | | | | | | | | | |  | | | | | | | | | | | 0.009 | | | | | | | | | |  | | | | | | | |
| DPB1*03:01 | | |  | | | | | | | | |  | | | | | | | | | |  | | | | | | | | | | | 0.032 | | | | | | | | | |  | | | | | | | |
| DPB1*04:01 | | |  | | | | | | | | |  | | | | | | | | | |  | | | | | | | | | | | 0.110 | | | | | | | | | |  | | | | | | | |
| DPB1*04:02 | | |  | | | | | | | | |  | | | | | | | | | |  | | | | | | | | | | | 0.233 | | | | | | | | | |  | | | | | | | |
| DPB1*11:01:01 | | |  | | | | | | | | |  | | | | | | | | | |  | | | | | | | | | | | 0.009 | | | | | | | | | |  | | | | | | | |
| DPB1*13:01 | | |  | | | | | | | | |  | | | | | | | | | |  | | | | | | | | | | | 0.044 | | | | | | | | | |  | | | | | | | |
| DPB1*14:01 | | |  | | | | | | | | |  | | | | | | | | | |  | | | | | | | | | | | 0.003 | | | | | | | | | |  | | | | | | | |
|  | | |  | | | | | | | | |  | | | | | | | | | |  | | | | | | | | | | |  | | | | | | | | | |  | | | | | | | |
| DQA1*01:01 | | | 0.051 | | | | | | | | |  | | | | | | | | | |  | | | | | | | | | | |  | | | | | | | | | |  | | | | | | | |
| DQA1*01:02 | | | 0.380 | | | | | | | | |  | | | | | | | | | |  | | | | | | | | | | |  | | | | | | | | | |  | | | | | | | |
| DQA1*01:03 | | | 0.043 | | | | | | | | |  | | | | | | | | | |  | | | | | | | | | | |  | | | | | | | | | |  | | | | | | | |
| DQA1*01:04 | | | 0.071 | | | | | | | | |  | | | | | | | | | |  | | | | | | | | | | |  | | | | | | | | | |  | | | | | | | |
| DQA1*02:01 | | | 0.063 | | | | | | | | |  | | | | | | | | | |  | | | | | | | | | | |  | | | | | | | | | |  | | | | | | | |
| DQA1*03:01 | | | 0.004 | | | | | | | | |  | | | | | | | | | |  | | | | | | | | | | |  | | | | | | | | | |  | | | | | | | |
| DQA1*03:02 | | | 0.111 | | | | | | | | |  | | | | | | | | | |  | | | | | | | | | | |  | | | | | | | | | |  | | | | | | | |
| DQA1*04:01 | | | 0.111 | | | | | | | | |  | | | | | | | | | |  | | | | | | | | | | |  | | | | | | | | | |  | | | | | | | |
| DQA1*05:01 | | | 0.158 | | | | | | | | |  | | | | | | | | | |  | | | | | | | | | | |  | | | | | | | | | |  | | | | | | | |
| DQA1*06:01 | | | 0.004 | | | | | | | | |  | | | | | | | | | |  | | | | | | | | | | |  | | | | | | | | | |  | | | | | | | |
|  | | |  | | | | | | | | |  | | | | | | | | | |  | | | | | | | | | | |  | | | | | | | | | |  | | | | | | | |
| DQB1*02:01 | | | 0.071 | | | | | | | | |  | | | | | | | | | |  | | | | | | | | | | |  | | | | | | | | | |  | | | | | | | |
| DQB1*02:02 | | | 0.071 | | | | | | | | |  | | | | | | | | | |  | | | | | | | | | | |  | | | | | | | | | |  | | | | | | | |
| DQB1*03:01 | | | 0.130 | | | | | | | | |  | | | | | | | | | |  | | | | | | | | | | |  | | | | | | | | | |  | | | | | | | |
| DQB1*03:02 | | | 0.015 | | | | | | | | |  | | | | | | | | | |  | | | | | | | | | | |  | | | | | | | | | |  | | | | | | | |
| DQB1*03:03:02 | | | 0.087 | | | | | | | | |  | | | | | | | | | |  | | | | | | | | | | |  | | | | | | | | | |  | | | | | | | |
| DQB1*04:01 | | | 0.004 | | | | | | | | |  | | | | | | | | | |  | | | | | | | | | | |  | | | | | | | | | |  | | | | | | | |
| DQB1*04:02 | | | 0.071 | | | | | | | | |  | | | | | | | | | |  | | | | | | | | | | |  | | | | | | | | | |  | | | | | | | |
| DQB1*05:01 | | | 0.119 | | | | | | | | |  | | | | | | | | | |  | | | | | | | | | | |  | | | | | | | | | |  | | | | | | | |
| DQB1*05:02 | | | 0.011 | | | | | | | | |  | | | | | | | | | |  | | | | | | | | | | |  | | | | | | | | | |  | | | | | | | |
| DQB1*05:03:01 | | | 0.011 | | | | | | | | |  | | | | | | | | | |  | | | | | | | | | | |  | | | | | | | | | |  | | | | | | | |
|  | | |  | | | | | | | | |  | | | | | | | | | |  | | | | | | | | | | |  | | | | | | | | | |  | | | | | | | |
| DRB1*01:01 | | | 0.004 | | | | | | | | |  | | | | | | | | | |  | | | | | | | | | | |  | | | | | | | | | |  | | | | | | | |
| DRB1*01:02 | | | 0.048 | | | | | | | | |  | | | | | | | | | |  | | | | | | | | | | |  | | | | | | | | | |  | | | | | | | |
| DRB1*03:01:01 | | | 0.071 | | | | | | | | |  | | | | | | | | | |  | | | | | | | | | | |  | | | | | | | | | |  | | | | | | | |
| DRB1*03:02:01 | | | 0.067 | | | | | | | | |  | | | | | | | | | |  | | | | | | | | | | |  | | | | | | | | | |  | | | | | | | |
| DRB1*04:05:01 | | | 0.008 | | | | | | | | |  | | | | | | | | | |  | | | | | | | | | | |  | | | | | | | | | |  | | | | | | | |
| DRB1*07:01 | | | 0.055 | | | | | | | | |  | | | | | | | | | |  | | | | | | | | | | |  | | | | | | | | | |  | | | | | | | |
| DRB1*08:04:01 | | | 0.067 | | | | | | | | |  | | | | | | | | | |  | | | | | | | | | | |  | | | | | | | | | |  | | | | | | | |
| DRB1*09:01:02 | | | 0.004 | | | | | | | | |  | | | | | | | | | |  | | | | | | | | | | |  | | | | | | | | | |  | | | | | | | |
| DRB1*10:01 | | | 0.011 | | | | | | | | |  | | | | | | | | | |  | | | | | | | | | | |  | | | | | | | | | |  | | | | | | | |
| DRB1*11:01 | | | 0.048 | | | | | | | | |  | | | | | | | | | |  | | | | | | | | | | |  | | | | | | | | | |  | | | | | | | |
| Central African Republic | | | | | | | | | | | | | | | | | | | | | | | | | | | | | | | | | | | | | | | | | | | | | | | | | | |
|  | | | Central African Republic Aka Pygmy (n=93) | | | | | | | | | | | | | | | | | | | | | | | | | | | | | | | | | | | | | | | | | | | | | | | |
| DPB1*01:01 | | | 0.049 | | | | | | | | | | | | | | | | | | | | | | | | | | | | | | | | | | | | | | | | | | | | | | | |
| DPB1*02:01 | | | 0.030 | | | | | | | | | | | | | | | | | | | | | | | | | | | | | | | | | | | | | | | | | | | | | | | |
| DPB1*03:01 | | | 0.025 | | | | | | | | | | | | | | | | | | | | | | | | | | | | | | | | | | | | | | | | | | | | | | | |
| DPB1*04:01 | | | 0.031 | | | | | | | | | | | | | | | | | | | | | | | | | | | | | | | | | | | | | | | | | | | | | | | |
| DPB1*04:02 | | | 0.695 | | | | | | | | | | | | | | | | | | | | | | | | | | | | | | | | | | | | | | | | | | | | | | | |
| DPB1*13:01 | | | 0.006 | | | | | | | | | | | | | | | | | | | | | | | | | | | | | | | | | | | | | | | | | | | | | | | |
| DPB1*18:01 | | | 0.049 | | | | | | | | | | | | | | | | | | | | | | | | | | | | | | | | | | | | | | | | | | | | | | | |
| DPB1*19:01 | | | 0.006 | | | | | | | | | | | | | | | | | | | | | | | | | | | | | | | | | | | | | | | | | | | | | | | |
| DPB1*34:01 | | | 0.006 | | | | | | | | | | | | | | | | | | | | | | | | | | | | | | | | | | | | | | | | | | | | | | | |
| DPB1*39:01 | | | 0.068 | | | | | | | | | | | | | | | | | | | | | | | | | | | | | | | | | | | | | | | | | | | | | | | |
|  | | |  | | | | | | | | | | | | | | | | | | | | | | | | | | | | | | | | | | | | | | | | | | | | | | | |
| DQB1*02:01 | | | 0.369 | | | | | | | | | | | | | | | | | | | | | | | | | | | | | | | | | | | | | | | | | | | | | | | |
| DQB1*03:01 | | | 0.030 | | | | | | | | | | | | | | | | | | | | | | | | | | | | | | | | | | | | | | | | | | | | | | | |
| DQB1*03:02 | | | 0.036 | | | | | | | | | | | | | | | | | | | | | | | | | | | | | | | | | | | | | | | | | | | | | | | |
| DQB1*03:03:02 | | | 0.024 | | | | | | | | | | | | | | | | | | | | | | | | | | | | | | | | | | | | | | | | | | | | | | | |
| DQB1*04:02 | | | 0.012 | | | | | | | | | | | | | | | | | | | | | | | | | | | | | | | | | | | | | | | | | | | | | | | |
| DQB1*05:01 | | | 0.208 | | | | | | | | | | | | | | | | | | | | | | | | | | | | | | | | | | | | | | | | | | | | | | | |
| DQB1*06:01 | | | 0.006 | | | | | | | | | | | | | | | | | | | | | | | | | | | | | | | | | | | | | | | | | | | | | | | |
| DQB1*06:02 | | | 0.190 | | | | | | | | | | | | | | | | | | | | | | | | | | | | | | | | | | | | | | | | | | | | | | | |
| DQB1*06:03 | | | 0.036 | | | | | | | | | | | | | | | | | | | | | | | | | | | | | | | | | | | | | | | | | | | | | | | |
| DQB1*06:04 | | | 0.065 | | | | | | | | | | | | | | | | | | | | | | | | | | | | | | | | | | | | | | | | | | | | | | | |
|  | | |  | | | | | | | | | | | | | | | | | | | | | | | | | | | | | | | | | | | | | | | | | | | | | | | |
| DRB1*01:01 | | | 0.006 | | | | | | | | | | | | | | | | | | | | | | | | | | | | | | | | | | | | | | | | | | | | | | | |
| DRB1*03:01 | | | 0.125 | | | | | | | | | | | | | | | | | | | | | | | | | | | | | | | | | | | | | | | | | | | | | | | |
| DRB1*04:03 | | | 0.006 | | | | | | | | | | | | | | | | | | | | | | | | | | | | | | | | | | | | | | | | | | | | | | | |
| DRB1*04:05 | | | 0.006 | | | | | | | | | | | | | | | | | | | | | | | | | | | | | | | | | | | | | | | | | | | | | | | |
| DRB1*07:01 | | | 0.244 | | | | | | | | | | | | | | | | | | | | | | | | | | | | | | | | | | | | | | | | | | | | | | | |
| DRB1*09 | | | 0.006 | | | | | | | | | | | | | | | | | | | | | | | | | | | | | | | | | | | | | | | | | | | | | | | |
| DRB1*10:01 | | | 0.006 | | | | | | | | | | | | | | | | | | | | | | | | | | | | | | | | | | | | | | | | | | | | | | | |
| DRB1*11:01 | | | 0.006 | | | | | | | | | | | | | | | | | | | | | | | | | | | | | | | | | | | | | | | | | | | | | | | |
| DRB1*11:02 | | | 0.054 | | | | | | | | | | | | | | | | | | | | | | | | | | | | | | | | | | | | | | | | | | | | | | | |
| DRB1*11:07 | | | 0.006 | | | | | | | | | | | | | | | | | | | | | | | | | | | | | | | | | | | | | | | | | | | | | | | |
| Gabon | | | | | | | | | | | | | | | | | | | | | | | | | | | | | | | | | | | | | | | | | | | | | | | | | | |
|  | | | Gabon Haut-Ogooue Dienga (n=167) | | | | | | | | | | | | | | | | | | | | | | | Gabon Lambarene (n=120) | | | | | | | | | | | | | | | | | | | | | | | | |
| DPA1*01:03 | | |  | | | | | | | | | | | | | | | | | | | | | | | 0.425 | | | | | | | | | | | | | | | | | | | | | | | | |
| DPA1*02:01:01 | | |  | | | | | | | | | | | | | | | | | | | | | | | 0.183 | | | | | | | | | | | | | | | | | | | | | | | | |
| DPA1*02:01:02 | | |  | | | | | | | | | | | | | | | | | | | | | | | 0.008 | | | | | | | | | | | | | | | | | | | | | | | | |
| DPA1*02:02:01 | | |  | | | | | | | | | | | | | | | | | | | | | | | 0.013 | | | | | | | | | | | | | | | | | | | | | | | | |
| DPA1*02:02:02 | | |  | | | | | | | | | | | | | | | | | | | | | | | 0.125 | | | | | | | | | | | | | | | | | | | | | | | | |
| DPA1*03:01 | | |  | | | | | | | | | | | | | | | | | | | | | | | 0.208 | | | | | | | | | | | | | | | | | | | | | | | | |
| DPA1*04:01 | | |  | | | | | | | | | | | | | | | | | | | | | | | 0.038 | | | | | | | | | | | | | | | | | | | | | | | | |
|  | | |  | | | | | | | | | | | | | | | | | | | | | | |  | | | | | | | | | | | | | | | | | | | | | | | | |
| DPB1*01:01 | | | 0.234 | | | | | | | | | | | | | | | | | | | | | | | 0.208 | | | | | | | | | | | | | | | | | | | | | | | | |
| DPB1*02:01 | | | 0.132 | | | | | | | | | | | | | | | | | | | | | | | 0.183 | | | | | | | | | | | | | | | | | | | | | | | | |
| DPB1*03:01 | | | 0.093 | | | | | | | | | | | | | | | | | | | | | | | 0.067 | | | | | | | | | | | | | | | | | | | | | | | | |
| DPB1*04:01 | | | 0.063 | | | | | | | | | | | | | | | | | | | | | | | 0.046 | | | | | | | | | | | | | | | | | | | | | | | | |
| DPB1*04:02 | | | 0.290 | | | | | | | | | | | | | | | | | | | | | | | 0.271 | | | | | | | | | | | | | | | | | | | | | | | | |
| DPB1*06:01 | | |  | | | | | | | | | | | | | | | | | | | | | | | 0.013 | | | | | | | | | | | | | | | | | | | | | | | | |
| DPB1*10:01 | | |  | | | | | | | | | | | | | | | | | | | | | | | 0.004 | | | | | | | | | | | | | | | | | | | | | | | | |
| DPB1*11:01 | | | 0.012 | | | | | | | | | | | | | | | | | | | | | | | 0.021 | | | | | | | | | | | | | | | | | | | | | | | | |
| DPB1*13:01 | | | 0.030 | | | | | | | | | | | | | | | | | | | | | | | 0.008 | | | | | | | | | | | | | | | | | | | | | | | | |
| DPB1*14:01 | | |  | | | | | | | | | | | | | | | | | | | | | | | 0.008 | | | | | | | | | | | | | | | | | | | | | | | | |
|  | | |  | | | | | | | | | | | | | | | | | | | | | | |  | | | | | | | | | | | | | | | | | | | | | | | | |
| DQA1*01:01 | | | 0.150 | | | | | | | | | | | | | | | | | | | | | | |  | | | | | | | | | | | | | | | | | | | | | | | | |
| DQA1*01:02 | | | 0.500 | | | | | | | | | | | | | | | | | | | | | | |  | | | | | | | | | | | | | | | | | | | | | | | | |
| DQA1*01:03 | | | 0.039 | | | | | | | | | | | | | | | | | | | | | | |  | | | | | | | | | | | | | | | | | | | | | | | | |
| DQA1*02:01 | | | 0.051 | | | | | | | | | | | | | | | | | | | | | | |  | | | | | | | | | | | | | | | | | | | | | | | | |
| DQA1*03:01 | | | 0.075 | | | | | | | | | | | | | | | | | | | | | | |  | | | | | | | | | | | | | | | | | | | | | | | | |
| DQA1*04:01 | | | 0.009 | | | | | | | | | | | | | | | | | | | | | | |  | | | | | | | | | | | | | | | | | | | | | | | | |
| DQA1*05:01 | | | 0.177 | | | | | | | | | | | | | | | | | | | | | | |  | | | | | | | | | | | | | | | | | | | | | | | | |
|  | | |  | | | | | | | | | | | | | | | | | | | | | | |  | | | | | | | | | | | | | | | | | | | | | | | | |
| DQB1*02:01 | | | 0.168 | | | | | | | | | | | | | | | | | | | | | | |  | | | | | | | | | | | | | | | | | | | | | | | | |
| DQB1*03:01 | | | 0.102 | | | | | | | | | | | | | | | | | | | | | | |  | | | | | | | | | | | | | | | | | | | | | | | | |
| DQB1*03:03 | | | 0.030 | | | | | | | | | | | | | | | | | | | | | | |  | | | | | | | | | | | | | | | | | | | | | | | | |
| DQB1*03:04 | | | 0.003 | | | | | | | | | | | | | | | | | | | | | | |  | | | | | | | | | | | | | | | | | | | | | | | | |
| DQB1*04:02 | | | 0.009 | | | | | | | | | | | | | | | | | | | | | | |  | | | | | | | | | | | | | | | | | | | | | | | | |
| DQB1*05:01 | | | 0.147 | | | | | | | | | | | | | | | | | | | | | | |  | | | | | | | | | | | | | | | | | | | | | | | | |
| DQB1*05:02 | | | 0.006 | | | | | | | | | | | | | | | | | | | | | | |  | | | | | | | | | | | | | | | | | | | | | | | | |
| DQB1*05:03 | | | 0.003 | | | | | | | | | | | | | | | | | | | | | | |  | | | | | | | | | | | | | | | | | | | | | | | | |
| DQB1*06:02 | | | 0.422 | | | | | | | | | | | | | | | | | | | | | | |  | | | | | | | | | | | | | | | | | | | | | | | | |
| DQB1*06:03 | | | 0.021 | | | | | | | | | | | | | | | | | | | | | | |  | | | | | | | | | | | | | | | | | | | | | | | | |
| Japan | | | | | | | | | | | | | | | | | | | | | | | | | | | | | | | | | | | | | | | | | | | | | | | | | | |
|  | J1 | | J2 | | J3 | | J4 | | J5 | | J6 | | J7 | | J8 | | | J9 | | J10 | | | J11 | | J12 | | J13 | | | J14 | | J15 | | | J16 | | J17 | | J18 | | J19 | | | J20 | | J21 | | J22 | | J23 |
| A*01:01 |  | | 0.009 | |  | |  | |  | |  | |  | |  | | |  | |  | | |  | | 0.00401 | |  | | |  | | 0.002 | | |  | | 0.018 | |  | |  | | |  | |  | |  | |  |
| A*01:01:01 |  | |  | |  | |  | |  | |  | |  | |  | | | 0.004 | |  | | |  | |  | |  | | |  | |  | | |  | |  | |  | |  | | |  | |  | |  | |  |
| A*02:01 |  | | 0.115 | |  | | 0.200 | |  | |  | |  | |  | | |  | |  | | |  | | 0.116 | |  | | |  | | 0.116 | | |  | | 0.106 | |  | |  | | |  | |  | |  | |  |
| A*02:01:01 |  | |  | |  | |  | |  | |  | |  | |  | | | 0.102 | |  | | |  | |  | |  | | |  | |  | | |  | |  | |  | |  | | |  | |  | |  | |  |
| A*02:01:03 |  | |  | |  | |  | |  | |  | |  | |  | | | 0.014 | |  | | |  | |  | |  | | |  | |  | | |  | |  | |  | |  | | |  | |  | |  | |  |
| A*02:03 |  | |  | |  | |  | |  | |  | |  | |  | | |  | |  | | |  | | 0.00063 | |  | | |  | | 0.001 | | |  | |  | |  | |  | | |  | |  | |  | |  |
| A*02:04 |  | |  | |  | |  | |  | |  | |  | |  | | | 0.014 | |  | | |  | |  | |  | | |  | |  | | |  | |  | |  | |  | | |  | |  | |  | |  |
| A*02:05 |  | |  | |  | |  | |  | |  | |  | |  | | |  | |  | | |  | | 0.00005 | |  | | |  | |  | | |  | |  | |  | |  | | |  | |  | |  | |  |
| A*02:06 |  | | 0.077 | |  | | 0.200 | |  | |  | |  | |  | | | 0.183 | |  | | |  | | 0.091 | |  | | |  | | 0.087 | | |  | | 0.084 | |  | |  | | |  | |  | |  | |  |
| A*02:07 |  | | 0.022 | |  | | 0.010 | |  | |  | |  | |  | | | 0.007 | |  | | |  | | 0.035 | |  | | |  | | 0.034 | | |  | | 0.040 | |  | |  | | |  | |  | |  | |  |
|  |  | |  | |  | |  | |  | |  | |  | |  | | |  | |  | | |  | |  | |  | | |  | |  | | |  | |  | |  | |  | | |  | |  | |  | |  |
| B*07:02 |  | | 0.065 | |  | | 0.010 | |  | |  | |  | |  | | |  | |  | | |  | | 0.056 | |  | | |  | | 0.057 | | |  | | 0.052 | |  | |  | | |  | |  | |  | |  |
| B*07:05 |  | |  | |  | |  | |  | |  | |  | |  | | |  | |  | | |  | | 0.00026 | |  | | |  | |  | | |  | |  | |  | |  | | |  | |  | |  | |  |
| B*07:31 |  | |  | |  | |  | |  | |  | |  | |  | | |  | |  | | |  | | 0.00005 | |  | | |  | |  | | |  | |  | |  | |  | | |  | |  | |  | |  |
| B*08:01 |  | |  | |  | |  | |  | |  | |  | |  | | |  | |  | | |  | | 0.00016 | |  | | |  | |  | | |  | |  | |  | |  | | |  | |  | |  | |  |
| B*13:01 |  | | 0.015 | |  | |  | |  | |  | |  | |  | | |  | |  | | |  | | 0.012 | |  | | |  | | 0.013 | | |  | | 0.013 | |  | |  | | |  | |  | |  | |  |
| B*13:02 |  | | 0.003 | |  | |  | |  | |  | |  | |  | | |  | |  | | |  | | 0.00287 | |  | | |  | | 0.002 | | |  | | 0.004 | |  | |  | | |  | |  | |  | |  |
| B*14:01 |  | |  | |  | |  | |  | |  | |  | |  | | |  | |  | | |  | | 0.00016 | |  | | |  | |  | | |  | |  | |  | |  | | |  | |  | |  | |  |
| B*14:02 |  | |  | |  | |  | |  | |  | |  | |  | | |  | |  | | |  | | 0.00010 | |  | | |  | |  | | |  | |  | |  | |  | | |  | |  | |  | |  |
| B*15:01 |  | | 0.087 | |  | | 0.290 | |  | |  | |  | |  | | |  | |  | | |  | | 0.076 | |  | | |  | | 0.075 | | |  | | 0.065 | |  | |  | | |  | |  | |  | |  |
| B*15:02 |  | | 0.001 | |  | |  | |  | |  | |  | |  | | |  | |  | | |  | | 0.00031 | |  | | |  | | 0.001 | | |  | |  | |  | |  | | |  | |  | |  | |  |
|  |  | |  | |  | |  | |  | |  | |  | |  | | |  | |  | | |  | |  | |  | | |  | |  | | |  | |  | |  | |  | | |  | |  | |  | |  |
| C*01:02 |  | | 0.148 | |  | |  | |  | |  | |  | |  | | |  | | 0.182 | | |  | | 0.176 | |  | | |  | | 0.182 | | |  | | 0.170 | |  | |  | | |  | |  | |  | | 0.173 |
| C*01:03 |  | | 0.004 | |  | |  | |  | |  | |  | |  | | |  | | 0.005 | | |  | | 0.00318 | |  | | |  | | 0.004 | | |  | |  | |  | |  | | |  | |  | |  | |  |
| C*02:02 |  | |  | |  | |  | |  | |  | |  | |  | | |  | |  | | |  | | 0.00026 | |  | | |  | |  | | |  | |  | |  | |  | | |  | |  | |  | | 0.00154 |
| C*03:02 |  | | 0.004 | |  | |  | |  | |  | |  | |  | | |  | | 0.006 | | |  | | 0.00568 | |  | | |  | | 0.006 | | |  | |  | |  | |  | | |  | |  | |  | | 0.00758 |
| C*03:03 |  | | 0.121 | |  | |  | |  | |  | |  | |  | | |  | | 0.129 | | |  | | 0.131 | |  | | |  | | 0.130 | | |  | | 0.078 | |  | |  | | |  | |  | |  | | 0.149 |
| C*03:04 |  | | 0.137 | |  | |  | |  | |  | |  | |  | | |  | | 0.127 | | |  | | 0.124 | |  | | |  | | 0.130 | | |  | | 0.113 | |  | |  | | |  | |  | |  | | 0.127 |
| C*03:23 |  | |  | |  | |  | |  | |  | |  | |  | | |  | |  | | |  | | 0.00010 | |  | | |  | |  | | |  | |  | |  | |  | | |  | |  | |  | |  |
| C*03:43 |  | |  | |  | |  | |  | |  | |  | |  | | |  | |  | | |  | | 0.00010 | |  | | |  | |  | | |  | |  | |  | |  | | |  | |  | |  | |  |
| C*03:64 |  | |  | |  | |  | |  | |  | |  | |  | | |  | |  | | |  | | 0.00005 | |  | | |  | |  | | |  | |  | |  | |  | | |  | |  | |  | |  |
| C*04:01 |  | | 0.046 | |  | |  | |  | |  | |  | |  | | |  | |  | | |  | |  | |  | | |  | |  | | |  | |  | |  | |  | | |  | |  | |  | |  |
|  |  | |  | |  | |  | |  | |  | |  | |  | | |  | |  | | |  | |  | |  | | |  | |  | | |  | |  | |  | |  | | |  | |  | |  | |  |
| DPA1*01:03 |  | |  | | 0.331 | |  | |  | |  | |  | |  | | |  | |  | | |  | |  | | 0.403 | | |  | |  | | |  | |  | |  | |  | | |  | |  | |  | |  |
| DPA1*01:11 |  | |  | |  | |  | |  | |  | |  | |  | | |  | |  | | |  | |  | | 0.00030 | | |  | |  | | |  | |  | |  | |  | | |  | |  | |  | |  |
| DPA1*02:01 |  | |  | |  | |  | |  | |  | |  | |  | | |  | |  | | |  | |  | | 0.160 | | |  | |  | | |  | |  | |  | |  | | |  | |  | |  | |  |
| DPA1*02:01:01 |  | |  | | 0.180 | |  | |  | |  | |  | |  | | |  | |  | | |  | |  | |  | | |  | |  | | |  | |  | |  | |  | | |  | |  | |  | |  |
| DPA1*02:02 |  | |  | |  | |  | |  | |  | |  | |  | | |  | |  | | |  | |  | | 0.435 | | |  | |  | | |  | |  | |  | |  | | |  | |  | |  | |  |
| DPA1*02:02:01 |  | |  | | 0.006 | |  | |  | |  | |  | |  | | |  | |  | | |  | |  | |  | | |  | |  | | |  | |  | |  | |  | | |  | |  | |  | |  |
| DPA1*02:02:02 |  | |  | | 0.483 | |  | |  | |  | |  | |  | | |  | |  | | |  | |  | |  | | |  | |  | | |  | |  | |  | |  | | |  | |  | |  | |  |
| DPA1*04:01 |  | |  | |  | |  | |  | |  | |  | |  | | |  | |  | | |  | |  | |  | | |  | |  | | |  | |  | |  | |  | | |  | |  | |  | |  |
|  |  | |  | |  | |  | |  | |  | |  | |  | | |  | |  | | |  | |  | |  | | |  | |  | | |  | |  | |  | |  | | |  | |  | |  | |  |
| DPB1*01:01 |  | |  | |  | |  | |  | |  | |  | |  | | |  | |  | | |  | |  | | 0.00030 | | |  | |  | | |  | |  | |  | |  | | |  | |  | |  | |  |
| DPB1*02:01 | 0.250 | |  | | 0.227 | |  | |  | |  | |  | |  | | |  | |  | | |  | |  | | 0.238 | | |  | |  | | |  | |  | |  | |  | | |  | | 0.205 | |  | |  |
| DPB1*02:02 | 0.050 | |  | | 0.035 | |  | |  | |  | |  | |  | | |  | |  | | |  | |  | | 0.039 | | |  | |  | | |  | |  | |  | |  | | |  | | 0.015 | |  | |  |
| DPB1*03:01 | 0.030 | |  | | 0.017 | |  | |  | |  | |  | |  | | |  | |  | | |  | |  | | 0.051 | | |  | |  | | |  | |  | |  | |  | | |  | | 0.057 | |  | |  |
| DPB1*04:01 | 0.050 | |  | | 0.041 | |  | |  | |  | |  | |  | | |  | |  | | |  | |  | | 0.050 | | |  | |  | | |  | |  | |  | |  | | |  | | 0.023 | |  | |  |
| DPB1*04:02 | 0.140 | |  | | 0.076 | |  | |  | |  | |  | |  | | |  | |  | | |  | |  | | 0.098 | | |  | |  | | |  | |  | |  | |  | | |  | | 0.170 | |  | |  |
| DPB1*05:01 | 0.350 | |  | | 0.442 | |  | |  | |  | |  | |  | | |  | |  | | |  | |  | | 0.374 | | |  | |  | | |  | |  | |  | |  | | |  | | 0.413 | |  | |  |
| DPB1*06:01 | 0.010 | |  | | 0.012 | |  | |  | |  | |  | |  | | |  | |  | | |  | |  | | 0.00520 | | |  | |  | | |  | |  | |  | |  | | |  | | 0.004 | |  | |  |
| DPB1*09:01 | 0.100 | |  | | 0.110 | |  | |  | |  | |  | |  | | |  | |  | | |  | |  | | 0.103 | | |  | |  | | |  | |  | |  | |  | | |  | | 0.095 | |  | |  |
|  |  | |  | |  | |  | |  | |  | |  | |  | | |  | |  | | |  | |  | |  | | |  | |  | | |  | |  | |  | |  | | |  | |  | |  | |  |
| DQA1*01:01 |  | |  | |  | |  | |  | |  | |  | |  | | |  | |  | | |  | |  | | 0.066 | | | 0.123 | |  | | |  | |  | |  | |  | | | 0.156 | |  | |  | |  |
| DQA1*01:02 |  | |  | |  | |  | |  | |  | |  | |  | | |  | |  | | |  | |  | | 0.134 | | | 0.121 | |  | | |  | |  | |  | |  | | | 0.112 | |  | |  | |  |
| DQA1*01:03 |  | |  | |  | |  | |  | |  | |  | |  | | |  | |  | | |  | |  | | 0.192 | | | 0.169 | |  | | |  | |  | |  | |  | | | 0.172 | |  | |  | |  |
| DQA1*01:04 |  | |  | |  | |  | |  | |  | |  | |  | | |  | |  | | |  | |  | | 0.047 | | |  | |  | | |  | |  | |  | |  | | |  | |  | |  | |  |
| DQA1*01:05 |  | |  | |  | |  | |  | |  | |  | |  | | |  | |  | | |  | |  | | 0.00550 | | |  | |  | | |  | |  | |  | |  | | |  | |  | |  | |  |
| DQA1*02:01 |  | |  | |  | |  | |  | |  | |  | |  | | |  | |  | | |  | |  | | 0.00360 | | | 0.008 | |  | | |  | |  | |  | |  | | |  | |  | |  | |  |
| DQA1*03:01 |  | |  | |  | |  | |  | |  | |  | |  | | |  | |  | | |  | |  | | 0.110 | | | 0.423 | |  | | |  | |  | |  | |  | | | 0.417 | |  | |  | |  |
| DQA1*03:02 |  | |  | |  | |  | |  | |  | |  | |  | | |  | |  | | |  | |  | | 0.144 | | |  | |  | | |  | |  | |  | |  | | |  | |  | |  | |  |
| DQA1*03:03 |  | |  | |  | |  | |  | |  | |  | |  | | |  | |  | | |  | |  | | 0.166 | | |  | |  | | |  | |  | |  | |  | | |  | |  | |  | |  |
| DQA1*04:01 |  | |  | |  | |  | |  | |  | |  | |  | | |  | |  | | |  | |  | | 0.028 | | | 0.031 | |  | | |  | |  | |  | |  | | | 0.036 | |  | |  | |  |
|  |  | |  | |  | |  | |  | |  | |  | |  | | |  | |  | | |  | |  | |  | | |  | |  | | |  | |  | |  | |  | | |  | |  | |  | |  |
| DQB1*02 |  | |  | | 0.012 | |  | |  | |  | |  | |  | | |  | |  | | |  | |  | |  | | |  | |  | | |  | |  | |  | |  | | |  | |  | |  | |  |
| DQB1*02:01 |  | |  | |  | |  | |  | |  | |  | |  | | |  | |  | | |  | |  | | 0.00160 | | |  | |  | | |  | |  | |  | |  | | |  | |  | |  | |  |
| DQB1*02:02 |  | | 0.003 | |  | |  | |  | |  | |  | |  | | |  | |  | | |  | |  | | 0.00260 | | |  | |  | | |  | |  | |  | |  | | |  | |  | |  | |  |
| DQB1*03:01 | 0.110 | | 0.113 | | 0.099 | |  | |  | |  | |  | |  | | |  | |  | | |  | |  | | 0.112 | | |  | |  | | |  | |  | |  | |  | | |  | |  | |  | |  |
| DQB1*03:02 | 0.090 | | 0.108 | | 0.081 | |  | |  | |  | |  | |  | | |  | |  | | |  | |  | | 0.108 | | |  | |  | | |  | |  | |  | |  | | |  | |  | |  | |  |
| DQB1*03:03 | 0.130 | | 0.135 | |  | |  | |  | |  | |  | |  | | |  | |  | | |  | |  | | 0.150 | | |  | |  | | |  | |  | |  | |  | | |  | |  | |  | |  |
| DQB1*03:03:02 |  | |  | | 0.174 | |  | |  | |  | |  | |  | | |  | |  | | |  | |  | |  | | |  | |  | | |  | |  | |  | |  | | |  | |  | |  | |  |
| DQB1*03:06 |  | | 0.001 | |  | |  | |  | |  | |  | |  | | |  | |  | | |  | |  | |  | | |  | |  | | |  | |  | |  | |  | | |  | |  | |  | |  |
| DQB1*03:19 |  | |  | |  | |  | |  | |  | |  | |  | | |  | |  | | |  | |  | | 0.00030 | | |  | |  | | |  | |  | |  | |  | | |  | |  | |  | |  |
| DQB1*04:01 | 0.120 | | 0.115 | | 0.163 | |  | |  | |  | |  | |  | | |  | |  | | |  | |  | |  | | |  | |  | | |  | |  | |  | |  | | |  | |  | |  | |  |
| DRB1*01:01 | 0.070 | | 0.065 | |  | |  | |  | | 0.074 | | 0.048 | |  | | |  | |  | | |  | | 0.058 | | 0.065 | | |  | | 0.056 | | |  | |  | |  | |  | | |  | |  | |  | |  |
| DRB1*01:02 |  | |  | |  | |  | |  | |  | |  | |  | | |  | |  | | |  | | 0.00016 | |  | | |  | |  | | |  | |  | |  | |  | | |  | |  | |  | |  |
| DRB1*03:01 |  | |  | |  | |  | |  | |  | |  | |  | | |  | |  | | |  | | 0.00146 | | 0.00070 | | |  | | 0.001 | | |  | |  | |  | |  | | |  | |  | |  | |  |
| DRB1*04:01 | 0.020 | | 0.007 | |  | |  | |  | | 0.005 | | 0.003 | |  | | |  | |  | | |  | | 0.00954 | | 0.00910 | | |  | | 0.013 | | |  | |  | |  | |  | | |  | |  | |  | |  |
| DRB1*04:02 |  | |  | |  | |  | |  | |  | |  | |  | | |  | |  | | |  | | 0.00005 | |  | | |  | |  | | |  | |  | |  | |  | | |  | |  | |  | |  |
| DRB1*04:03 | 0.040 | | 0.040 | |  | |  | |  | | 0.028 | | 0.018 | |  | | |  | |  | | |  | | 0.031 | | 0.037 | | |  | | 0.030 | | |  | |  | |  | |  | | |  | |  | |  | |  |
| DRB1*04:04 |  | | 0.001 | |  | |  | |  | | 0.008 | |  | |  | | |  | |  | | |  | | 0.00255 | | 0.00160 | | |  | | 0.003 | | |  | |  | |  | |  | | |  | |  | |  | |  |
| DRB1*04:05 | 0.140 | | 0.115 | |  | |  | |  | | 0.128 | | 0.155 | |  | | |  | |  | | |  | | 0.135 | | 0.137 | | |  | | 0.140 | | |  | |  | |  | |  | | |  | |  | |  | |  |
| DRB1*04:06 | 0.040 | | 0.035 | |  | |  | |  | | 0.018 | | 0.024 | |  | | |  | |  | | |  | | 0.034 | | 0.039 | | |  | | 0.035 | | |  | |  | |  | |  | | |  | |  | |  | |  |
| DRB1*04:07 |  | | 0.009 | |  | |  | |  | | 0.008 | | 0.003 | |  | | |  | |  | | |  | | 0.00573 | | 0.00520 | | |  | | 0.005 | | |  | |  | |  | |  | | |  | |  | |  | |  |
| Papua New Guinea | | | | | | | | | | | | | | | | | | | | | | | | | | | | | | | | | | | | | | | | | | | | | | | | | | |
|  | P1 | P2 | | | | P3 | | P4 | | P5 | | | | P6 | | P7 | | | P8 | | P9 | | | P10 | | | | P11 | | | P12 | | | P13 | | P14 | | P15 | | | | P16 | | | P17 | | P18 | | P19 | |
| A*11:01 |  | | |  | |  | |  | |  | | | |  | |  | | | 0.179 | |  | | | 0.636 | | | |  | | |  | | |  | |  | |  | | | | 0.550 | | |  | |  | | 0.385 | |
| A*24:02 |  | | |  | |  | |  | |  | | | |  | |  | | | 0.744 | |  | | | 0.153 | | | |  | | |  | | |  | |  | |  | | | | 0.142 | | |  | |  | | 0.513 | |
| A*26:01 |  | | |  | |  | |  | |  | | | |  | |  | | |  | |  | | | 0.025 | | | |  | | |  | | |  | |  | |  | | | |  | | |  | |  | |  | |
| A*31:01:02 |  | | |  | |  | |  | |  | | | |  | |  | | | 0.030 | |  | | | 0.051 | | | |  | | |  | | |  | |  | |  | | | | 0.017 | | |  | |  | | 0.013 | |
| A*34:01 |  | | |  | |  | |  | |  | | | |  | |  | | | 0.048 | |  | | | 0.136 | | | |  | | |  | | |  | |  | |  | | | | 0.292 | | |  | |  | | 0.090 | |
|  |  | | |  | |  | |  | |  | | | |  | |  | | |  | |  | | |  | | | |  | | |  | | |  | |  | |  | | | |  | | |  | |  | |  | |
| B*07:02 |  | | |  | |  | |  | |  | | | |  | |  | | |  | |  | | |  | | | |  | | |  | | |  | |  | | 0.008 | | | |  | | |  | |  | |  | |
| B*13:01 |  | | |  | |  | |  | |  | | | |  | |  | | | 0.069 | |  | | | 0.191 | | | |  | | |  | | |  | |  | | 0.174 | | | | 0.036 | | |  | |  | |  | |
| B*15:01 |  | | |  | |  | |  | |  | | | |  | |  | | |  | |  | | |  | | | |  | | |  | | |  | |  | | 0.008 | | | |  | | |  | |  | |  | |
| B*15:05 |  | | |  | |  | |  | |  | | | |  | |  | | |  | |  | | |  | | | |  | | |  | | |  | |  | | 0.008 | | | |  | | |  | |  | |  | |
| B*15:06 |  | | |  | |  | |  | |  | | | |  | |  | | | 0.100 | |  | | | 0.191 | | | |  | | |  | | |  | |  | | 0.123 | | | | 0.273 | | |  | |  | |  | |
| B*15:21 |  | | |  | |  | |  | |  | | | |  | |  | | | 0.025 | |  | | |  | | | |  | | |  | | |  | |  | | 0.015 | | | |  | | |  | |  | |  | |
| B*15:25 |  | | |  | |  | |  | |  | | | |  | |  | | |  | |  | | |  | | | |  | | |  | | |  | |  | | 0.015 | | | | 0.082 | | |  | |  | |  | |
| B*15:36 |  | | |  | |  | |  | |  | | | |  | |  | | |  | |  | | | 0.073 | | | |  | | |  | | |  | |  | |  | | | | 0.046 | | |  | |  | |  | |
| B*18:01 |  | | |  | |  | |  | |  | | | |  | |  | | |  | |  | | | 0.009 | | | |  | | |  | | |  | |  | | 0.015 | | | |  | | |  | |  | |  | |
| B*27:04 |  | | |  | |  | |  | |  | | | |  | |  | | | 0.225 | |  | | | 0.055 | | | |  | | |  | | |  | |  | | 0.038 | | | | 0.091 | | |  | |  | |  | |
|  |  | | |  | |  | |  | |  | | | |  | |  | | |  | |  | | |  | | | |  | | |  | | |  | |  | |  | | | |  | | |  | |  | |  | |
| C*01:02 |  | | |  | |  | |  | |  | | | |  | |  | | | 0.200 | |  | | | 0.147 | | | |  | | |  | | |  | |  | | 0.132 | | | |  | | |  | |  | | 0.107 | |
| C*02:02 |  | | |  | |  | |  | |  | | | |  | |  | | |  | |  | | |  | | | |  | | |  | | |  | |  | | 0.007 | | | |  | | |  | |  | |  | |
| C*03:03 |  | | |  | |  | |  | |  | | | |  | |  | | | 0.281 | |  | | |  | | | |  | | |  | | |  | |  | | 0.049 | | | |  | | |  | |  | | 0.018 | |
| C*03:04 |  | | |  | |  | |  | |  | | | |  | |  | | | 0.181 | |  | | | 0.095 | | | |  | | |  | | |  | |  | | 0.083 | | | |  | | |  | |  | | 0.223 | |
| C*04:01 |  | | |  | |  | |  | |  | | | |  | |  | | | 0.069 | |  | | | 0.095 | | | |  | | |  | | |  | |  | | 0.208 | | | |  | | |  | |  | | 0.170 | |
| C*04:03 |  | | |  | |  | |  | |  | | | |  | |  | | | 0.119 | |  | | | 0.250 | | | |  | | |  | | |  | |  | | 0.160 | | | |  | | |  | |  | | 0.080 | |
| C*07:01 |  | | |  | |  | |  | |  | | | |  | |  | | |  | |  | | |  | | | |  | | |  | | |  | |  | | 0.014 | | | |  | | |  | |  | |  | |
| C*07:02 |  | | |  | |  | |  | |  | | | |  | |  | | | 0.019 | |  | | | 0.302 | | | |  | | |  | | |  | |  | | 0.125 | | | |  | | |  | |  | | 0.054 | |
| C*08:01 |  | | |  | |  | |  | |  | | | |  | |  | | |  | |  | | | 0.026 | | | |  | | |  | | |  | |  | | 0.028 | | | |  | | |  | |  | |  | |
| C*12:02 |  | | |  | |  | |  | |  | | | |  | |  | | | 0.031 | |  | | | 0.060 | | | |  | | |  | | |  | |  | | 0.049 | | | |  | | |  | |  | | 0.116 | |
|  |  | | |  | |  | |  | |  | | | |  | |  | | |  | |  | | |  | | | |  | | |  | | |  | |  | |  | | | |  | | |  | |  | |  | |
| DPB1*01:01 |  | | |  | |  | |  | | 0.026 | | | |  | |  | | |  | |  | | |  | | | |  | | |  | | | 0.006 | |  | |  | | | |  | | |  | |  | |  | |
| DPB1*02:01 |  | | |  | |  | |  | | 0.359 | | | |  | |  | | |  | |  | | |  | | | |  | | |  | | | 0.006 | |  | |  | | | |  | | |  | |  | |  | |
| DPB1*03:01 |  | | |  | |  | |  | | 0.039 | | | |  | |  | | |  | |  | | |  | | | |  | | |  | | |  | |  | |  | | | |  | | |  | |  | |  | |
| DPB1*04:01 |  | | |  | |  | |  | | 0.372 | | | |  | |  | | |  | |  | | |  | | | |  | | |  | | | 0.012 | |  | |  | | | |  | | |  | |  | |  | |
| DPB1*05:01 |  | | |  | |  | |  | | 0.160 | | | |  | |  | | |  | |  | | |  | | | |  | | |  | | | 0.976 | |  | |  | | | |  | | |  | |  | |  | |
| DPB1*14:01 |  | | |  | |  | |  | | 0.045 | | | |  | |  | | |  | |  | | |  | | | |  | | |  | | |  | |  | |  | | | |  | | |  | |  | |  | |
|  |  | | |  | |  | |  | |  | | | |  | |  | | |  | |  | | |  | | | |  | | |  | | |  | |  | |  | | | |  | | |  | |  | |  | |
| DQA1*01:01 |  | | |  | | 0.208 | | 0.228 | |  | | | |  | |  | | |  | |  | | | 0.031 | | | |  | | |  | | |  | |  | |  | | | |  | | |  | |  | |  | |
| DQA1*01:02 |  | | |  | | 0.167 | | 0.465 | |  | | | |  | |  | | |  | |  | | | 0.385 | | | |  | | |  | | |  | |  | |  | | | |  | | |  | |  | |  | |
| DQA1*01:03 |  | | |  | | 0.050 | | 0.097 | |  | | | |  | |  | | |  | |  | | | 0.015 | | | |  | | |  | | |  | |  | |  | | | |  | | |  | |  | |  | |
| DQA1*03:01 |  | | |  | | 0.192 | | 0.149 | |  | | | |  | |  | | |  | |  | | | 0.046 | | | |  | | |  | | |  | |  | |  | | | |  | | |  | |  | |  | |
| DQA1*05:01 |  | | |  | | 0.383 | | 0.061 | |  | | | |  | |  | | |  | |  | | | 0.523 | | | |  | | |  | | |  | |  | |  | | | |  | | |  | |  | |  | |
|  |  | | |  | |  | |  | |  | | | |  | |  | | |  | |  | | |  | | | |  | | |  | | |  | |  | |  | | | |  | | |  | |  | |  | |
| DQB1*03:01 |  | | |  | | 0.383 | | 0.061 | |  | | | |  | |  | | |  | | 0.587 | | | 0.523 | | | |  | | |  | | |  | |  | |  | | | |  | | |  | |  | |  | |
| DQB1*03:02 |  | | |  | | 0.092 | |  | |  | | | |  | |  | | |  | |  | | | 0.015 | | | |  | | |  | | |  | |  | |  | | | |  | | |  | |  | |  | |
| DQB1*03:03 |  | | |  | |  | |  | |  | | | |  | |  | | |  | |  | | | 0.008 | | | |  | | |  | | |  | |  | |  | | | |  | | |  | |  | |  | |
| DQB1*04 |  | | |  | |  | |  | |  | | | |  | |  | | |  | | 0.011 | | |  | | | |  | | |  | | |  | |  | |  | | | |  | | |  | |  | |  | |
| DQB1*04:01 |  | | |  | |  | | 0.079 | |  | | | |  | |  | | |  | |  | | | 0.015 | | | |  | | |  | | |  | |  | |  | | | |  | | |  | |  | |  | |
| DQB1*04:02 |  | | |  | | 0.100 | | 0.070 | |  | | | |  | |  | | |  | |  | | | 0.008 | | | |  | | |  | | |  | |  | |  | | | |  | | |  | |  | |  | |
| DQB1*05:01 |  | | |  | |  | |  | |  | | | |  | |  | | |  | | 0.005 | | | 0.008 | | | |  | | |  | | |  | |  | |  | | | |  | | |  | |  | |  | |
| DQB1*05:02 |  | | |  | | 0.167 | | 0.044 | |  | | | |  | |  | | |  | | 0.245 | | | 0.169 | | | |  | | |  | | |  | |  | |  | | | |  | | |  | |  | |  | |
| DQB1*05:03 |  | | |  | | 0.133 | | 0.254 | |  | | | |  | |  | | |  | |  | | | 0.008 | | | |  | | |  | | |  | |  | |  | | | |  | | |  | |  | |  | |
| DQB1*06:01 |  | | |  | | 0.125 | | 0.193 | |  | | | |  | |  | | |  | | 0.141 | | | 0.231 | | | |  | | |  | | |  | |  | |  | | | |  | | |  | |  | |  | |
|  |  | | |  | |  | |  | |  | | | |  | |  | | |  | |  | | |  | | | |  | | |  | | |  | |  | |  | | | |  | | |  | |  | |  | |
| DRB1*04:01 |  | | |  | |  | |  | | 0.011 | | | |  | |  | | |  | |  | | |  | | | |  | | |  | | |  | |  | |  | | | |  | | |  | |  | |  | |
| DRB1*04:03 |  | | |  | | 0.092 | |  | |  | | | |  | |  | | |  | | 0.015 | | | 0.015 | | | |  | | |  | | | 0.123 | |  | |  | | | |  | | |  | |  | |  | |
| DRB1*04:04 |  | | |  | |  | |  | |  | | | |  | |  | | |  | |  | | |  | | | |  | | | 0.013 | | | 0.006 | |  | |  | | | |  | | |  | |  | |  | |
| DRB1*04:05 | 0.222 | | | 0.127 | | 0.092 | | 0.079 | | 0.132 | | | | 0.310 | | 0.192 | | |  | |  | | | 0.023 | | | | 0.200 | | | 0.138 | | | 0.056 | | 0.094 | |  | | | |  | | | 0.245 | | 0.147 | |  | |
| DRB1*04:10 |  | | | 0.008 | | 0.009 | | 0.070 | | 0.029 | | | |  | |  | | |  | |  | | |  | | | |  | | |  | | |  | |  | |  | | | |  | | |  | |  | |  | |
| DRB1*07:01 |  | | |  | |  | |  | | 0.006 | | | |  | |  | | |  | |  | | |  | | | |  | | |  | | |  | |  | |  | | | |  | | |  | |  | |  | |
| DRB1*08:02 |  | | |  | |  | |  | |  | | | | 0.006 | | 0.008 | | |  | |  | | |  | | | | 0.010 | | |  | | |  | | 0.022 | |  | | | |  | | | 0.009 | | 0.039 | |  | |
| DRB1*08:03 | 0.175 | | | 0.254 | | 0.050 | | 0.097 | | 0.063 | | | | 0.209 | | 0.185 | | |  | |  | | | 0.015 | | | | 0.200 | | | 0.208 | | | 0.321 | | 0.167 | |  | | | |  | | | 0.127 | | 0.157 | |  | |
| DRB1*08:10 |  | | |  | |  | |  | |  | | | |  | |  | | |  | |  | | |  | | | |  | | |  | | | 0.006 | |  | |  | | | |  | | |  | |  | |  | |
| DRB1*09:01:02 |  | | |  | |  | |  | | 0.006 | | | |  | |  | | |  | |  | | | 0.008 | | | |  | | |  | | | 0.179 | |  | |  | | | |  | | |  | |  | |  | |

**Supplementary Table 3.** Common HLA alleles and high frequency HLA alleles from endemic regions submitted to NetMHCpan v 4.1 and NetMHCIIpan v 4.0 for the identification of CD8^+^ and CD4^+^ peptides, respectively

| HLA allele gene | HLA allele field |
| --- | --- |
| HLA-alleles selected and used for CD8^+^ peptide identification | |
| HLA-A | 01:01, 01:02, 02:01, 02:02, 02:03, 02:04,02:05, 02:06, 02:07, 02:11, 02:14,03:01, 11:01, 23:01, 24:02, 24:06,24:13, 26:01, 30:01, 30:02, 31:01, 32:01, 33:01, 34:01, 68:01, 68:02 |
| HLA-B | 07:02, 07:05, 07:31, 08:01, 13:01, 13:02, 14:01, 14:02, 14:03, 15:01, 15:02, 15:03, 15:05, 15:06, 15:10, 15:16, 15:21, 15:25, 15:36, 18:01, 27:04, 35:01, 40:01, 44:02, 44:03, 51:01, 53:01, 57:01, 58:01 |
| HLA-C | 01:02, 01:03, 02:02, 03:02, 03:03, 03:04, 03:23, 03:43, 03:64, 04:01, 04:03, 04:07, 05:01, 06:02, 07:01, 07:02, 07:04, 08:01, 12:02 |
| HLA-alleles selected and used for CD4^+^ peptide identification | |
| DRB1 | 01:01, 01:02, 01:03, 03:01, 04:01, 04:02, 04:03, 04:04, 04:05, 04:06, 0407, 04:10, 04:11, 04:12, 07:01, 08:02, 08:03, 08:10, 09:01, 10:01, 11:01, 11:02, 11:07, 12:01, 13:02, 15:01 |
| DRB3 | 01:01, 02:02 |
| DRB4 | 01:01 |
| DRB5 | 01:01 |
| DPA1-DPB1 | DPA10103-DPB10201  DPA10103-DPB10301  DPA10103-DPB10401  DPA10103-DPB10402  DPA10103-DPB10601  DPA10103-DPB11101  DPA10103-DPB11701 |
|  |  |
| DQA1-DQB1 | DQA10102-DQB10501,  DQA10102-DQB10602,  DQA10103-DQB10501,  DQA10103-DQB10603,  DQA10201-DQB10201,  DQA10201-DQB10202,  DQA10501-DQB10201,  DQA10501-DQB10301 |

**Supplementary Table 4.** List of non-allergenic and non-toxin CD8^+^ and CD4^+^ epitopes, their respective alleles, the IC50 and antigenicity scores

| Epitope | HLA alleles | IC50 (nm) | Antigenicity score |
| --- | --- | --- | --- |
| CD8^+^ Epitopes |  |  |  |
| ALRLIRPAW | HLA-A*32:01 | 197.96 | 1.34 |
| APVVGVNPW | HLA-B*53:01 | 92.66 | 1.40 |
| FALRLIRPA | HLA-A*02:06  HLA-C*03:02  HLA-C*03:43 | 63.00  220.98  98.22 | 1.05 |
| FLWGVDGRL | HLA-A*02:01  HLA-A*02:02  HLA-A*02:03  HLA-A*02:04  HLA-A*02:05  HLA-A*02:06  HLA-A*02:11  HLA-A*02:14 | 16.31  12.69  48.27  55.80  124.41  67.57  6.16  109.34 | 1.67 |
| GVDGRLPLL | HLA-A*02:11 | 139.22 | 1.80 |
| GVNPWAITL | HLA-A*02:11  HLA-A*32:01 | 49.66  116.82 | 1.79 |
| IRPAWQRPV | HLA-C*06:02 | 110.54 | 1.03 |
| RLIRPAWQR | HLA-A*03:01  HLA-A*11:01  HLA-A*31:01 | 179.70  141.33  8.88 | 0.66 |
| RYAQRAAHR | HLA-A*31:01 | 14.83 | 0.89 |
| WGVDGRLPL | HLA-C*03:02  HLA-C*03:03  HLA-C*03:04  HLA-C*03:23  HLA-C*03:43  HLA-C*03:64 | 147.46  45.35  45.35  45.35  85.55  45.35 | 0.79 |
| YAQRAAHRL | HLA-C*03:02  HLA-C*03:03  HLA-C*03:04  HLA-C*03:23  HLA-C*03:43  HLA-C*03:64  HLA-C*12:02 | 45.01  12.39  12.39  12.39  29.98  12.39  246.48 | 0.64 |
| CD4^+^ Epitopes |  |  |  |
| GCDSRYAQRAAHRLG | HLA-DRB1*01:01  HLA-DRB5*01:01 | 160.60  170.48 | 1.23 |
| GSAPVVGVNPWAITL | HLA-DRB1*01:01  HLA-DRB1*01:02  HLA-DRB1*07:01  HLA-DRB1*09:01  HLA-DRB1*10:01  HLA-DRB1*13:02 | 107.90  222.51  77.28  141.36  242.18  132.44 | 1.67 |
| IPFALRLIRPAWQRP | HLA-DPA1*01:03-DPB1*03:01  HLA-DQA1*01:02-DQB1*05:01  HLA-DQA1*01:03-DQB1*05:01  HLA-DRB1*01:01  HLA-DRB1*01:02  HLA-DRB1*01:03  HLA-DRB1*04:01  HLA-DRB1*04:02  HLA-DRB1*04:04  HLA-DRB1*04:05  HLA-DRB1*04:10  HLA-DRB1*07:01  HLA-DRB1*08:02  HLA-DRB1*08:10  HLA-DRB1*09:01  HLA-DRB1*10:01  HLA-DRB1*11:01  HLA-DRB1*11:02  HLA-DRB1*12:01  HLA-DRB1*13:02  HLA-DRB1*15:01  HLA-DRB4*01:01  HLA-DRB5*01:01 | 210.64  74.45  111.52  27.64  67.53  197.31  95.10  129.14  39.44  105.20  45.36  179.57  54.65  72.20  155.47  18.51  36.22  42.04  167.97  226.15  108.86  37.36  41.94 | 0.99 |
| LGSAPVVGVNPWAIT | HLA-DQA1*05:01-DQB1*03:01  HLA-DRB1*01:01  HLA-DRB1*07:01  HLA-DRB1*09:01  HLA-DRB1*13:02 | 201.07  181.72  110.43  197.58  221.85 | 1.43 |
| PFALRLIRPAWQRPV | HLA-DPA1*01:03-DPB1*03:01  HLA-DQA1*01:02-DQB1*05:01  HLA-DQA1*01:03-DQB1*05:01  HLA-DRB1*01:01  HLA-DRB1*01:02  HLA-DRB1*01:03  HLA-DRB1*04:01  HLA-DRB1*04:02  HLA-DRB1*04:03  HLA-DRB1*04:04  HLA-DRB1*04:05  HLA-DRB1*04:06  HLA-DRB1*04:10  HLA-DRB1*07:01  HLA-DRB1*08:02  HLA-DRB1*08:10  HLA-DRB1*09:01  HLA-DRB1*10:01  HLA-DRB1*11:01  HLA-DRB1*11:02  HLA-DRB1*12:01  HLA-DRB1*13:02  HLA-DRB1*15:01  HLA-DRB4*01:01  HLA-DRB5*01:01 | 174.56  58.32  84.79  23.61  53.13  170.66  85.74  118.05  240.37  35.75  98.12  240.37  42.49  86.10  53.70  61.84  78.12  17.17  35.93  30.85  119.36  112.29  71.46  33.47  37.44 | 0.96 |
| PGCDSRYAQRAAHRL | HLA-DRB1*01:01  HLA-DRB5*01:01 | 221.61  182.33 | 0.93 |
| WAGFLWGVDGRLPLL | HLA-DQA1*01:02-DQB1*05:01  HLA-DQA1*01:03-DQB1*05:01  HLA-DRB1*01:01  HLA-DRB1*03:01  HLA-DRB1*07:01  HLA-DRB1*09:01  HLA-DRB1*10:01  HLA-DRB3*01:01 | 111.93  236.88  79.84  126.73  214.18  120.45  149.24  128.77 | 0.78 |

The most common HLA alleles for the CD8^+^ and CD4^+^ epitopes used for docking are highlighted.

**Supplementary Table 5.** The virulent MFS transporter proteins of the identified conserved T-cell and B-cell epitopes

| Accession number | Protein name | Source organism | Virulence score |
| --- | --- | --- | --- |
| EUA85481.1 | major facilitator superfamily MFS_1 domain protein | *Mycobacterium ulcerans str. Harvey* | 0.95 |
| EUA85589.1 | major Facilitator Superfamily protein | *Mycobacterium ulcerans str. Harvey* | 1.02 |
| WP_096369848.1 | MFS transporter | *Mycobacterium ulcerans* | 0.69 |
| WP_134429183.1 | multidrug effflux MFS transporter | *Mycobacterium ulcerans* | 1.01 |

**Supplementary Table 6.** The analysis of the number of immune tests performed with the identified T-cell epitopes.

| Epitope | No. of subjects tested/assay performed * | No. of subjects responded/positive assays | Response frequency | Lower bound (95% CI) | Upper bound (95% CI) |
| --- | --- | --- | --- | --- | --- |
| CD8^+^ T-cell epitopes |  |  |  |  |  |
| ALRLIRPAW | 197.76 | 0.28 | 0.00 | 0.00 | 0.02 |
| APVVGVNPW | 92.66 | 0.11 | 0.00 | 0.00 | 0.04 |
| FALRLIRPA | 63.00 | 0.76 | 0.00 | -0.00 | 0.06 |
|  | 220.98 | 0.86 | 0.00 | 0.00 | 0.02 |
|  | 98.22 | 0.74 | 0.00 | 0.00 | 0.04 |
| FLWGVDGRL | 16.31 | 0.23 | 0.00 | 0.00 | 0.16 |
|  | 12.69 | 0.30 | 0.00 | 0.00 | 0.21 |
|  | 48.27 | 1.08 | 0.02 | 0.00 | 0.09 |
|  | 55.80 | 0.21 | 0.00 | 0.00 | 0.07 |
|  | 124.41 | 0.99 | 0.00 | -0.00 | 0.03 |
|  | 67.57 | 0.80 | 0.00 | 0.00 | 0.05 |
|  | 6.16 | 0.34 | 0.00 | 0.00 | 0.38 |
|  | 109.34 | 0.56 | 0.00 | 0.00 | 0.03 |
| GVDGRLPLL | 139.22 | 3.02 | 0.02 | 0.01 | 0.06 |
| GVNPWAITL | 49.66 | 1.77 | 0.04 | 0.00 | 0.09 |
|  | 116.82 | 0.17 | 0.00 | 0.00 | 0.03 |
| IRPAWQRPV | 110.54 | 0.05 | 0.00 | 0.00 | 0.03 |
| RLIRPAWQR | 179.70 | 0.59 | 0.00 | 0.00 | 0.02 |
|  | 141.33 | 0.75 | 0.00 | 0.00 | 0.03 |
|  | 8.88 | 0.04 | 0.00 | 0.00 | 0.30 |
| RYAQRAAHR | 14.83 | 0.11 | 0.00 | 0.00 | 0.18 |
| WGVDGRLPL | 147.46 | 0.67 | 0.00 | 0.00 | 0.03 |
|  | 45.35 | 0.20 | 0.00 | 0.00 | 0.06 |
|  | 45.35 | 0.20 | 0.00 | 0.00 | 0.06 |
|  | 45.35 | 0.20 | 0.00 | 0.00 | 0.06 |
|  | 85.55 | 0.67 | 0.00 | 0.00 | 0.04 |
|  | 45.35 | 0.20 | 0.00 | 0.00 | 0.06 |
| YAQRAAHRL | 45.01 | 0.26 | 0.00 | 0.00 | 0.06 |
|  | 12.39 | 0.05 | 0.00 | 0.00 | 0.21 |
|  | 12.39 | 0.05 | 0.00 | 0.00 | 0.21 |
|  | 12.39 | 0.05 | 0.00 | 0.00 | 0.21 |
|  | 29.98 | 0.29 | 0.00 | 0.00 | 0.09 |
|  | 12.39 | 0.05 | 0.00 | 0.00 | 0.21 |
|  | 246.48 | 0.31 | 0.00 | 0.00 | 0.02 |
|  |  |  |  |  |  |
| CD4^+^ T-cell epitopes |  |  |  |  |  |
| GCDSRYAQRAAHRLG | 160.60 | 16.98 | 0.11 | 0.06 | 0.16 |
|  | 170.48 | 6.76 | 0.04 | 0.02 | 0.07 |
| GSAPVVGVNPWAITL | 107.90 | 12.50 | 0.12 | 0.07 | 0.19 |
|  | 222.51 | 6.95 | 0.03 | 0.01 | 0.06 |
|  | 77.28 | 2.89 | 0.04 | 0.01 | 0.09 |
|  | 141.36 | 4.71 | 0.03 | 0.01 | 0.07 |
|  | 242.18 | 23.24 | 0.10 | 0.06 | 0.14 |
|  | 132.44 | 4.22 | 0.03 | 0.01 | 0.08 |
| IPFALRLIRPAWQRP | 210.64 | 1.14 | 0.01 | 0.00 | 0.03 |
|  | 74.45 | 4.53 | 0.06 | 0.02 | 0.13 |
|  | 111.52 | 1.70 | 0.02 | 0.00 | 0.05 |
|  | 27.64 | 3.20 | 0.12 | 0.03 | 0.26 |
|  | 67.53 | 1.46 | 0.02 | 0.00 | 0.08 |
|  | 197.31 | 0.69 | 0.00 | 0.00 | 0.02 |
|  | 95.10 | 1.39 | 0.01 | 0.00 | 0.06 |
|  | 129.14 | 0.08 | 0.00 | 0.00 | 0.03 |
|  | 39.44 | 0.37 | 0.00 | 0.00 | 0.07 |
|  | 105.20 | 1.66 | 0.02 | 0.00 | 0.05 |
|  | 45.36 | 0.15 | 0.00 | 0.00 | 0.06 |
|  | 179.57 | 6.92 | 0.04 | 0.02 | 0.07 |
|  | 54.65 | 0.06 | 0.00 | 0.00 | 0.07 |
|  | 72.20 | 0.10 | 0.00 | 0.00 | 0.05 |
|  | 155.47 | 5.32 | 0.03 | 0.01 | 0.07 |
|  | 18.51 | 0.85 | 0.00 | 0.00 | 0.14 |
|  | 36.22 | 0.63 | 0.00 | 0.00 | 0.08 |
|  | 42.04 | 0.97 | 0.00 | 0.00 | 0.07 |
|  | 167.97 | 1.09 | 0.01 | 0.00 | 0.03 |
|  | 226.15 | 7.16 | 0.03 | 0.02 | 0.06 |
|  | 108.86 | 3.07 | 0.03 | 0.01 | 0.08 |
|  | 37.36 | 0.08 | 0.00 | 0.00 | 0.07 |
|  | 41.94 | 1.36 | 0.03 | 0.00 | 0.11 |
| LGSAPVVGVNPWAIT | 201.07 | 6.73 | 0.03 | 0.01 | 0.06 |
|  | 181.72 | 18.55 | 0.10 | 0.06 | 0.15 |
|  | 110.43 | 4.21 | 0.04 | 0.01 | 0.09 |
|  | 197.58 | 7.14 | 0.04 | 0.02 | 0.07 |
|  | 221.85 | 7.03 | 0.03 | 0.02 | 0.06 |
| PFALRLIRPAWQRPV | 174.56 | 0.87 | 0.00 | 0.00 | 0.02 |
|  | 58.32 | 2.48 | 0.04 | 0.01 | 0.12 |
|  | 84.79 | 0.76 | 0.00 | 0.00 | 0.04 |
|  | 23.61 | 2.64 | 0.11 | 0.02 | 0.24 |
|  | 53.13 | 0.98 | 0.00 | -0.00 | 0.07 |
|  | 170.66 | 0.55 | 0.00 | 0.00 | 0.02 |
|  | 85.74 | 1.16 | 0.01 | 0.00 | 0.06 |
|  | 118.05 | 0.07 | 0.00 | -0.00 | 0.03 |
|  | 240.37 | 0.09 | 0.00 | -0.00 | 0.02 |
|  | 35.75 | 0.29 | 0.00 | 0.00 | 0.08 |
|  | 98.12 | 1.49 | 0.02 | 0.00 | 0.06 |
|  | 240.37 | 0.09 | 0.00 | -0.00 | 0.02 |
|  | 42.49 | 0.12 | 0.00 | 0.00 | 0.07 |
|  | 86.10 | 3.25 | 0.04 | 0.01 | 0.10 |
|  | 53.70 | 0.06 | 0.00 | -0.00 | 0.07 |
|  | 61.84 | 0.04 | 0.00 | 0.00 | 0.06 |
|  | 78.12 | 1.99 | 0.03 | 0.00 | 0.07 |
|  | 17.17 | 0.73 | 0.00 | 0.00 | 0.15 |
|  | 35.93 | 0.62 | 0.00 | 0.00 | 0.08 |
|  | 30.85 | 0.52 | 0.00 | 0.00 | 0.09 |
|  | 119.36 | 0.59 | 0.00 | 0.00 | 0.03 |
|  | 112.29 | 3.52 | 0.03 | 0.01 | 0.08 |
|  | 71.46 | 1.71 | 0.02 | 0.00 | 0.08 |
|  | 33.47 | 0.05 | 0.00 | 0.00 | 0.08 |
|  | 37.44 | 1.17 | 0.03 | 0.00 | 0.12 |
| PGCDSRYAQRAAHRL | 221.61 | 21.21 | 0.10 | 0.06 | 0.14 |
|  | 182.33 | 7.22 | 0.04 | 0.02 | 0.08 |
| WAGFLWGVDGRLPLL | 111.93 | 9.56 | 0.09 | 0.04 | 0.15 |
|  | 236.88 | 8.47 | 0.04 | 0.02 | 0.07 |
|  | 79.84 | 9.68 | 0.12 | 0.06 | 0.20 |
|  | 126.73 | 1.25 | 0.01 | 0.00 | 0.04 |
|  | 214.18 | 8.29 | 0.04 | 0.02 | 0.07 |
|  | 120.45 | 3.81 | 0.03 | 0.01 | 0.07 |
|  | 149.24 | 15.78 | 0.11 | 0.06 | 0.16 |
|  | 128.77 | 1.68 | 0.01 | 0.00 | 0.04 |

*The IC_50_ values correspond with the number of subjects tested/assay performed.

**Supplementary Table 7.** The individual and combined population coverage of the CD8^+^ and CD4^+^ T-cell epitopes globally and for identified endemic regions

| Population/region | Class I | | | Class II | | | Class combined | | |
| --- | --- | --- | --- | --- | --- | --- | --- | --- | --- |
|  | Coverage (%) | Average hits | pc90 | Coverage (%) | Average hits | pc90 | Coverage (%) | Average hits | pc90 |
| World | 83.15 | 1.71 | 0.59 | 99.40 | 7.82 | 3.84 | 99.90 | 9.53 | 5.25 |
| Australia | 67.81 | 1.16 | 0.31 | 89.49 | 3.73 | 0.95 | 96.62 | 4.89 | 1.91 |
| Cameroon | 68.50 | 1.17 | 0.32 | 98.21 | 6.47 | 3.13 | 99.44 | 7.63 | 3.99 |
| Central African Republic | 16.57 | 0.17 | 0.12 | 93.24 | 5.01 | 1.50 | 94.36 | 5.18 | 1.67 |
| Congo | 0.00 | 0.00 | 0.00 | 98.8 | 7.45 | 3.64 | 98.80 | 7.45 | 3.64 |
| Côte d'Ivoire | 40.18 | 0.40 | 0.17 | 0.00 | 0.00 | 0.00 | 40.18 | 0.40 | 0.17 |
| Gabon | 0.00 | 0.00 | 0.00 | 99.43 | 7.40 | 3.87 | 99.43 | 7.40 | 3.87 |
| Japan | 84.52 | 2.23 | 0.65 | 98.21 | 7.18 | 3.14 | 99.72 | 9.41 | 5.04 |
| Liberia | 0.00 | 0.00 | 0.00 | 21.81 | 0.45 | 0.26 | 21.81 | 0.45 | 0.26 |
| Nigeria | 0.00 | 0.00 | 0.00 | 43.64 | 0.90 | 0.35 | 43.64 | 0.90 | 0.35 |
| Papua New Guinea | 77.87 | 1.56 | 0.45 | 99.94 | 7.01 | 4.47 | 99.99 | 8.58 | 5.67 |
| Sudan | 84.62 | 1.64 | 0.65 | 92.21 | 3.95 | 1.18 | 98.80 | 5.58 | 2.50 |
| West Africa | 70.30 | 1.42 | 0.34 | 99.84 | 8.51 | 4.54 | 99.95 | 9.92 | 5.68 |
| Average | 45.66 | 0.88 | 0.28 | 79.56 | 5.07 | 2.37 | 84.05 | 5.95 | 3.08 |
| Standard deviation | 35.42 | 0.78 | 0.24 | 32.91 | 2.88 | 1.63 | 27.19 | 3.32 | 1.99 |

pc90 = minimum number of epitope hits and HLA combinations that are recognized by 90% of the population.

**(A)**
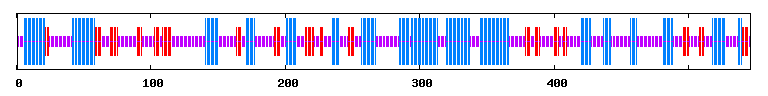


**(B)**
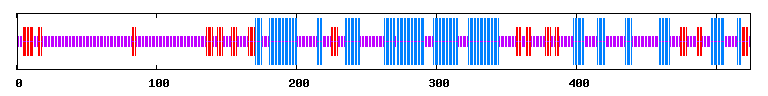


**Supplementary Figure 1.** The secondary structures for the candidate vaccine construct, with the alpha helices shown in blue, the extended strands in red, and the random coils in yellow. **(A)** The secondary structures graph generated for vaccine construct one. **(B)** The secondary structures graph generated for vaccine construct two.

**(A)**


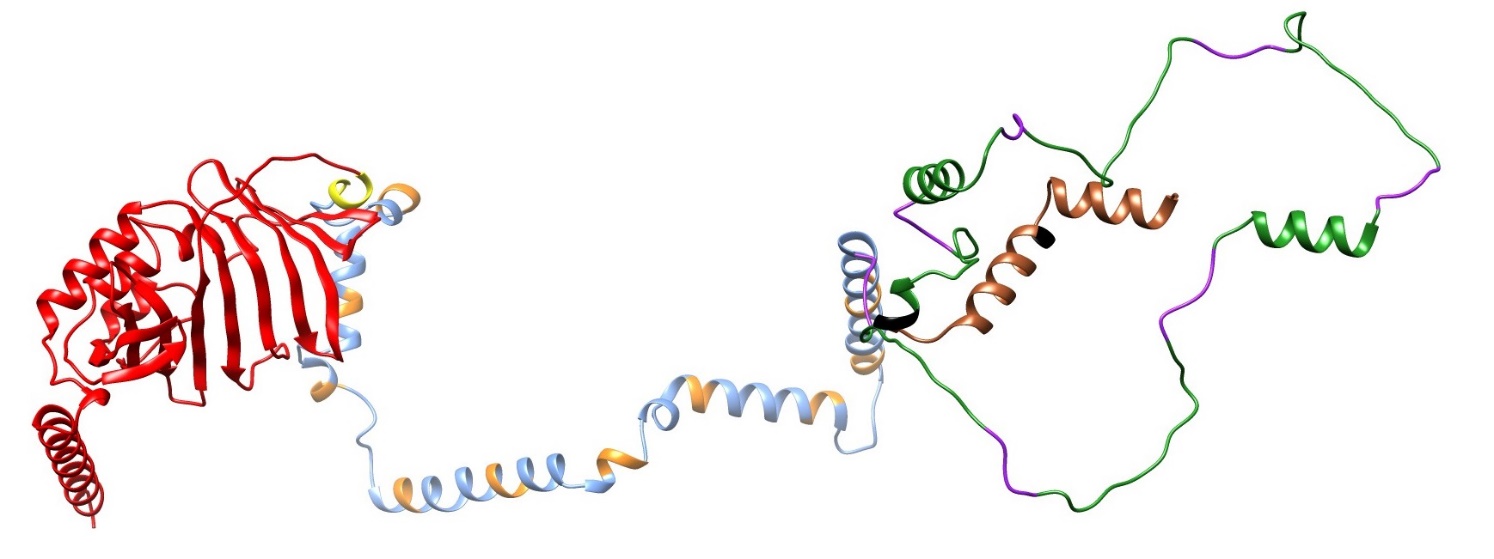


**(B)**


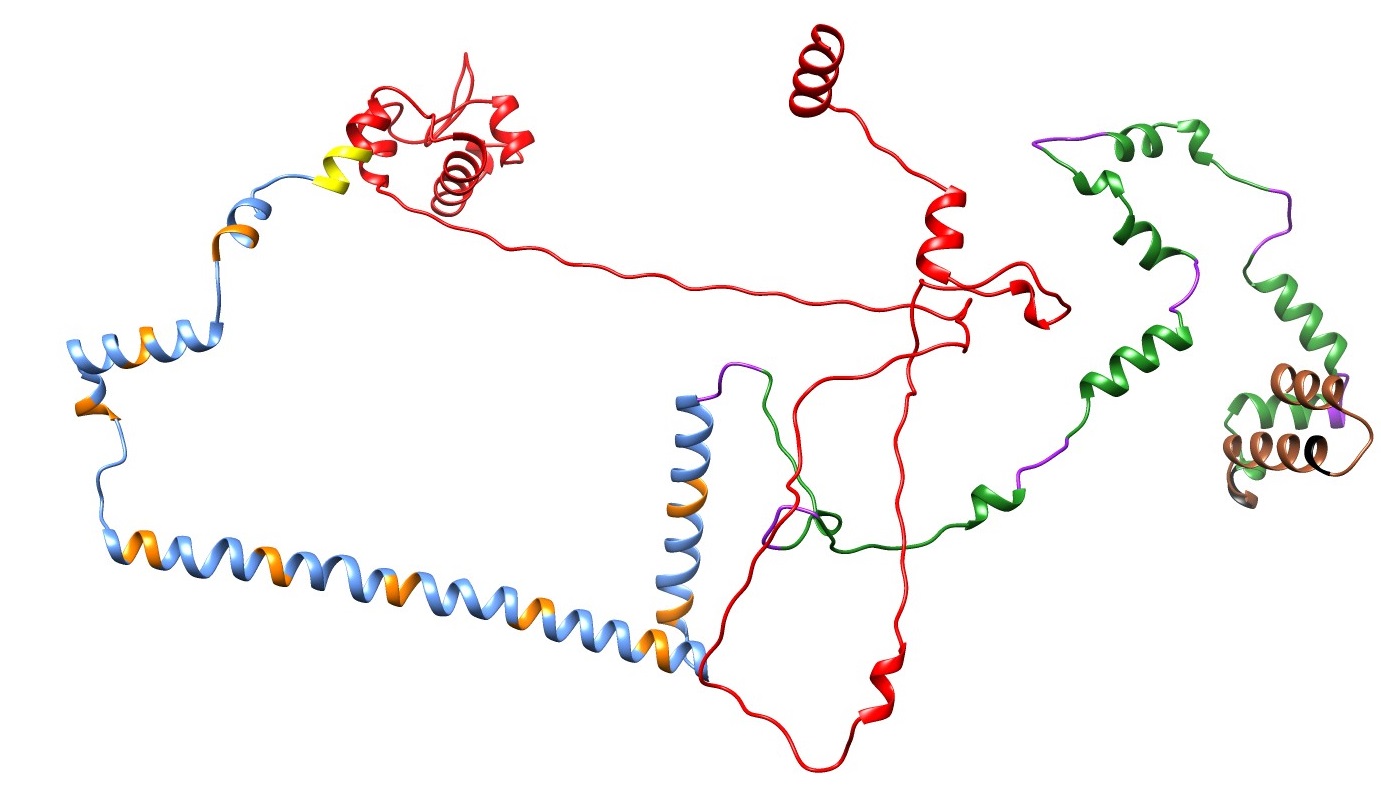


**Supplementary Figure 2.** The refined 3D model for the multi-epitope vaccines. The structures consist of the respective adjuvants (red), EAAAK linker (yellow), CD8^+^ T-cell epitopes (blue), AAY linkers (orange), CD4^+^ T-cell epitopes (green), GPGPG linkers (purple), KK linkers (black) and the B-cell epitope (brown). (A) The refined model of construct 1. (B) The refined model of construct 2.

**(A)**

**(B)**
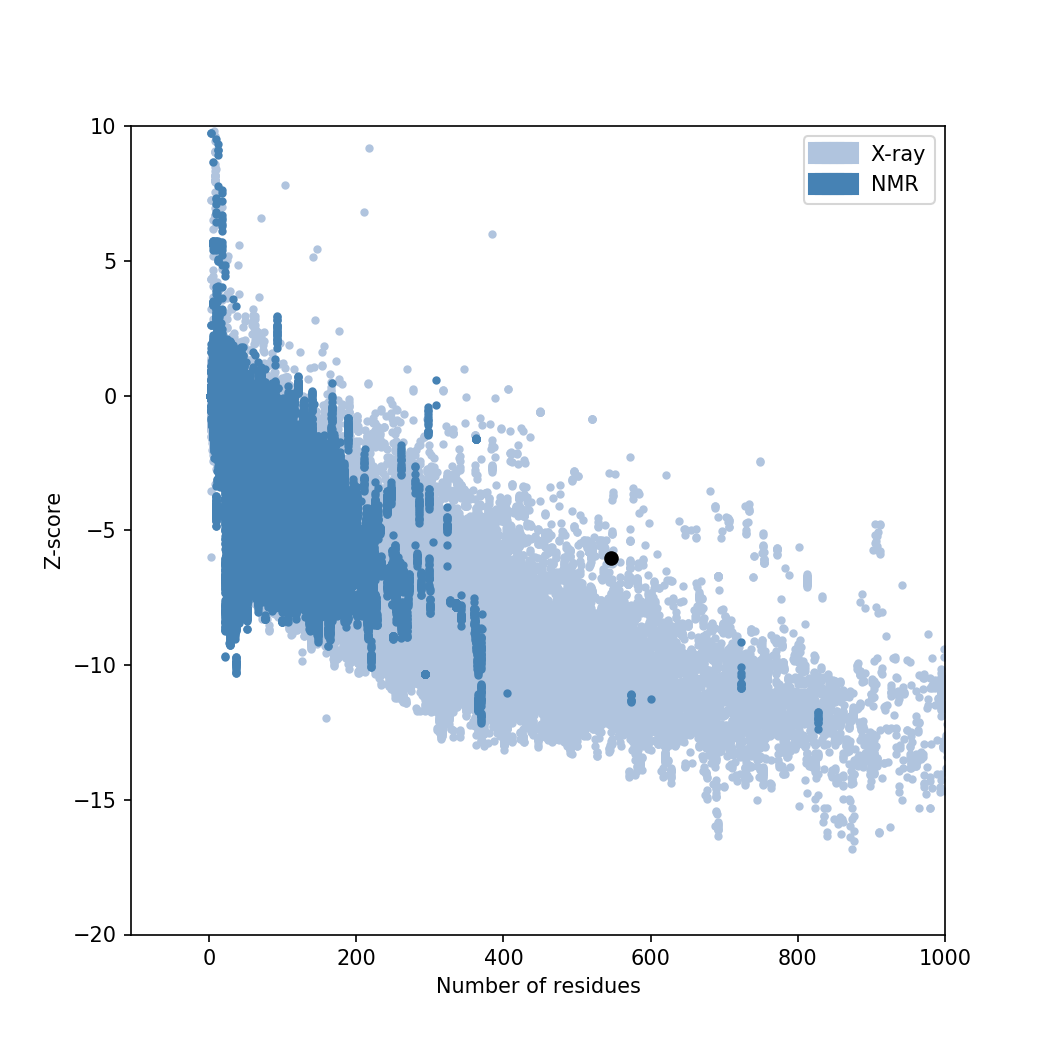

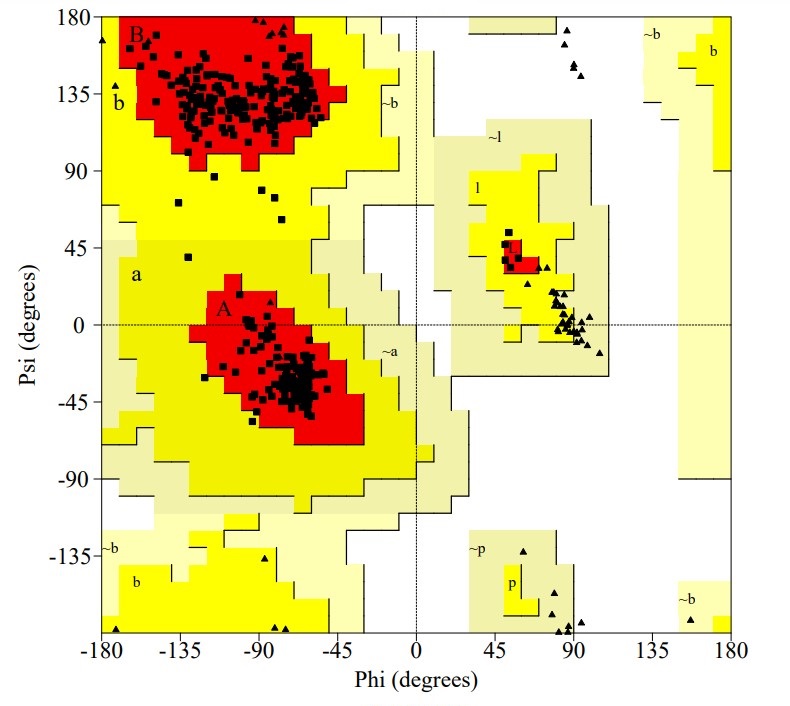


**(C)**


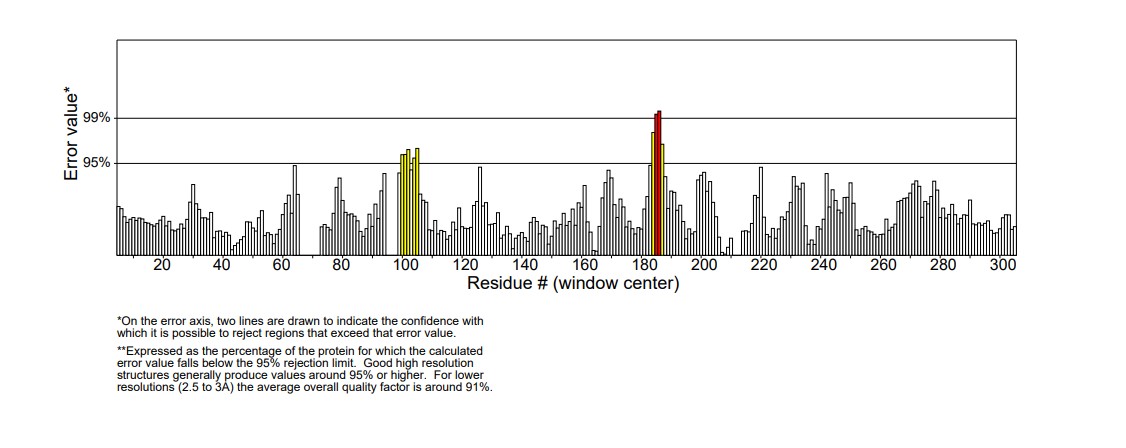


**Supplementary Figure 3.** Various analyses showing vaccine modelling after refinement and validation for vaccine construct one. **(A)** Validation of the refined vaccine model using ProSA. **(B)** Ramachandran plot generated for refined model of vaccine construct one. **(C)** The ERRAT score was generated for vaccine model one.

**(A)**


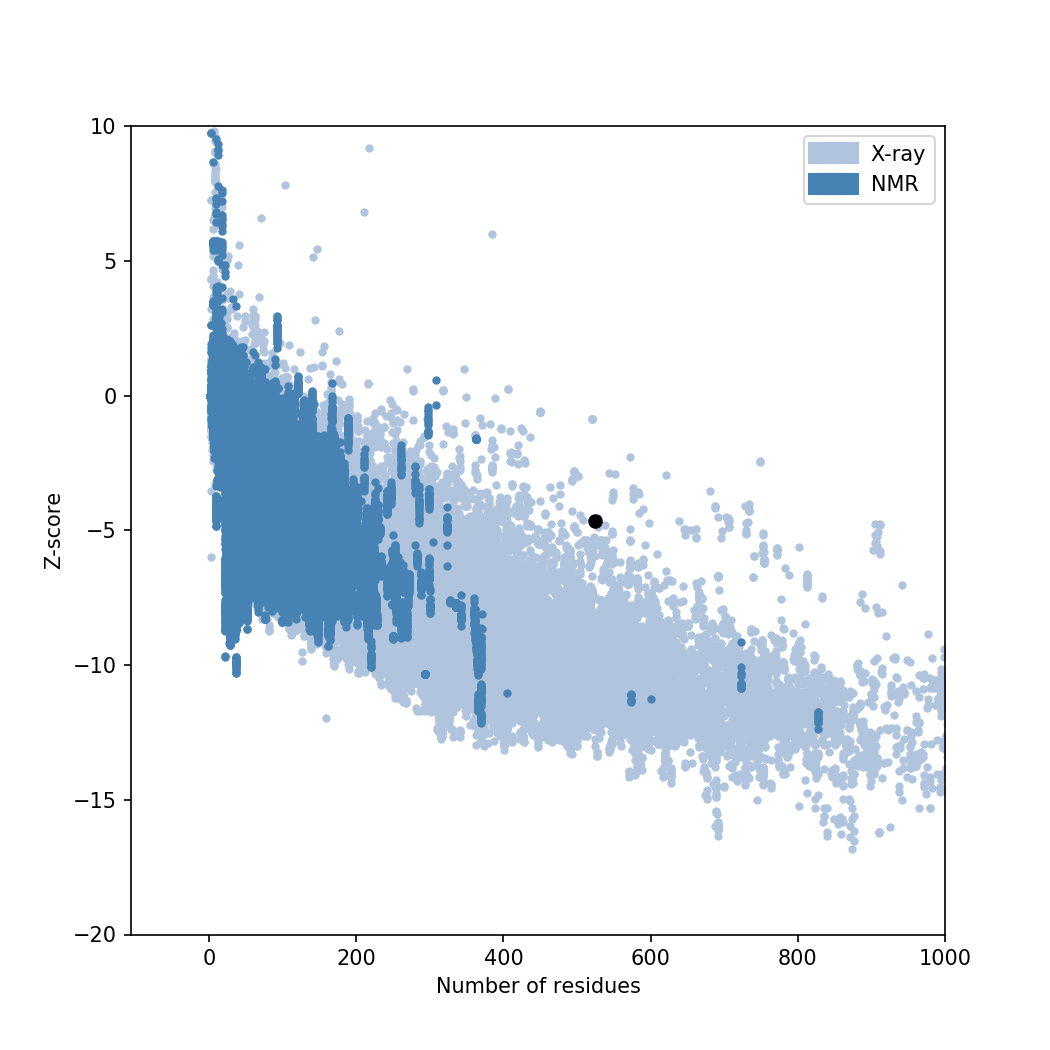


**(B)**


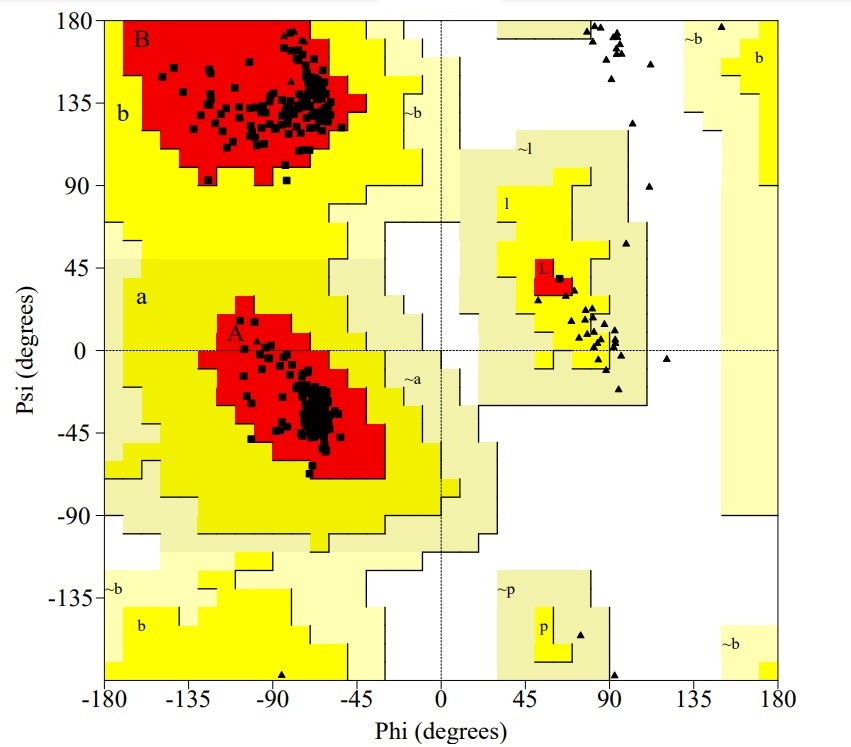


**(C)**


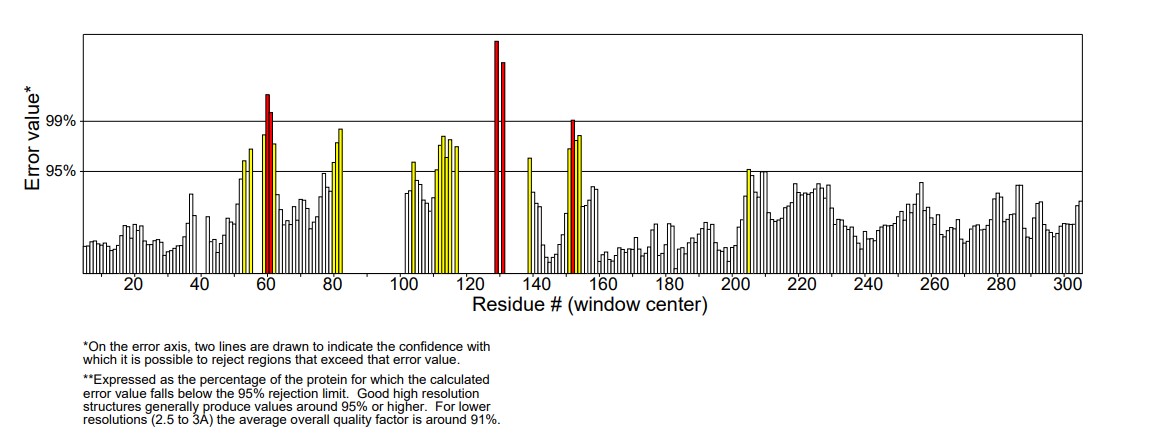


**Supplementary Figure 4.** Various analyses showing vaccine modelling after refinement and validation for vaccine construct two. **(A)** Validation of the refined vaccine model using ProSA. **(B)** Ramachandran plot generated for refined model of vaccine construct two. **(C)** The ERRAT score was generated for vaccine model two.

**(A)**


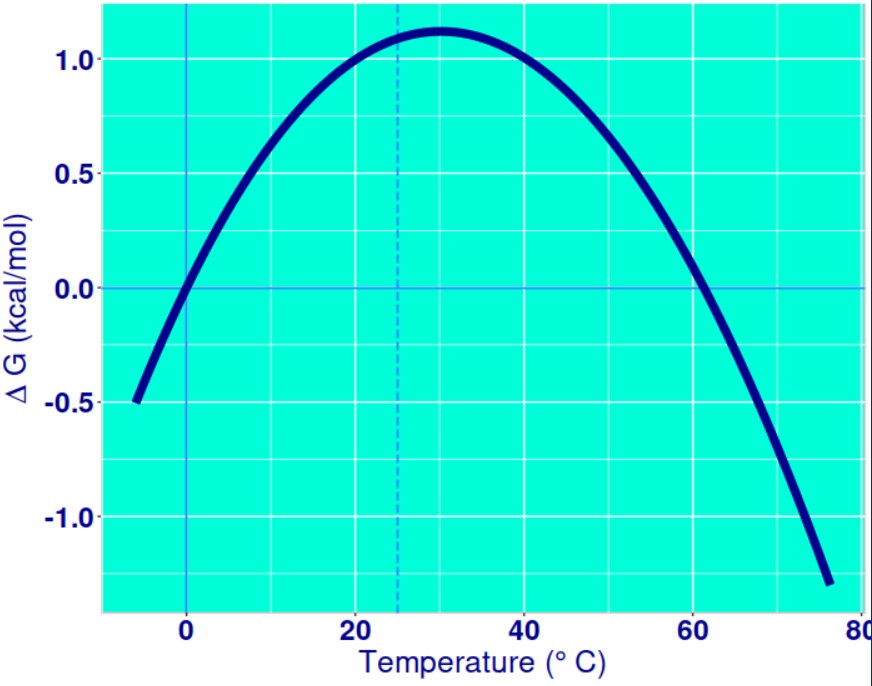


**(B)**


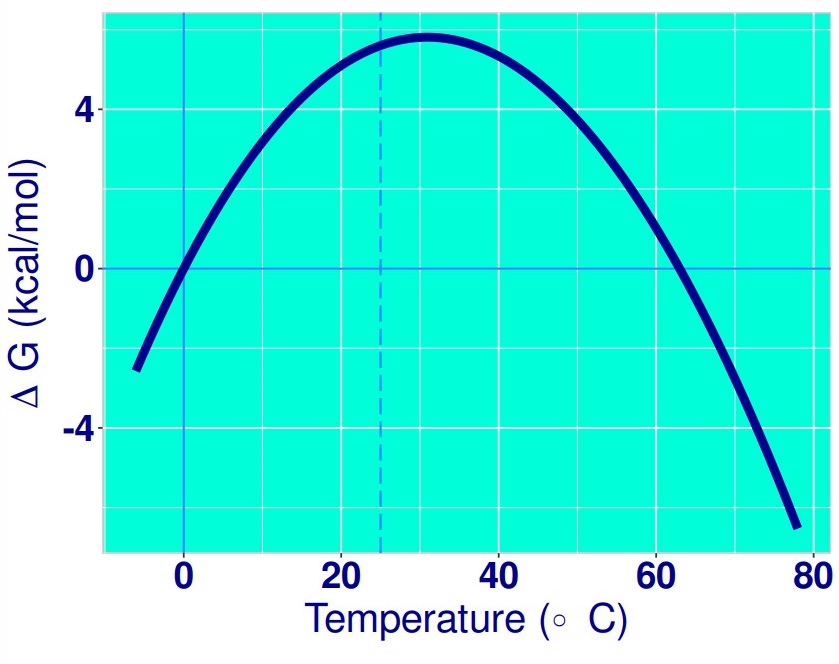


**Supplementary Figure 5.** The Gibbs-Helmholtz curve based on the two refined model candidate structures. **(A)** The Gibbs-Helmholtz curve based on the refined vaccine construct one. **(B)** The Gibbs-Helmholtz curve based on the refined vaccine construct two.

**(A)**


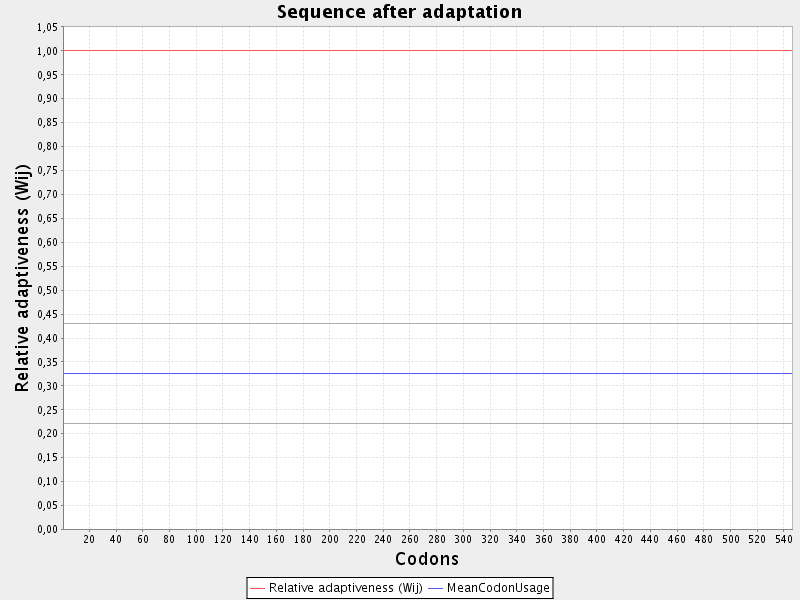


**(B)**


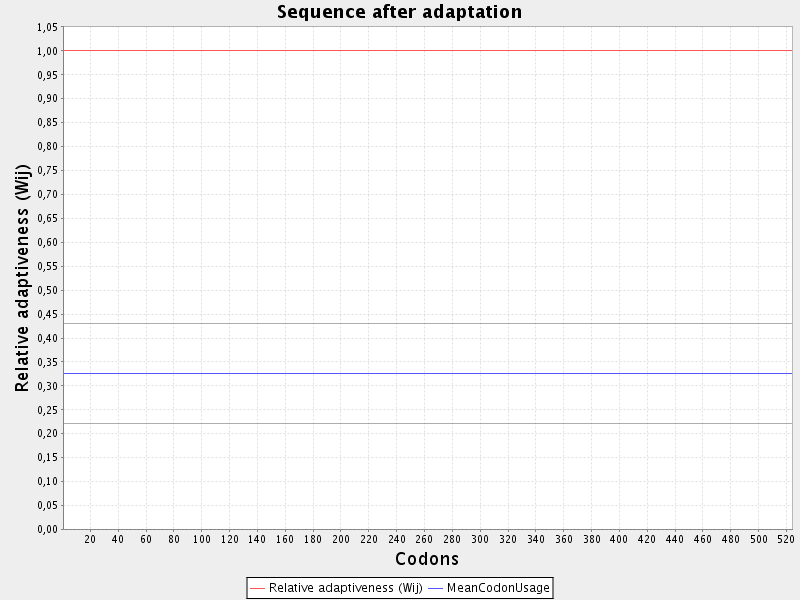


**Supplementary Figure 6.** The relative adaptiveness values for the codons of the two vaccine constructs. **(A)** The relative adaptiveness graph for vaccine construct one. **(B)** The relative adaptiveness graph for vaccine construct two.

**(A)**


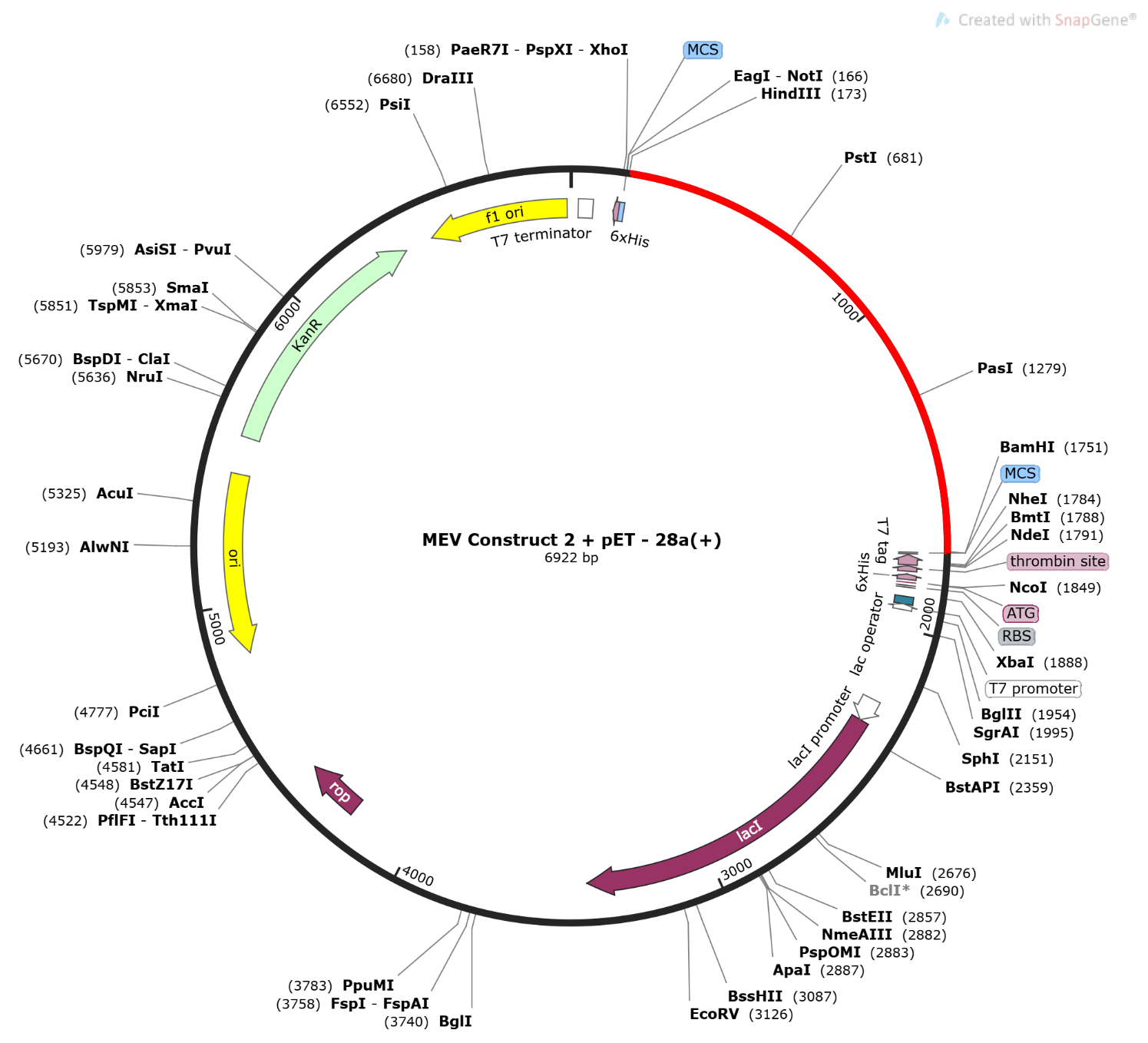


**Supplementary Figure 7.** The *in-silico* cloning map of the pET-28a(+) plasmid, with the optimized DNA sequence of the second MEV construct shown in red. The sequence is located between HindIII (173) and BamHI (1751).

**(A)**


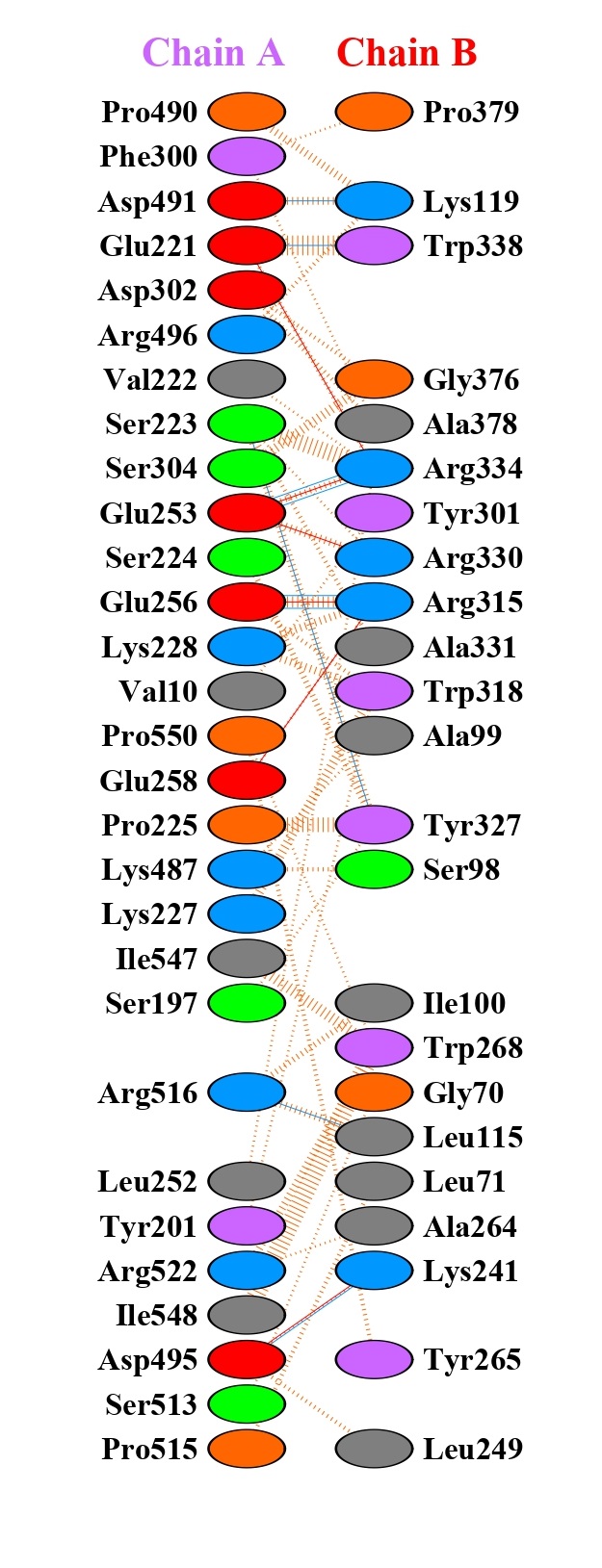


**(B)**


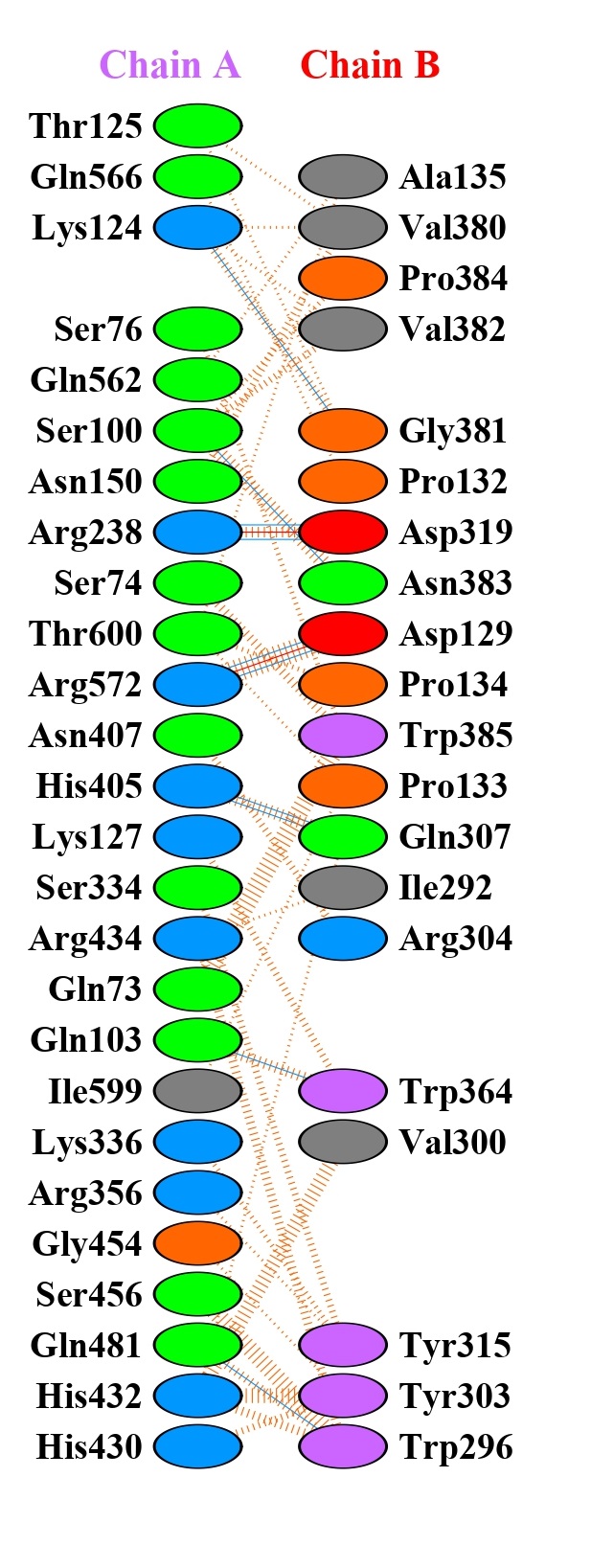


**Supplementary Figure 8.** Diagram indicating the interactions between the amino acid residues of the chains of complex one and two. The hydrogen bonds are indicated in blue, salt bridges in red and other types of contact are shown in orange. The residues are colour coded based on their properties, with positive residues shown in blue, negative in red, neutral in green, aliphatic in grey, aromatic in pink, proline and glycine shown in orange and cysteine shown in yellow. (A) Diagram indicating the bonds between the first vaccine construct and TLR2. (B) Diagram indicating the bonds between the second vaccine construct and TLR4.

**(A)**


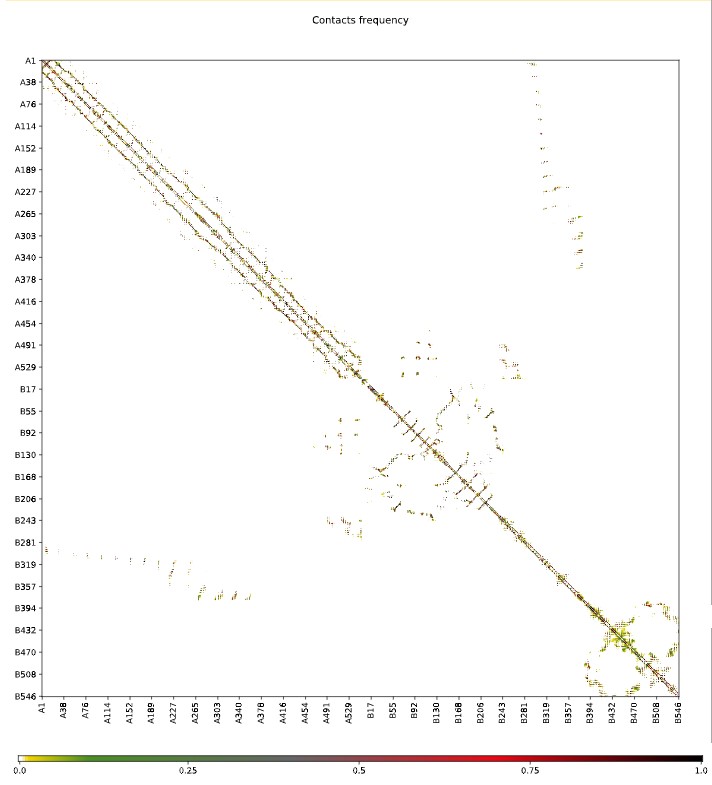


**(B)**


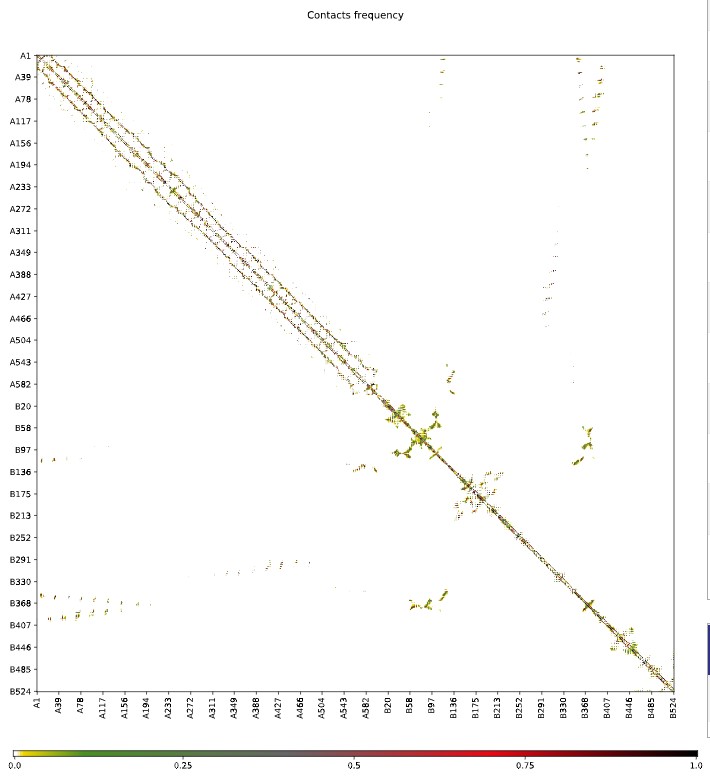


**Supplementary Figure 9.** The structural flexibility of the docked complexes. The range of the frequency of occurrence of the interaction between all atoms is shown, ranging from yellow to red, in increasing frequency. **(A)** The contact map based on the MEV-TLR2 complex. **(B)** The contact map based on the MEV-TLR4 complex.

**(A)**


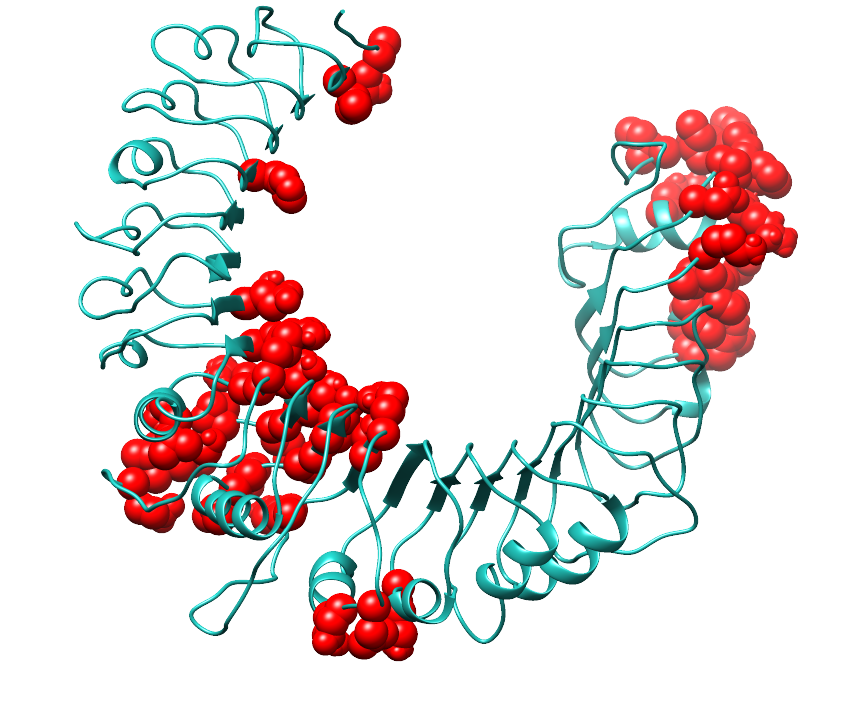


**(B)**


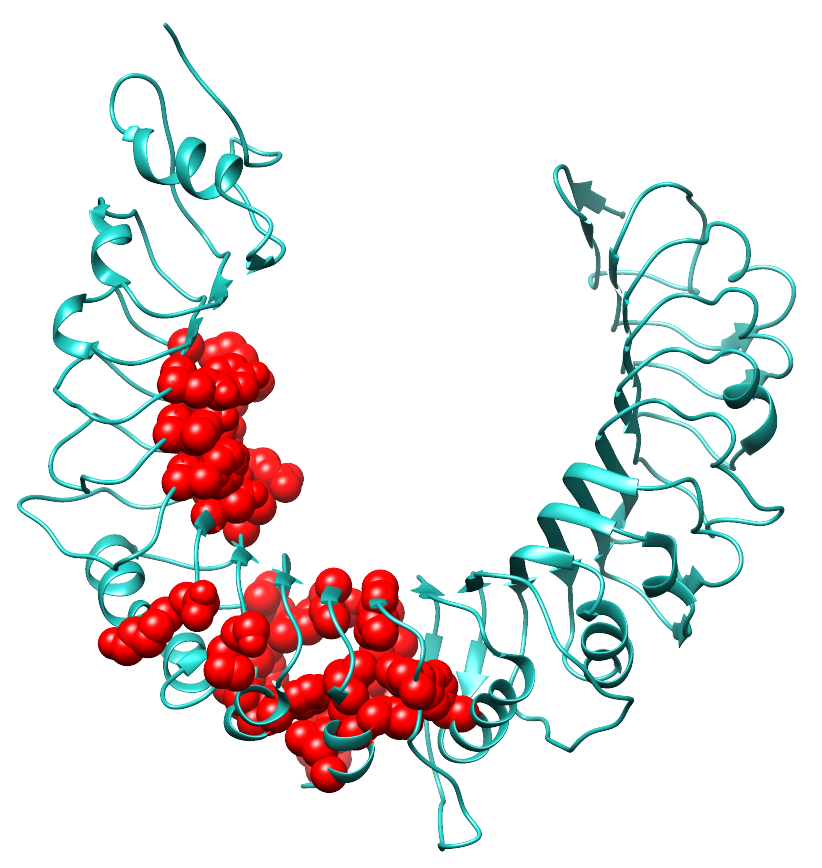


**Supplementary Figure 10.** The mapped binding domains of ligands to TLR2. The binding domains are indicated in red and the TLR chain is shown in blue. (A) The mapped binding domain of the first vaccine construct to TLR2. (B) The mapped binding domain of *Streptococcus Pneumoniae* lipoteichoic acid to TLR2.

**(A)**


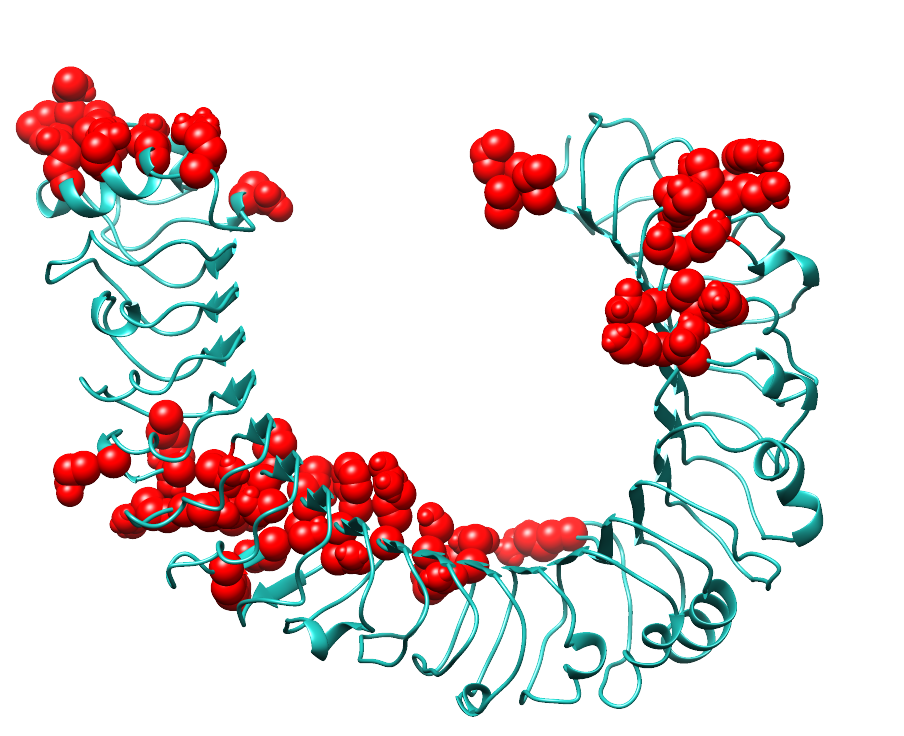


**(B)**


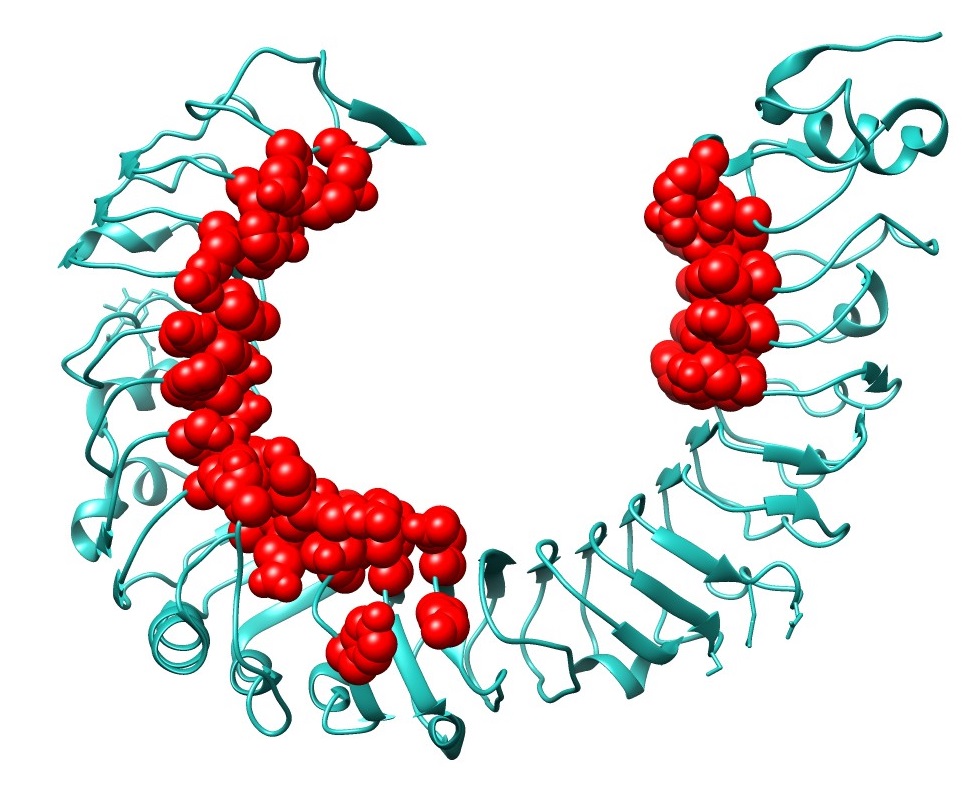


**Supplementary Figure 11.** The mapped binding domains of ligands to TLR4. The binding domains are indicated in red and the TLR chain is shown in blue. (A) The mapped binding domain of the second vaccine construct to TLR4. (B) The mapped binding domain of the MD-2 and LPS ligands to TLR4.

**(A)**


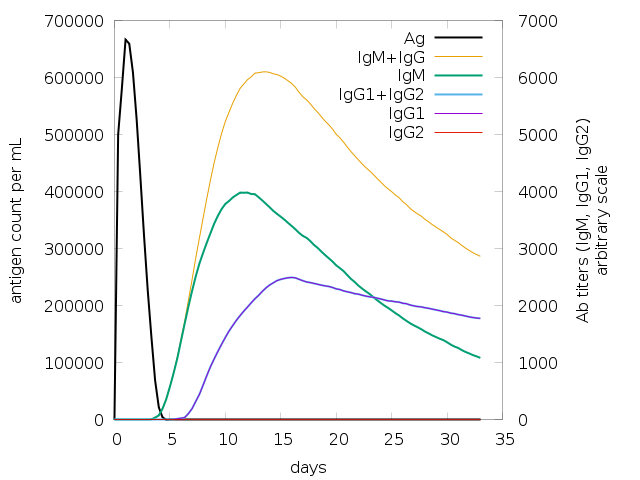


**(B)**


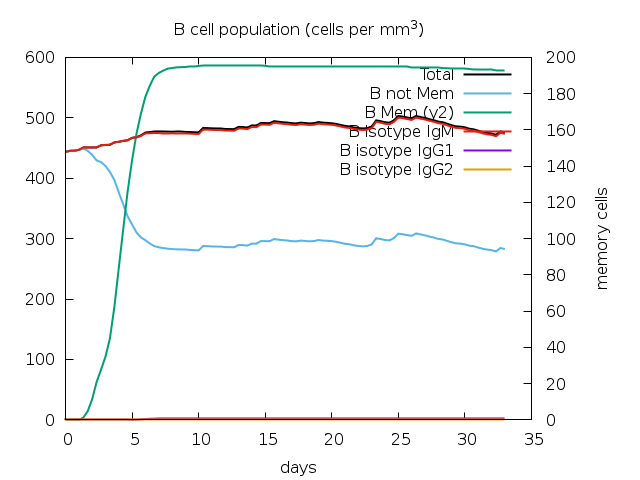


**(C)**


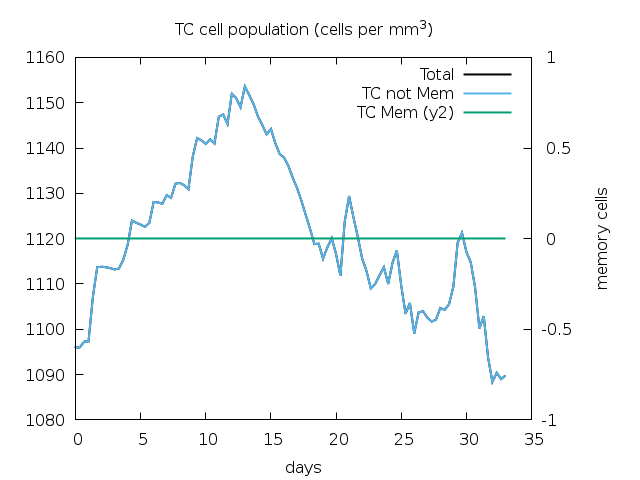


**(D)**


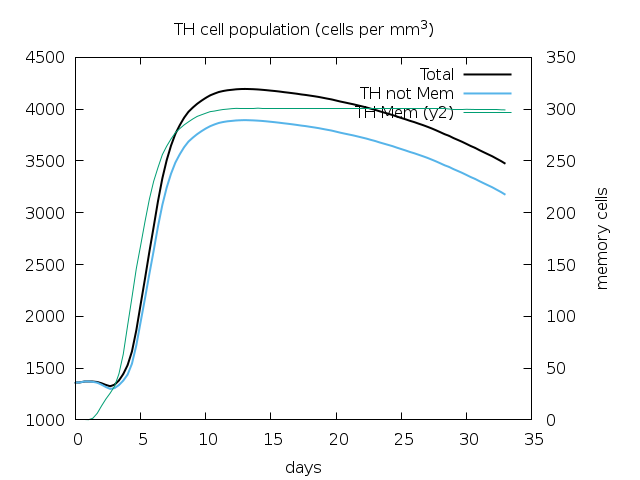


**(E)**


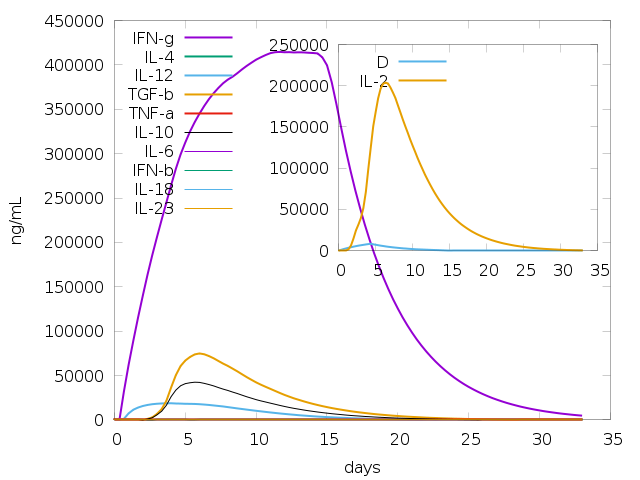


**Supplementary Figure 12.** The immune simulation results from C-IMMSIM of MEV construct two. (A) The induced antigen and immunoglobin responses. (B) The B-cell population: total count, memory cells, and sub-divided into isotypes IgM, IgG1 and IgG2 (C) The CD8^+^ cytotoxic lymphocytes count. (D) The CD4^+^ T-helper lymphocyte count. (E) The induced cytokine response. The inset plot displays the danger signal coupled with IL-2.

**(A)**


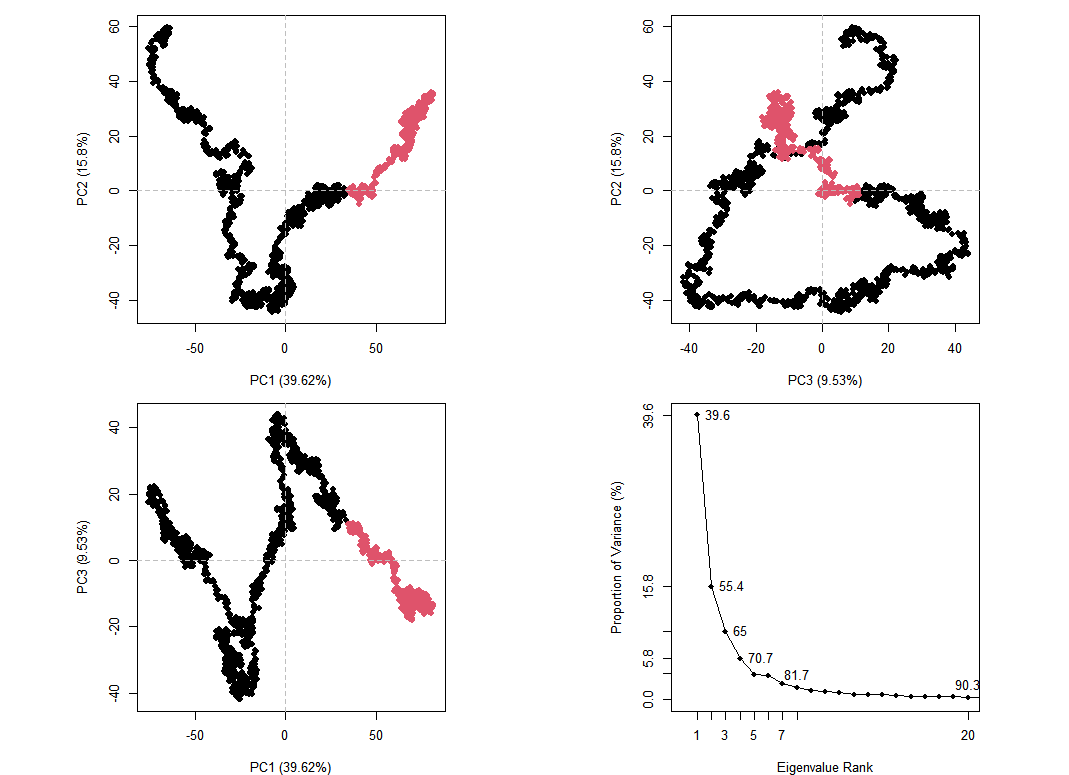


**(B)**


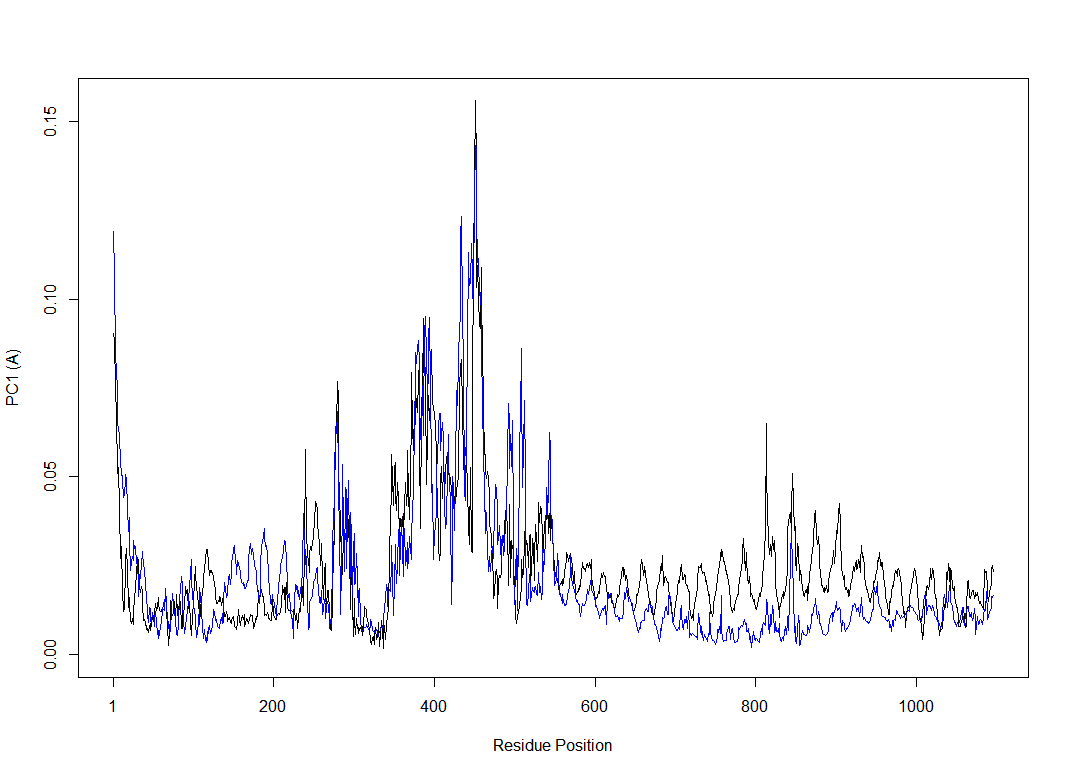


**(C)**


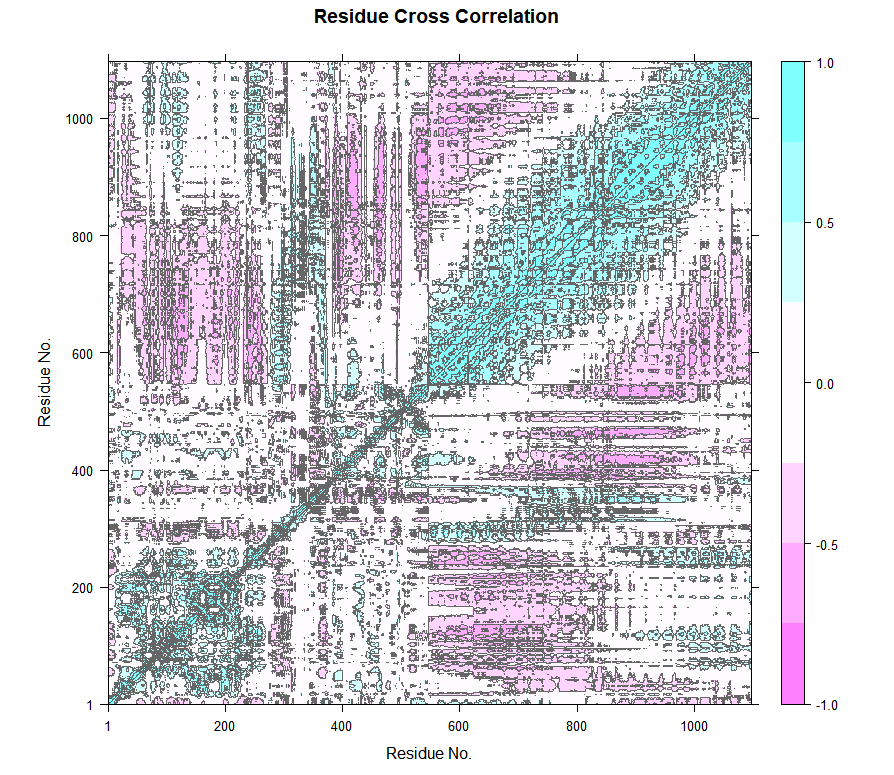


**Supplementary Figure 13.** Post MDS analysis of the MEV-TLR2 docked complex. **(A)** The PCA plots for MEV-TLR2 complex in eigenvalue rank; PC2 vs PC1, PC2 vs PC3, PC3 vs PC1. The colours are based on order of time and the cumulative variability at each data point. **(B)** The residue-wise loadings indicating PC1 and PC2 based on the MEV-TLR2 complex. The graphs are shown in black and blue, respectively. **(C)** The dynamical cross-correlation map generated based on the MEV-TLR2 complex. The blue regions indicate the residues moving in a singular direction, while the pink regions indicate that the residues moved in opposite directions.

**(A)**


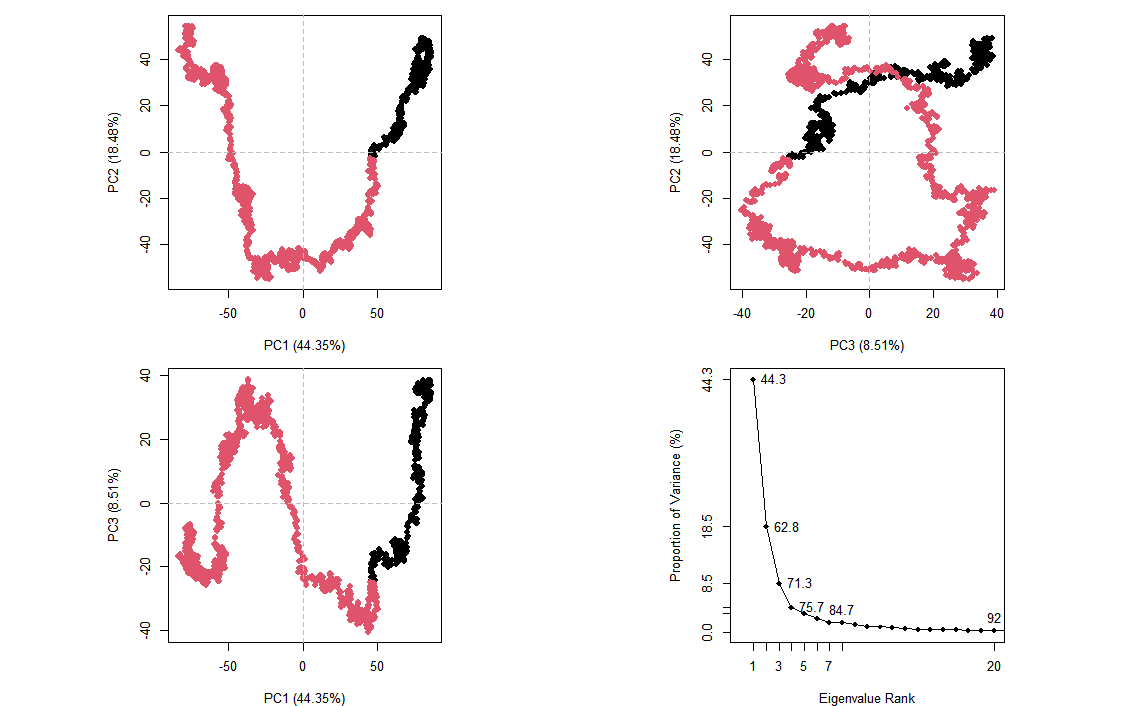


**(B)**


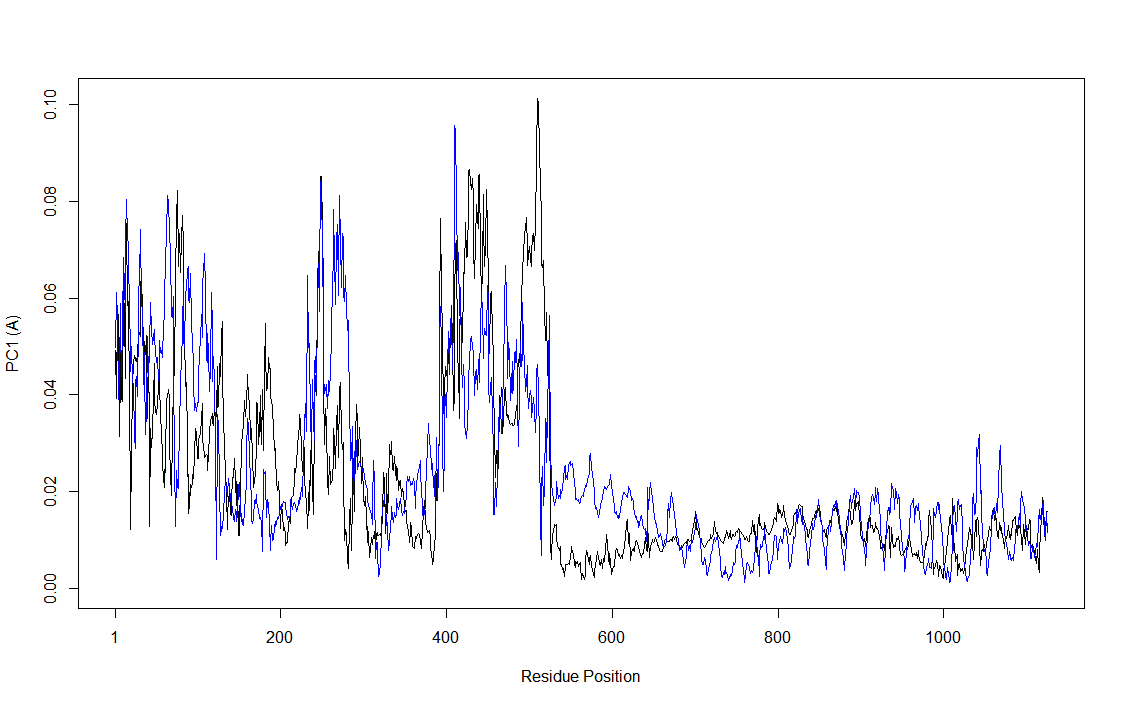


**(C)**


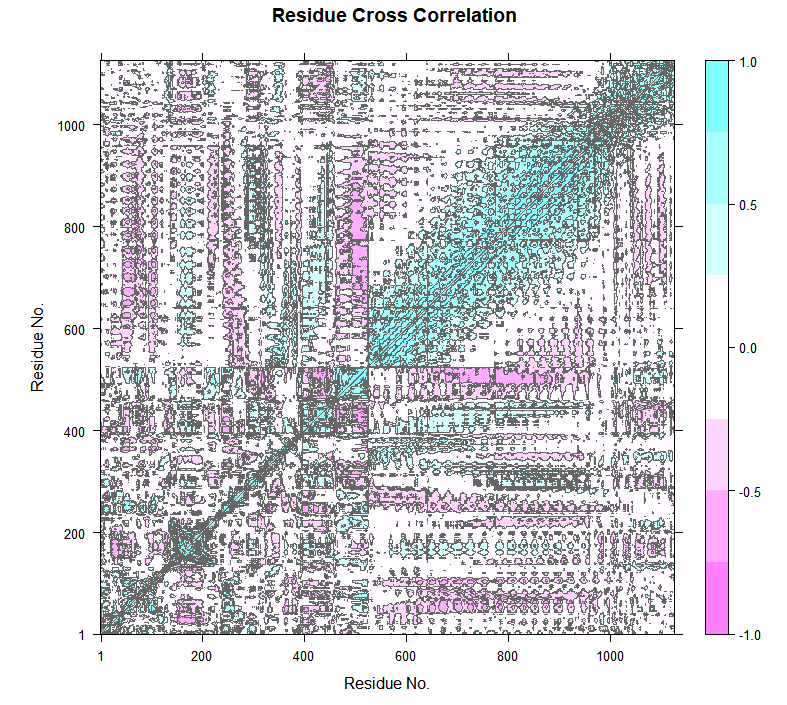


**Supplementary Figure 14.** Post MDS analysis of the MEV-TLR4 docked complex. **(A)** The PCA plots for MEV-TLR4 complex in eigenvalue rank; PC2 vs PC1, PC2 vs PC3, PC3 vs PC1. The colours are based on order of time and the cumulative variability at each data point. **(B)** The residue-wise loadings indicating PC1 and PC2 based on the MEV-TLR4 complex. The graphs are shown in black and blue, respectively. **(C)** The dynamical cross-correlation map generated based on the MEV-TLR4 complex. The blue regions indicate the residues moving in a singular direction, while the pink regions indicate that the residues moved in opposite directions.

**(A)**


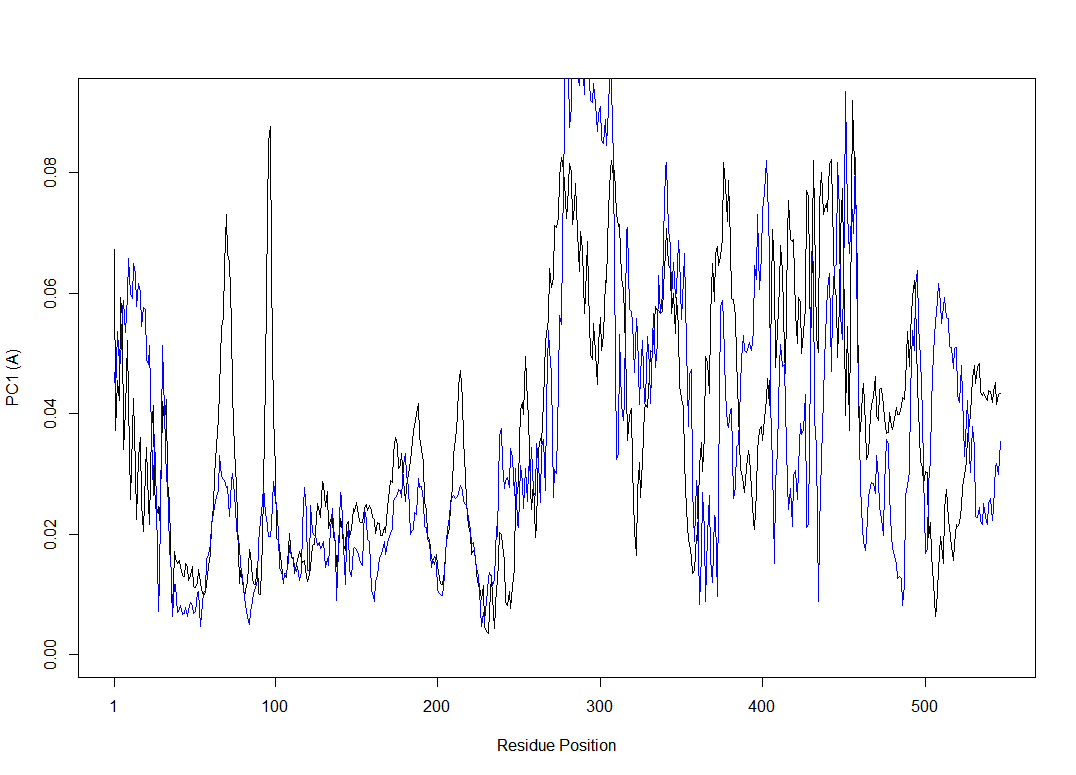


**(B)**


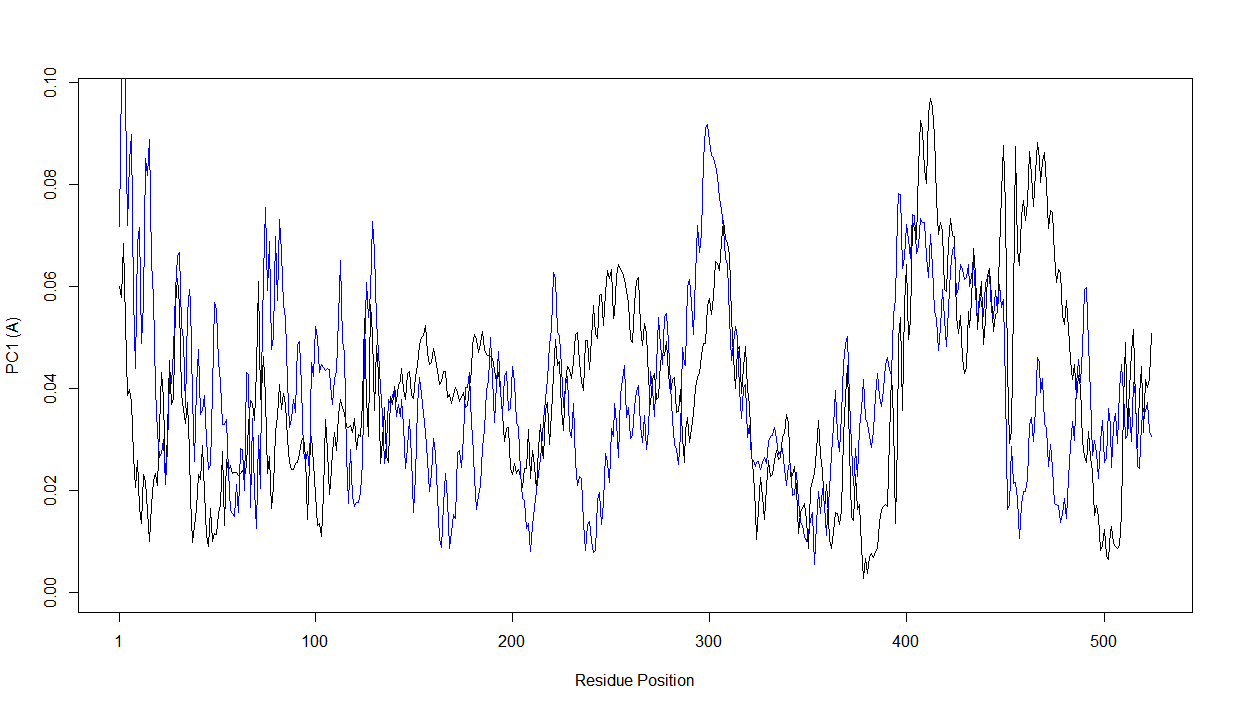


**Supplementary Figure 15.** The residue-wise loadings indicating PC1 and PC2. The graphs are shown in black and blue respectively. (A) The graphs based on the first construct. (B) The graphs based on the second construct.
